# Correction: Validation of the Unesp-Botucatu composite scale to assess acute postoperative abdominal pain in sheep (USAPS)

**DOI:** 10.1371/journal.pone.0268305

**Published:** 2022-05-05

**Authors:** Nuno Emanuel Oliveira Figueiredo Silva, Pedro Henrique Esteves Trindade, Alice Rodrigues Oliveira, Marilda Onghero Taffarel, Maria Alice Pires Moreira, Renan Denadai, Paula Barreto Rocha, Stelio Pacca Loureiro Luna

Following the publication of this article [1] concerns were raised about the statistical analysis presented in this article. The journal has assessed these concerns with the help of a member of the Editorial Board and a statistical adviser, and concluded that although there were some issues with the reporting of the statistics, the overall results and conclusions of the article are supported. In order to update the reporting of the statistical analysis of this study, the following paragraphs are added:

The following paragraph is added to the Discussion section after paragraph 10:

The Kaiser criterion has limitations and therefore, the data was re-evaluated to determine the optimal number of dimensions to retain by applying Horn’s Parallel Analysis [2]. In addition, three other methods were used to confirm comprehensiveness or not of the results of the article. The three methods employed were the Marchenko-Pastur limit [3], the Gavish-Donoho method [4], and the Elbow method [5]. All these methods indicated retaining only one dimension, except for the Elbow method, which indicated retaining two dimensions. Therefore, as most statistical methods to determine the optimal number of dimensions to be retained coincided with the number of dimensions that were previously reported in the article, the results were unchanged.

The following paragraph is added to the subheading Internal consistency of the Results section after paragraph 1:

Cronbach’s α coefficient is considered one of the most popular methods for checking internal consistency. However, one of its limitations is the tau-equivalent. Therefore, to confirm the internal consistency of USAPS, the data was re-evaluated by applying the McDonald’s omega coefficient (ω) [6]. Because we assumed that USAPS was unidimensional, we used McDonald’s total omega coefficient [7]. Internal consistency with all items together was α = 0.81 and ω = 0.85, when excluding interaction α = 0.73 and ω = 0.80, excluding locomotion α = 0.74 and ω = 0.81, excluding head position α = 0.77 and ω = 0.83, excluding posture α = 0.80 and ω = 0.86, excluding activity α = 0.70 and ω = 0.81, and excluding appetite α = 0.88 and ω = 0.87. The McDonald’s omega coefficient results were close to those found by the Cronbach’s alpha coefficient originally used in the study and the classification of the interpretation actually improved from ’acceptable’ (α) to ’strong’ (ω) [7]. These findings confirm the robustness of the USAPS internal consistency.

The following paragraph is added to the subheading Criterion validity of the Results section after paragraph 1:

By a qualitative visual judgment, the dispersion of the relationship between USAPS and Numeric Scale is apparently non-monotonic. To comprehensively check the criterion validity result, the data was re-evaluated by applying four different methods to infer interdependencies between the USAPS and the Numeric Scale. First, Pearson’s correlation coefficient (rP) was applied, as suggested by Streiner et al. [8], to compare with the results given by Spearman’s rank correlation coefficient (rS). The correlation between USAPS and Numeric Scale showed values of rS = 0.83 and rP = 0.84. Therefore Pearson´s correlation did not change the interpretation of the Spearman´s one. Second, the contingency table built between USAPS and Numeric Scale was submitted to a chi-square test (χ^2^). This analysis evidenced the existence of a relationship between the variables (χ^2^ = 1741.6; df = 108; p< 2.2^−16^), according to S7 Table.

Third and fourth, a linear regression model and a quadratic regression model, respectively, applying USAPS as the predictor variable and Numeric Scale as the predictive variable were conducted. The quadratic modelling proved to be more adjusted than the linear one by the significance found in the analysis of variance (F = 65,206; p = 1,352^−15^) and other parameters described in S8 Table. The beta of the Numeric Scale of the quadratic regression was significant (p < 2.2^−16^), confirming the existence of a quadratic relationship between the variables. In light of this new analysis Fig 5 and its caption are updated. Please see the correct Fig 5 and Fig 5 caption below, assuming a quadratic relationship between both variables.

**Fig 5:**
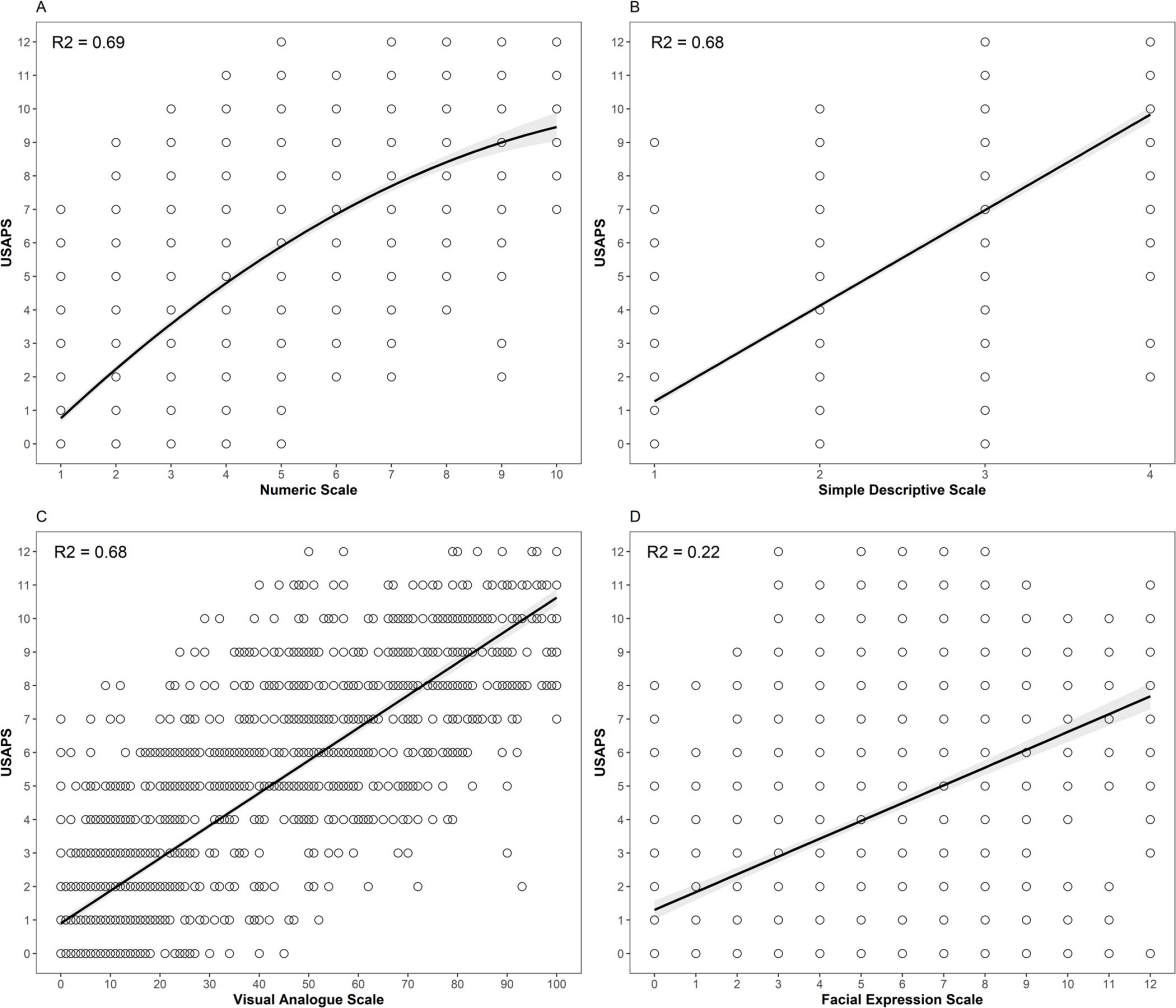
**Scatter plot of the relationship between USAPS (predictor variable) and predictive variable Numeric Scale (A) with quadratic adjustment, and Simple Descriptive Scale (B), Visual Analogue Scale (C), and Facial Expression Scale (D) with linear adjustment.** The line represents the fit line, and the shaded area is the standard error.

In Table 3, the links to the videos in the fourth column require updating. Please see the correct Table 3 with updated links here.

**Table 3:** Final validated Unesp-Botucatu sheep acute composite pain scale (USAPS).

Item	Subitem (descriptors)	Score	Links to videos
**Interaction**	Active, attentive to the environment, interacts and/or follows other animals	0	https://youtu.be/0tmOTmrbTAk
	Apathetic: may remain close to other animals, but interacts little	1	https://youtu.be/qSdTm7Y_ypw
	Very apathetic: isolated or not interacting with other animals, not interested in the environment	2	https://youtu.be/BzPlcdv9RhA
**Locomotion**	Moves about freely, without altered locomotion; when stopped, the pelvic limbs are parallel to the thoracic limbs	0	https://youtu.be/KrRPDLYFSJM
	Moves about with restriction and/or short steps and/or pauses and/or lameness; when stopped, the thoracic or pelvic limbs may be more open and further back than normal	1	https://youtu.be/ynr4b-YRSNo
	Difficulty and/or reluctant to get up and/or not moving and/or walking abnormally and/or limping; may lean against a surface	2	https://youtu.be/YMOuMIk0ubc
**Head position**	Head above the withers or eating	0	https://youtu.be/hz9QDPQ_u50
	Head at the height of the withers	1	https://youtu.be/6pAr9mmJFPk
	Head below the withers (except when eating)	2	https://youtu.be/0Yn6mHRinVs
**Posture**	Arched back		https://youtu.be/Nwuwn9y8y0M
	Extends the head and neck		https://youtu.be/YKG-f83gvik
	Lying down with head resting on the ground or close to the ground		https://youtu.be/kDnXZ_ZIqHc
	Moves the tail quickly (except when breastfeeding) and repeatedly and/or keeps the tail straight (except to defecate/urinate)		https://youtu.be/CnA2M0MlpJc
	Absence of these behaviours	0	
	Presence of one of the related behaviours	1	
	Presence of two or more of the related behaviours	2	
**Activity**	Moves normally	0	https://youtu.be/irQZjNEIa00
	Restless, moves more than normal or lies down and gets up frequently	1	https://youtu.be/F8DwdyJoxZ4
	Moves less frequently or only when stimulated using a stick or does not move	2	https://youtu.be/7keXsYpq5lk
**Appetite**	Normorexia and/or rumination present	0	https://youtu.be/-1gj-WgU_18
	Hyporexia	1	https://youtu.be/4Cv-Hb0-JTA
	Anorexia	2	https://youtu.be/9sIDUwpj4xQ

There is an error in the title for Table 4, “Load values, eigenvalues and variance of the USAPS items based on principal components analysis”. The title should read “Loading values, eigenvalues and variance of the USAPS items based on principal components analysis”. Please find the table with the correct title here.

**Table 4:** Loading values, eigenvalues and variance of the USAPS items based on principal components analysis.

Dimensions	1	2
Items	Loading value	Loading value
Interaction	**0.88**	0.01
Locomotion	**0.85**	-0.14
Head position	**0.78**	0.00
Posture	**0.60**	-0.13
Activity	**0.84**	-0.12
Appetite	0.31	**0.95**
**Eigenvalue**	**3.26**	0.94
**Variance**	**54.25**	15.77

USAPS–Unesp-Botucatu sheep acute composite pain scale. The structure was determined considering items with a loading value ≥ 0.50 or ≤ -0.50 (in bold), with representative dimension (eigenvalue > 1 and variance > 20%) [47].

## Supporting information

S7 Table Contingency table between USAPS (0–12) and Numeric Scale (1–10) submitted to chi-square test. (DOCX)

S8 Table Model findings with linear and quadratic fit of the predictor variable USAPS and with the predictive variable Numeric Scale. (DOCX)
