# Supplementary material for: Validation of the Unesp-Botucatu composite scale to assess acute postoperative abdominal pain in sheep (USAPS)
Source: PLoS One. 2020 Oct 14;15(10):e0239622. doi: 10.1371/journal.pone.0239622 (PMC7556455; doi:10.1371/journal.pone.0239622)
Supplement: S1 Table — (PDF) [file pone.0239622.s001.pdf]

S1 Table. Ethogram with the description of the behaviours analyzed in 48 sheep submitted to laparoscopy [5,11,20-22,34-40].

Behavioural category	Description
Eat	Act of eating feed
Ruminate	Act of ruminating
Drink	Act of drinking water
Urinate	Act of urinating
Defecate	Act of defecating
Normal interaction	Active, attentive to the environment, interacts with and/or follows other animals.
Reduced interaction	Apathetic: may remain close to other animals but interacts little.
Absent interaction	Very apathetic: is isolated or does not follow other animals, is not interested in the environment.
Normal locomotion	Moves about freely, without altered locomotion; when stopped, the pelvic limbs are parallel to the thoracic limbs.
Reduced/altered locomotion	Moves about with restriction and/or short steps and/or pauses and/or lameness with limb support; when stopped.
Absent/abnormal locomotion	Reluctant to get up or gets up with difficulty; does not move or demonstrates unstable or rigid walking and/or limps with little or no limb support; may walk backward or walk in a circle or lean against a surface or fall.
Head above the withers or eating	Occipital is positioned above the withers or eating
Head at the height of the withers	Occipital is positioned at the same level of the withers
Head below the withers (except when eating)	Occipital is positioned below the withers (except when eating)
Standing still in normal posture	Quadrupedal position when not moving
Standing in altered posture	Quadrupedal position with arched back and pelvic limbs rigid and/or caudally extended (pelvic limbs may be more open and further back than normal)
Kick and stamp the limbs on the ground	Kicks the limbs while standing
Lying down with extension of the head and neck and/or limb(s)	Stretches the head and neck and thoracic or pelvic limbs when in recumbence
Lying down	Lying in ventral or lateral recumbence
Lying down with head turned back	Lying down with the head turning back
Lying with head supported on or close to the ground	Lying down with head close or supported on the ground
Quick and repeated tail movements	Energetic tail wagging (except when breastfeeding)
Keep the tail straight (when not urinating or defecating)	Tail stretched (when not urinating or defecating)
Arch the back	Back arching
Body tremors	Back and abdomen (mostly) trembling
Body rotation	Rotates body partially or totally, without getting up
Attention to the affected area	Does not look
	Turns the head and looks
	Licks or tries to lick or avoids contact of the area with surfaces or other animals
