# Supplementary material for: Validation of the Unesp-Botucatu composite scale to assess acute postoperative abdominal pain in sheep (USAPS)
Source: PLoS One. 2020 Oct 14;15(10):e0239622. doi: 10.1371/journal.pone.0239622 (PMC7556455; doi:10.1371/journal.pone.0239622)
Supplement: S2 Table — (PDF) [file pone.0239622.s002.pdf]

S2 Table. Criteria used to select the behaviours included in the pre-refinement of the USAPS used for video analysis (S3 Table), based on content validity and behaviours reported in the literature.

Variable	Criterion	Ev.1	Ev.2	Ev.3	Score	Ethogram	Literature
Interaction	*Active: attentive to the environment, interacts and/or follows other animals*	1	1	1	**1**	x	[5,20,36]
	Apathetic: may remain close to other animals, but interacts little	1	1	1	1	x	[20,36]
	Very apathetic: isolated or does not follow other animals, not interested in the environment	1	1	1	**1**	x	[20,36]
Locomotion	*Moves about freely, without altered locomotion; when stopped, the pelvic limbs are parallel to the thoracic limbs*	1	1	1	**1**	x	[5,20,34,36]
	Moves about with restriction and/or short steps and/or pauses and/or lameness; when stopped, the thoracic or pelvic limbs may be more open and further back than normal	1	1	1	**1**	x	[5,20, 34-36]
	Difficulty and/or reluctant to get up; not moving and/or walking abnormally and/or limping; walks backward or on knees; jumps like a rabbit, walks in a circle; *may lean against a surface*	1	0	1	**0.66**	x	[5,20, 34-36]
Head position	*Head above the withers or eating*	1	0	1	**0.66**	x	[20,34]
	Head at the height of the withers	1	0	1	**0.66**	x	[20,34]
	Head below the withers (except when eating)	1	0	1	**0.66**	x	[20,34]
Posture	Kicks and stamps the limbs on the ground	1	1	1	**1**	x	[20,34-36]
	Extends the head and neck and/or one or more limbs	1	0	1	**0.66**	x	[20,34]
	Body tremors (without considering the ears)	1	1	0	0	x	[20,36]
	The pelvic limbs may be more open and further back than normal	1	1	1	1	x	[5,20,36]
Posture in recumbence	Lying down, relaxed	1	1	1	**1**	x	[20,36]
	Extends neck and body forward	1	1	0	**0.66**	x	[5,20,36]
	Lying down with the head resting on the ground or close to the ground.	1	1	1	**1**		[5,20,34,36]
Miscellaneous behaviours	*Moves the tail quickly and repeatedly (except when breastfeeding) or keeps the tail straight (except when defecating or urinating)*	1	0	1	**0.66**	x	[5,20,36]
	Arched back	1	1	1	**1**	x	[5,20,34,36]
	Rotates body partially or totally, without getting up	1	0	0	0	x	[20,36]
Activity	*Moves normally*	1	1	1	**1**		[5,20,34,35]
	Restless, moves more than normal or lies down and gets up frequently	1	1	1	**1**		[5,20,34,36]
	Moves less frequently or only when stimulated using a stick or does not move	1	1	1	**1**		[20,36]
Appetite	*Normorexia and/or rumination present*	1	1	1	**1**	x	[5,20,36]
	Hyporexia	1	0	1	**0.66**		[5,20,36]
	Anorexia	1	1	1	**1**		[5,20,36]
**Attention to the affected area**	Does not look	1	1	1	**1**	x	[20,36]
	Turns the head and looks	1	1	1	**1**	x	[5,20,36]
	Licks or tries to lick or avoids contact of the area with surfaces or other animals	1	1	1	**1**	x	[5,20,36]

USAPS: Unesp-Botucatu sheep acute composite pain scale; Ev. = Evaluator; each item was classified as relevant (+1), not known (0), or irrelevant (-1), the values were added and divided by the number of experts. Score: final score of content validation. In bold are the items approved in content validation, because the mean score was ≥ 0.5 and because the specific pain-related behavior had been reported in the ethogram (marked with "x") and literature [number of the references in the list of references]; in italics are the behaviors that remained on the scale after refinement.
