# Supplementary material for: Validation of the Unesp-Botucatu composite scale to assess acute postoperative abdominal pain in sheep (USAPS)
Source: PLoS One. 2020 Oct 14;15(10):e0239622. doi: 10.1371/journal.pone.0239622 (PMC7556455; doi:10.1371/journal.pone.0239622)
Supplement: S3 Table — (PDF) [file pone.0239622.s003.pdf]

S3 Table. Pre-refinement of the USAPS to assess postoperative pain in sheep submitted to video analysis after content validation.

Principal item	Subitems (with sub-divisions in some subitems)	video 1	video 2	video 3	video 4
Interaction	A. Active: attentive to the environment, interacts and/or follows other animals				
	B. Apathetic: may remain close to other animals but interacts little				
	C. Very apathetic: isolated or does not follow other animals, not interested in the environment				
Locomotion	A. Moves about freely, without altered locomotion; when stopped, the pelvic limbs are parallel to the thoracic limbs				
	B. Moves with restriction and/or short steps and/or pauses and/or lameness; when stopped, the pelvic limbs may be more open and further back than normal				
	C. 1.1 Reluctant to get up and/or				
	1.2 Gets up with difficulty				
	2.1 Does not move and/or				
	2.2 Walks abnormally and/or				
	2.3 Lameness				
	3.1 Walks backward and/or				
	3.2 Walks in circles and/or				
	3.3 Leans against a surface				
Head position	A. Head above the withers or eating				
	B. Head at the level of the withers				
	C. Head below the withers (except when eating)				
Posture	1. Kicks or stamps one or more limbs on the ground				
	2.1. Extends the head and neck and/or				
	2.2. Extends one or more limbs				
	3. Lying down with the head resting on the ground or close to the ground				
Miscellaneous behaviours	1.1. Moves the tail quickly and repeatedly (except when breastfeeding) and/or				
	1.2. Keeps the tail straight, except when defecating or urinating				
	2. Arched back				
	3. Body tremors (without considering the ears)				
	4. Crawls in ventral recumbence, without getting up				
Activity	A. Moves normally				
	B. Restless, moves more than normal or lies down and gets up often				
	C. Moves less frequently or only when stimulated using a stick or does not move				
Appetite	A1. Normorexia and/or				
	A2. Rumination present				
	B. Hyporexia.				
	C. Anorexia				

USAPS: Unesp-Botucatu sheep acute composite pain scale.
