# Supplementary material for: Validation of the Unesp-Botucatu composite scale to assess acute postoperative abdominal pain in sheep (USAPS)
Source: PLoS One. 2020 Oct 14;15(10):e0239622. doi: 10.1371/journal.pone.0239622 (PMC7556455; doi:10.1371/journal.pone.0239622)
Supplement: S4 Table — (PDF) [file pone.0239622.s004.pdf]

S4 Table. Refinement process for inclusion and exclusion of items and subitems on the USAPS.

Items / Tests	Sum	Etho* M2	CV*	%M2 ≥ 15*	PCA	Intra*	Inter*	Resp	ITC	IC	*Sp* and *S*
**Interaction**	**8**		1		1	1	1	1	1	1	1
**Locomotion**	**8**		1		1	1	1	1	1	1	1
**Locomotion 1**	**3**			1		1	1				
**Locomotion 2**	**3**			1		1	1				
**Locomotion 3**	**3**			1		1	1				
**Head position**	**7**		1		1	1	1		1	1	1
**Posture**	2					1			1		
Posture A	2			1			1				
**Posture B1**	**3**	1	1	1							
Posture B2	2		1	1							
**Posture C**	**4**		1	1		1	1				
**Miscellaneous behaviours**	4						1	1	1	1	
**Miscellaneous A**	**3**		1	1			1				
**Miscellaneous B**	**3**	1	1	1							
Miscellaneous C	0										
Miscellaneous D	2					1	1				
**Activity**	**7**		1		1		1	1	1	1	1
**Appetite**	**7**		1		1	1	1	1	1	1	

USAPS: Unesp-Botucatu sheep acute composite pain scale. **Statistical tests according to Table 1**: **Etho. M2** - highest score of behaviour in the ethogram according to the Friedman test at M2 (moment of pain) vs the other moments; **% M2 ≥ 15** - at least 15% frequency of occurrence of items/subitems at M2; **CV** - content validation; **Intra** – intra-observer reliability; **Inter** – inter-observer reliability; **PCA** - Principal component analysis (> 0.5); **Resp** (responsiveness) - highest score of the behaviour at M2 vs at least two of the three moments (M1, M3 and M4) according to Friedman test; **ITC** - item-total Spearman correlation between 0.3 – 0.7; **IC** - Internal consistency (> 0.6); ***Sp*** – Specificity (≥ 70%); ***S*** – Sensitivity (≥ 70%). **Sum** – the sum of the 10 statistical tests. The main items were subjected to ten tests and when approved at least in five, they were included in the final scale (Table 3); the subitems were subjected to five tests marked with asterisk (*) and when approved in at least three, they were included in the final scale (Table 3). Numbers 1 indicates that the item /subitem was included according to the criteria of each test. The items and subitems included in the final scale after refinement are in bold.

Locomotion: 1 - Reluctant to get up or gets up with difficulty; 2 - Does not move and/or walks abnormally and/or with lameness; 3 - Leans against a surface; **Posture**: A - Kicks or stamps one or more limbs on the ground; B1 - Extends the head and neck; B2 - Extends one or more limbs; C – Lying down with the head resting on the ground or close to the ground; **Miscellaneous behaviours**: A - Moves the tail quickly and repeatedly (except when breastfeeding) or keeps the tail straight (except when defecating or urinating); B - Arched back; C - Body tremors (without considering the ears); D. Crawls in ventral recumbence, without getting up. Moments - M1: preoperative; M2: postoperative, before rescue analgesia; M3: postoperative, after rescue analgesia; M4: 24h postoperative.
