# Supplementary material for: Validation of the Unesp-Botucatu composite scale to assess acute postoperative abdominal pain in sheep (USAPS)
Source: PLoS One. 2020 Oct 14;15(10):e0239622. doi: 10.1371/journal.pone.0239622 (PMC7556455; doi:10.1371/journal.pone.0239622)
Supplement: S5 Table — (PDF) [file pone.0239622.s005.pdf]

S5 Table. Inter-observer matrix agreement of items of the USAPS, unidimensional scales and rescue analgesia indication.

Evaluator	1			2			3			1			2			3		
	k_w	Min	Max	k_w	Min	Max	k_w	Min	Max	k_w	Min	Max	k_w	Min	Max	k_w	Min	Max
	**Interaction**									**Locomotion**								
**2**	**0.54**	0.47	0.61							**0.52**	0.46	0.59						
**3**	**0.53**	0.46	0.61	**0.62**	0.58	0.66				**0.46**	0.38	0.54	**0.55**	0.48	0.63			
**4**	**0.59**	0.52	0.66	**0.51**	0.44	0.58	**0.52**	0.45	0.58	**0.61**	0.55	0.68	**0.50**	0.42	0.58	**0.49**	0.41	0.58
	**Head position**									**Posture**								
**2**	**0.58**	0.51	0.65							0.41	0.26	0.55						
**3**	0.46	0.37	0.56	**0.53**	0.45	0.61				0.34	0.14	0.53	0.37	0.17	0.58			
**4**	**0.50**	0.42	0.58	0.48	0.4	0.56	0.43	0.35	0.52	0.33	0.17	0.5	0.49	0.39	0.59	0.32	0.07	0.57
	**Activity**									**Appetite**								
**2**	**0.54**	0.48	0.6							**0.53**	0.44	0.61						
**3**	0.41	0.33	0.5	**0.57**	0.51	0.63				**0.53**	0.44	0.62	**0.54**	0.46	0.62			
**4**	**0.59**	0.53	0.65	0.49	0.42	0.57	0.45	0.37	0.53	0.25	0.09	0.41	0.33	0.19	0.46	0.24	0.07	0.4
	**NS**									**SDS**								
**2**	0.40	0.13	0.68							**0.68**	0.68	0.68						
**3**	0.44	0.32	0.55	**0.69**	0.69	0.69				**0.52**	0.44	0.59	**0.59**	0.59	0.59			
**4**	**0.72**	0.72	0.72	0.46	0.18	0.74	0.49	0.17	0.8	**0.67**	0.64	0.7	**0.65**	0.62	0.69	0.47	0.37	0.57
	**RA**																	
**2**	**0.51**	0.43	0.59															
**3**	0.49	0.4	0.57	**0.65**	0.57	0.73												
**4**	**0.55**	0.47	0.63	0.47	0.39	0.55	0.43	0.35	0.51									
	**ICC**	Min	Max	**ICC**	Min	Max	**ICC**	Min	Max	**ICC**	Min	Max	**ICC**	Min	Max	**ICC**	Min	Max
	**USAPS**									**VAS**								
**2**	**0.65**	0.59	0.71							0.24	0.14	0.33						
**3**	**0.57**	0.50	0.63	**0.74**	0.69	0.78				0.33	0.24	0.42	0.64	0.58	0.70			
**4**	**0.70**	0.64	0.75	**0.63**	0.56	0.68	**0.53**	0.46	0.60	**0.70**	0.64	0.74	0.40	0.32	0.48	0.46	0.38	0.53

USAPS - Unesp-Botucatu sheep acute composite pain scale; RA - Rescue analgesia; NS – numerical; SDS - simple descriptive; VAS - visual analogue. k_w – weighted kappa coefficient; ICC - intraclass correlation coefficient; CI - Confidence interval. Interpretation of reliability: very good 0.81 - 1.0; good 0.61 - 0.80; moderate 0.41 - 0.60; reasonable 0.21 - 0.4; poor < 0.2 [29,52,53]. Bold type corresponds to values > 0.50.
