# Supplementary material for: Validation of the Unesp-Botucatu composite scale to assess acute postoperative abdominal pain in sheep (USAPS)
Source: PLoS One. 2020 Oct 14;15(10):e0239622. doi: 10.1371/journal.pone.0239622 (PMC7556455; doi:10.1371/journal.pone.0239622)
Supplement: S6 Table — Scores, specificity, sensitivity and Youden index corresponding to rescue analgesia indication of the USAPS and unidimensional scales (A); 95% confidence intervals of 1,001 replications and of sensitivity and specificity >0.90 applied to estimate the diagnostic uncertainty zone of the cut-off point of each scale, according to the Youden index (B). (PDF) [file pone.0239622.s006.pdf]

S6 Table. Scores, specificity, sensitivity and Youden index corresponding to rescue analgesia indication of the USAPS and unidimensional scales (A); 95% confidence intervals of 1,001 replications and of sensitivity and specificity >0.90 applied to estimate the diagnostic uncertainty zone of the cut-off point of each scale, according to the Youden index (B).

**(A)**	**USAPS**			**NS**				**SDS**				**VAS**			
**Score**	***Sp***	***S***	***YI***	**Score**	***Sp***	***S***	***YI***	**Score**	***Sp***	***S***	***YI***	**Score**	***Sp***	***S***	***YI***
0	0	1	0	1	0	1	0	1	0	1	0	0	0	1	0
1	0.42	0.99	0.41	2	0.59	1.00	0.59	**2**	**0.84**	**0.99**	**0.83**	1	0.28	1	0.28
2	0.58	0.97	0.55	3	0.84	0.99	0.83	3	1	0.69	0.69	2	0.30	1	0.30
**3**	0.79	0.95	0.74	**4**	**0.97**	**0.93**	**0.90**	4	1	0.17	0.17	(…)	…	…	…
**4**	0.88	0.92	0.80	5	1	0.77	0.77					24	0.91	0.96	0.87
5	0.93	0.85	0.78	6	1	0.58	0.58					25	0.93	0.95	0.87
6	0.97	0.73	0.69	7	1	0.41	0.41					**26**	**0.94**	**0.94**	**0.88**
7	0.99	0.57	0.56	8	1	0.26	0.26					27	0.95	0.93	0.88
8	1	0.43	0.43	9	1	0.10	0.10					28	0.95	0.92	0.87
9	1	0.29	0.28	10	1	0.04	0.04					(…)	…	…	…
10	1	0.16	0.16									98	1	0.03	0.03
11	1	0.07	0.07									99	1	0.02	0.02
12	1	0.01	0.01									100	1	0.01	0.01
**(B)**	Confidence interval				Confidence interval				Confidence interval				Confidence interval		
	**Est.**	Min.	Max.		**Est.**	Min.	Max.		**Est.**	Min.	Max.		**Est.**	Min.	Max.
**Replication CI**	**3.5**	3.5	4.5		**3.5**	3.5	3.5		**1.5**	1.5	1.5		**25.5**	20.5	26.5
**CI > 0.90**	-	3.8	4.0		**--**	3.0	3.7		**--**	1.8	1.9		**--**	22	29
**AUC**	**0.96**	0.94	0.96		**0.99**	0.99	0.99		**0.97**	0.97	0.98		**0.99**	0.98	0.99

USAPS: Unesp-Botucatu sheep acute composite pain scale; NS – numerical; SDS - simple descriptive; VAS - visual analogue; *Sp*- Specificity; *S* - Sensitivity; *YI* - Youden Index; CI - confidence interval; CI > 0.90 - CI of specificity and sensitivity > 0.90. AUC - area under the curve; Est - estimated; Min - minimum; Max – maximum; Values in bold are in the same line of the cut-off point for each scale (A) and estimated values (B).
