# Supplementary material for: Validation of the Unesp-Botucatu composite scale to assess acute postoperative abdominal pain in sheep (USAPS)
Source: PLoS One. 2020 Oct 14;15(10):e0239622. doi: 10.1371/journal.pone.0239622 (PMC7556455; doi:10.1371/journal.pone.0239622)
Supplement: Attachment — Submitted filename: PONE-D-20-07213_reviewerUSAPS.pdf [file pone.0239622.s008.pdf]

PLOS ONE

Validation of the Unesp-Botucatu sheep acute composite pain scale (USAPS)

--Manuscript Draft--

**Manuscript Number:**	PONE-D-20-07213
**Article Type:**	Research Article
**Full Title:**	Validation of the Unesp-Botucatu sheep acute composite pain scale (USAPS)
**Short Title:**	Validation of the Unesp-Botucatu sheep acute pain scale (USAPS)
**Corresponding Author:**	Stelio Pacca Loureiro Luna<br>São Paulo State University (Unesp), School of Veterinary Medicine and Animal Science<br>Botucatu, São Paulo BRAZIL
**Keywords:**	ovine; welfare; analgesia; laparoscopy; behaviour; reliability; validity.
**Abstract:**	A scale with robust statistical validation is essential to diagnose pain and guarantee effective analgesia. This blind, randomized, prospective and opportunist study aimed to develop an ethogram to evaluate behavior and validate a scale to assess acute ovine postoperative pain. Elective laparoscopy was performed in 48 healthy sheep, filmed at one preoperative and three postoperative moments, before and after analgesic rescue and 24 hours after. The videos were randomized and assessed twice by four evaluators, with a one-month interval between evaluations. Statistical analysis was performed using R software and differences were considered significant when p <0.05. The intra- and inter-observer reliability ranged from moderate to very good (intraclass correlation coefficient ≥0.55). The scale presented Spearman correlations > 0.80 with the numerical, simple descriptive, and visual analogue scales, and a correlation of 0.59 with the facial expression scale. According to the Friedman test, the scale was responsive, due to the increase and decrease in pain scores of all items after surgery and analgesic intervention, respectively. Based on the multiple association, a unidimensional scale was adopted. All items on the scale demonstrated an acceptable Spearman item-total correlation (0.3-0.7). The internal consistency was excellent (Cronbach's α = 0.84) and all items presented specificity >0.70 and sensitivity between 0.67-0.85, except for appetite. According to the Youden index, the cut-off point was ≥ 4 out of 12, with a diagnostic uncertainty zone of 3 to 4, which indicates the accuracy of the scale. The area under the curve >0.95 for all evaluators demonstrated excellent discriminatory capacity of the instrument. Total scores of the scale were classified as mild (0-3), moderate (4-8) and severe (≥9) pain. In conclusion, the Unesp-Botucatu sheep acute composite pain scale (USAPS) is valid, reliable, specific, sensitive, with excellent internal consistency, accuracy, discriminatory capacity, and a defined cut-off point.
**Order of Authors:**	Nuno Emanuel Oliveira Figueiredo Silva
	Pedro Henrique Esteves Trindade
	Alice Rodrigues Oliveira
	Marilda Onghero Taffarel
	Maria Alice Pires Moreira
	Renan Denadai
	Paula Barreto Rocha
	Stelio Pacca Loureiro Luna
**Additional Information:**	
**Question**	**Response**
**Financial Disclosure**<br><br>Enter a financial disclosure statement that	SPLL - Financial support from the São Paulo Research Foundation – FAPESP (Process 2010/08967-0 and 2017/12815-0). http://www.fapesp.br/en/<br>NEOFS - Grant from Coordenação de Aperfeiçoamento de Pessoal de Nível Superior

Powered by Editorial Manager® and ProduXion Manager® from Aries Systems Corporation

describes the sources of funding for the work included in this submission. Review the submission guidelines for detailed requirements. View published research articles from *PLOS ONE* for specific examples.<br><br>This statement is required for submission and **will appear in the published article** if the submission is accepted. Please make sure it is accurate.<br><br>**Unfunded studies**<br>Enter: *The author(s) received no specific funding for this work.*<br><br>**Funded studies**<br>Enter a statement with the following details:<br>• Initials of the authors who received each award<br>• Grant numbers awarded to each author<br>• The full name of each funder<br>• URL of each funder website<br>• Did the sponsors or funders play any role in the study design, data collection and analysis, decision to publish, or preparation of the manuscript?<br>• **NO** - Include this sentence at the end of your statement: *The funders had no role in study design, data collection and analysis, decision to publish, or preparation of the manuscript.*<br>• **YES** - Specify the role(s) played.<br><br>* typeset	– Brazil (CAPES)<br>The funders had no role in study design, data collection and analysis, decision to publish, or preparation of the manuscript.
**Competing Interests**<br><br>Use the instructions below to enter a competing interest statement for this submission. On behalf of all authors, disclose any competing interests that could be perceived to bias this work—acknowledging all financial support and any other relevant financial or non-financial competing interests.<br><br>This statement **will appear in the published article** if the submission is accepted. Please make sure it is accurate. View published research articles from *PLOS ONE* for specific examples.	The authors have declared that no competing interests exist

Powered by Editorial Manager® and ProduXion Manager® from Aries Systems Corporation

**NO authors have competing interests**<br><br>Enter: *The authors have declared that no competing interests exist.*<br><br>**Authors with competing interests**<br><br>Enter competing interest details beginning with this statement:<br><br>*I have read the journal's policy and the authors of this manuscript have the following competing interests: [insert competing interests here]*<br><br>* typeset	
**Ethics Statement**<br><br>Enter an ethics statement for this submission. This statement is required if the study involved:<br><br>• Human participants<br>• Human specimens or tissue<br>• Vertebrate animals or cephalopods<br>• Vertebrate embryos or tissues<br>• Field research<br><br>Write "N/A" if the submission does not require an ethics statement.<br><br>General guidance is provided below. Consult the submission guidelines for detailed instructions. **Make sure that all information entered here is included in the Methods section of the manuscript.**	The study was approved by the Ethical Committee for the Use of Animals in Research from the School of Veterinary Medicine and Animal Science, São Paulo State University (Unesp), Botucatu, São Paulo, Brazil, under protocol 0027/2017 and follows the Brazilian Federal legislation of CONCEA (National Council for the Control of Animal Experimentation).

Powered by Editorial Manager® and ProduXion Manager® from Aries Systems Corporation

**Format for specific study types**<br><br>**Human Subject Research (involving human participants and/or tissue)**<br>• Give the name of the institutional review board or ethics committee that approved the study<br>• Include the approval number and/or a statement indicating approval of this research<br>• Indicate the form of consent obtained (written/oral) or the reason that consent was not obtained (e.g. the data were analyzed anonymously)<br><br>**Animal Research (involving vertebrate animals, embryos or tissues)**<br>• Provide the name of the Institutional Animal Care and Use Committee (IACUC) or other relevant ethics board that reviewed the study protocol, and indicate whether they approved this research or granted a formal waiver of ethical approval<br>• Include an approval number if one was obtained<br>• If the study involved *non-human primates*, add *additional details* about animal welfare and steps taken to ameliorate suffering<br>• If anesthesia, euthanasia, or any kind of animal sacrifice is part of the study, include briefly which substances and/or methods were applied<br><br>**Field Research**<br><br>Include the following details if this study involves the collection of plant, animal, or other materials from a natural setting:<br>• Field permit number<br>• Name of the institution or relevant body that granted permission	
**Data Availability**<br><br>Authors are required to make all data underlying the findings described fully available, without restriction, and from the time of publication. PLOS allows rare exceptions to address legal and ethical concerns. See the PLOS Data Policy and FAQ for detailed information.	Yes - all data are fully available without restriction

A Data Availability Statement describing where the data can be found is required at submission. Your answers to this question constitute the Data Availability Statement and **will be published in the article**, if accepted.<br><br>**Important:** Stating 'data available on request from the author' is not sufficient. If your data are only available upon request, select 'No' for the first question and explain your exceptional situation in the text box.<br><br>Do the authors confirm that all data underlying the findings described in their manuscript are fully available without restriction?	
**Describe where the data may be found in full sentences. If you are copying our sample text, replace any instances of XXX with the appropriate details.**<br><br>• If the data are **held or will be held in a public repository**, include URLs, accession numbers or DOIs. If this information will only be available after acceptance, indicate this by ticking the box below. For example: *All XXX files are available from the XXX database (accession number(s) XXX, XXX.).*<br>• If the data are all contained **within the manuscript and/or Supporting Information files**, enter the following: *All relevant data are within the manuscript and its Supporting Information files.*<br>• If neither of these applies but you are able to provide **details of access elsewhere**, with or without limitations, please do so. For example:<br><br>*Data cannot be shared publicly because of [XXX]. Data are available from the XXX Institutional Data Access / Ethics Committee (contact via XXX) for researchers who meet the criteria for access to confidential data.*<br><br>*The data underlying the results presented in the study are available from (include the name of the third party*	All relevant data are within the manuscript and its Supporting Information files.

and contact information or URL).<br>• This text is appropriate if the data are owned by a third party and authors do not have permission to share the data.<br><br>* typeset	
Additional data availability information:	Tick here if the URLs/accession numbers/DOIs will be available only after acceptance of the manuscript for publication so that we can ensure their inclusion before publication.

Powered by Editorial Manager® and ProduXion Manager® from Aries Systems Corporation

Manuscript Click here to download Manuscript Manuscript.docx

Validation of the Unesp-Botucatu sheep acute composite pain scale (USAPS)

Nuno Emanuel Oliveira Figueiredo Silva[1¶], Pedro Henrique Esteves Trindade[1¶], Alice Rodrigues Oliveira[1&], Marilda Onghero Taffarel[3&], Maria Alice Pires Moreira[4&], Renan Denadai[2&], Paula Barreto Rocha[1&], Stelio Pacca Loureiro Luna[1¶*]

[1] Department of Anesthesiology, School of Medicine, São Paulo State University (Unesp), Botucatu, São Paulo, Brazil

[2] Department of Veterinary Surgery and Animal Reproduction, School of Veterinary Medicine and Animal Science, São Paulo State University (Unesp), Botucatu, São Paulo, Brazil.

[3] Department of Veterinary Medicine, State University of Maringá, Umuarama, Paraná, Brazil.

[4] Goiano Federal Institute, Urutaí Campus, Department of Veterinary Medicine, Urutaí, GO, Brazil.

[¶]These authors contributed equally to this work.

[&]These authors also contributed equally to this work.

* Corresponding and Senior author

Email: stelio.pacca@unesp.br

Abstract

A scale with robust statistical validation is essential to diagnose pain and guarantee effective analgesia. This blind, randomized, prospective and opportunist study aimed to develop an ethogram to evaluate behavior and validate a scale to assess acute ovine postoperative pain. Elective laparoscopy was performed in 48 healthy sheep, filmed at one preoperative and three postoperative moments, before and after analgesic rescue and 24 hours after. The videos were randomized and assessed twice by four evaluators, with a one-month interval between

evaluations. Statistical analysis was performed using R software and differences were considered significant when p <0.05. The intra- and inter-observer reliability ranged from moderate to very good (intraclass correlation coefficient ≥0.55). The scale presented Spearman correlations > 0.80 with the numerical, simple descriptive, and visual analogue scales, and a correlation of 0.59 with the facial expression scale. According to the Friedman test, the scale was responsive, due to the increase and decrease in pain scores of all items after surgery and analgesic intervention, respectively. Based on the multiple association, a unidimensional scale was adopted. All items on the scale demonstrated an acceptable Spearman item-total correlation (0.3-0.7). The internal consistency was excellent (Cronbach's α = 0.84) and all items presented specificity >0.70 and sensitivity between 0.67-0.85, except for appetite. According to the Youden index, the cut-off point was ≥ 4 out of 12, with a diagnostic uncertainty zone of 3 to 4, which indicates the accuracy of the scale. The area under the curve >0.95 for all evaluators demonstrated excellent discriminatory capacity of the instrument. Total scores of the scale were classified as mild (0-3), moderate (4-8) and severe (≥9) pain. In conclusion, the Unesp-Botucatu sheep acute composite pain scale (USAPS) is valid, reliable, specific, sensitive, with excellent internal consistency, accuracy, discriminatory capacity, and a defined cut-off point.

Introduction

The lack of valid and reliable instruments to recognize and quantify pain in farm animals compromises their welfare state and limits the use of analgesics in these species [1–4]. The conviction that these animals feel less pain compared to small animals [5], economic reasons [6,7], the need for withholding periods for the consumption of milk and meat due to residues of the drugs, and the absence of licensed analgesics are other factors that make it difficult to treat pain in farm animals [3].

Sheep are subjected to several painful procedures, often without appropriate use of anesthesia or analgesia, such as castration, tail cutting, mulesing, and ear marking [8]. Pain

in sheep also occurs from diseases such as mastitis, pododermatitis, abscesses in the feet, and external myiasis [9–11], which can lead to chronic and neuropathic pain [12–14].

The sheep species is suitable as an experimental model due to its similarity in size and weight to humans. In 2014, 60,209 sheep were used in research in the European Union, an increase of 108% compared to 2011, only surpassed by pigs among farm animals. Sheep and pigs are more commonly used than dogs and non-human primates as non-rodent models for research and teaching, as their limitations to using these other species as models, including ethical issues [15,16]. Among the experimental models that include sheep are preclinical and translational studies on osteoporosis [17] and bone regeneration and osteointegration of dental implants [18].

To treat pain, instruments are needed to assess it. Although there are several useful indicators to assess nociception in experimental situations, such as the injection of formalin into the interdigital space [19], von Frey filaments [20], tourniquets [21], electrical stimuli [22], and pneumo-mechanical stimulus in limbs [23], these are not reproducible and are difficult to use in clinical situations. Actigraphy can be used to monitor sheep from a distance, which excludes the presence of the observer, however, this method requires specific equipment [24]. Other physiological measures, such as hair cortisone concentration [25], heart rate variability [26], blood pressure, ocular and rectal temperature, electromyography, and electroencephalography, are not clinically feasible and require the animal to be restrained [27].

The behavioral expression of pain replaces the absence of verbal expression of the animals. Behavior is easy to observe, does not require restraint of the animal in field situations, and, therefore, does not generate stress, does not require equipment, and has no cost, thus representing the most applicable method in both clinical or experimental situations [28–30].

In contrast to cattle and goats, sheep, when suffering pain under restraint, tend to remain more silent [28] and only express pain behaviors when released [31]. These behaviors are: reduced interaction with the environment and with other animals, gait abnormalities, lameness, relaxation of the pelvic limbs (which move slowly and alternately when the animals are stationary), turning of the head, hyporexia, abnormal vocalization, lip-licking movement,

curved lips, gnashing of teeth, tremors, stamping feet on the ground, and strong tail wagging [8,28,32,33].

Two analyzes are essential to develop and validate a pain scale: validity, which indicates whether the instrument effectively measures the attribute for which it was designed [34–36], and reliability, which guarantees equivalence of results when the measure is evaluated by the same observer on different occasions, or by different observers on the same occasion [36–38]. The scale must also be responsive, meaning its scores change according to a painful stimulus or analgesia [39–41]. Behavior-based pain scales have been developed in dogs [34,42,43],[44] cats [41,45–47], horses [38,40,48–50], cattle [29], and pigs [30]. The most commonly used scales to measure postoperative pain in sheep are still unidimensional, such as the numerical (NS), simple descriptive (SDS), and visual analogue scales (VAS). However, these instruments exclusively evaluate the intensity of pain dor [39], whereas multidimensional or composite scales include sensory, motor and emotional qualities [45,47].

To develop the scales, a species-specific ethogram is produced to quantifies the duration and/or frequency of the behaviors present before and after a painful stimulus [51]. Although previous studies have reported the behavioral descriptors of pain based on an ethogram after a nociceptive stimulus [8, 28,31,32,52–66], according to our knowledge, there are no validated behavioral scales in the literature to detect acute pain in sheep, following solid statistical analysis [67,68]. The instruments developed to evaluate acute pain in sheep were based on behavioral changes in lambs submitted to orchiectomy and tail cutting [28] or facial expression in sheep with pododermatitis and mastitis [69]. Another facial scale (sheep grimace) was published after the beginning of our research [70]. None of these studies evaluated the criteria, content, and construct validity. To guarantee the reliable measurement of pain, it is necessary to develop an instrument with in-depth statistical validation, such as that performed in in cats [47], cattle [29] horses [38], and pigs [30], which used a blind and random methodology, with evidence of validity, reliability, sensitivity, specificity, and a defined analgesic intervention point [71].

Given the hypothesis that the scale proposed in the current study presents reliability, and content, construct and criterion validities, the main objective of this study was to validate a behavioral scale to assess acute pain in sheep undergoing soft tissue surgery (laparoscopy), constructed from the literature and an ethogram, followed by refinement and subsequent validation, with definition of the cut-off point for analgesic intervention.

Material and methods

This was a blind, randomized, prospective and opportunist study. The study was approved by the Ethical Committee for the Use of Animals in Research from the School of Veterinary Medicine and Animal Science, São Paulo State University (Unesp), Botucatu, São Paulo, Brazil, under protocol 0027/2017 and follows the Brazilian Federal legislation of CONCEA (National Council for the Control of Animal Experimentation). The study followed the recommendations of ARRIVE [72]. In total, 48 sheep (*Ovis aries* species, dairy line) were used, from the institution itself, of the breeds Bergamácia, Lacaune, and Dorper, 3.5 ± 1.8 (1.5 - 6) years old and weighing 58.5 ± 17.3 (34 - 92) kg. As inclusion criteria, the sheep were required to be considered healthy through clinical and laboratory evaluation (hematocrit, plasma protein, glucose and lactate). The sheep were placed in stalls, close to the pen they lived in and where they were used to stay like a shelter when atmospheric conditions were extrem, 24 hours before the start of the study, during which they fasted for feed, and for 12 hours they fasted for water. In each stall (3 x 2 x 1.10 m or 2.20 x 2 x 1.20 m - length x width x height) 6 to 8 sheep or 2 to 4 sheep were housed, respectively.

For the pilot study, sheep were filmed and evaluated before and after laparoscopy, to test the position of the cameras and other adjustments, in order to guarantee the quality of filming. For the main study, the following moments were selected: M1 - one hour before surgery; M2 - at the predicted time of greatest pain, between three and four hours after the end of surgery; M3 - one hour after analgesic intervention; and M4 - 24 hours after surgery.

Immediately before surgery, 30,000 IU/kg of benzathine penicillin (Pentabiótico®, Zoetis, São Paulo, SP, Brazil) was administered intramuscularly (IM). After dissociative anesthesia with 0.5 mg/kg of diazepam (Compaz®, Cristália, Itapira, SP, Brazil) and 5 mg/kg of ketamine (Cetamin®, Syntec; Santana de Parnaíba, SP, Brazil) administered intravenously (IV), lumbosacral epidural anesthesia was performed with 0.1 ml/kg of 1% lidocaine without vasoconstrictor (Xylestesin®, Cristália, Itapira, SP, Brazil) and anesthetic infiltration with 2% lidocaine without vasoconstrictor (Xylestesin®, Cristália, Itapira, SP, Brazil) at the incision site and subsequent introduction of a trocar. When the animals demonstrated any sympathetic response related to the surgery, characterized by an increase of more than 20% in heart rate in relation to the value observed before the beginning of the surgery, dissociative anesthesia was supplemented with 5 mg/kg of ketamine IV.

In all animals, the same experienced surgeon performed a laparoscopy for follicular aspiration and replacement of follicular cells [73–75], by inserting three trocars (5mm) in three retro-umbilical regions. The postoperative analgesic intervention was performed after the M2 evaluation, with 0.5 mg/kg meloxicam 2% (Maxicam®, Ourofino, Cravinhos, SP, Brazil) and 0.2 mg/kg morphine (Dimorf®, Cristália, Itapira, SP, Brazil) IV in separate syringes (Fig 1).

Fig 1. Timeline of moments for validation of the Unesp-Botucatu sheep acute composite pain scale (USAPS).

Data collection

Three to four animals underwent surgery per day. The procedures started at 9 am and the evaluations of the last animals ended around 7 pm. The study was carried out in the months of April and May 2017 and the mean temperature and humidity of the environment varied between 16 - 24ºC and 68 - 92 %, respectively. The local had the following geographic coordinates: latitude - 22º51’ S; longitude - 48º26’ O; altitude - 818 m. The presential observer made the recordings using a digital camera (Gopro Hero5 Black®) positioned on a tripod. The

camera was turned on and the presential researcher left the place, and stayed at least 10 m in order to minimize human interference in the behavior of the sheep.

To analyze the pain-related behavior in sheep, the research was divided into the following phases: 1) elaboration of an ethogram to characterize the behavior of the animals before and after the painful procedure (S1 Table); 2) content validation of the normal and pain behaviors based on previous studies, the pilot study, and the ethogram [8,28,31,32,52–66] (S2 Table); 3) production of a pre-refinement scale (S3 Table - scale 1), used to evaluate the videos, which were reevaluated after 30 days, by four observers who were blinded regarding the moments; 4) statistical analysis of the pre-refinement scale (S3 Table) evaluated by the observers according to the criteria in Table 1; 5) refinement criteria applied the scale (S4 Table), based on the statistical analysis of Table 1; 6) validation of the final scale (scale 2) after refinement (Table 3); 7) comparison of the final scale with the facial scale and the three unidimensional scales (Fig 2).

Fig 2. Flowchart with the stages of elaboration, refinement, and validation of the USAPS. Statistical tests (table 1) used in refinement and final validation of the scale: 1) content validation (only in refinement); 2) multiple association; 3) intra-observer reliability; 4) inter-observer reliability; 5) criterion validity; 6) construct validity; 7) internal consistency; 8) sensitivity and specificity; 9) determination of the analgesic rescue point (only in refinement).

Ethogram

To elaborate the ethogram, the presential researcher evaluated, twice, 20-minute videos of each of the four moments (48 animals x 4 = 192 videos), for a total of 64 hours of filming, through recordings continuously evaluated using the focal animal method [76]. These assessments aimed to: 1) recognize the different behaviors and 2) measure the length of time (in seconds) of each behavior presented in S1 Table and its respective percentage of time in relation to the total recording time of 20 minutes. Next the videos were edited with the inclusion of the predominant behaviors for the period of about three minutes at each moment. The edited videos were evaluated by four observers for the scale validation process.

Pain scale

The videos were made available on a virtual platform to the observers, who evaluated the four moments of each animal, in a blind and randomized manner regarding the moments. At the end of the observation of each video, the observers, based on their clinical experience, answered whether or not they would administer rescue analgesia (0 = no and 1 = yes to do rescue analgesia). These data were used to determine the cut-off point related to the need for analgesic intervention. Next, pain scores were determined using three unidimensional scales (NS, SDS, and VAS). The NS ranges from "0" to "10", where "0" represents no pain and "10" the worst possible or imaginable pain; the SDS ranges from 1 - no pain, 2 - mild pain, 3 - moderate pain, and 4 - severe pain; and the VAS is based on a straight line 100 mm long, where “0” represents the animal without pain and “100” the worst possible pain [34,42,43].

As some sub-items in S3 Table used to evaluate the videos, contained a lot of grouped descriptors (example: “locomotion”), they were subdivided for individual assessment of the importance of each one. The scale assessed by the four evaluators was based on a scale composed of seven items - five with three sub-items and two with four sub-items. In addition, some of the sub-items had several sub-divisions, which totaled 33 behavioral variables. Finally, the facial pain scale in sheep [69], was applied, through the photographic record captured by the presential observer at the end of the 20-minute recording of each moment.

For the refinement and validation of the acute pain scale and facial scale, the gold standard observer was selected through that which presented greater intra-observer reliability and greater consistency when indicating less rescue analgesics at the moment without pain (M1) and more rescue analgesics at the moment with pain (M2). For refinement, all items and sub-items of the pre-refinement scale (S3 Table) were evaluated according to the statistical analysis of Table 1, and refinement criteria described in S4 Table.

Statistical analysis

Statistical analyzes were performed using R software in the RStudio integrated development environment [77]. For all analyzes, an α of 5% was considered. The sample size was estimated in 13 with a 0.90 test power, α = 0.0001; for the chi-square test (http://biomath.info/power/chsq.htm). For this estimate, the effect size was calculated, applying the percentage of need for rescue analgesia at M1 (without pain; 8%) and M2 (in pain; 92%) according to the data of the 2nd assessment of the gold standard evaluator (Table 13).

For the analysis of the ethogram, the time spent in seconds and the percentages of duration of each behavioral category between the four moments were compared using the Friedman test [29,47]. Table 1 presents the guidelines for the refinement and statistical validation process of the proposed scale to quantify acute pain in, with a description of the types of analysis performed and the respective statistical tests.

Pre-refinement scale (S3 Table) was submitted to refinement according to Table 1. To refine the validated final scale, the subsequent inclusion and exclusion criteria of items and sub-items were followed according to 10 statistical tests, based on data from the second phase of the gold standard observer assessment (S4 Table): 1) ethogram (highest score of behavior by the Friedman test at M2 *vs* the other moments); 2) content validation; 3) at least 15% frequency of occurrence of items/sub-items at M2; 4) multiple association (principal component analysis); 5) intra-observer reliability; 6) inter-observer reliability; 7) construct validity (Friedman's tests - highest score of item/sub-item at M2 *vs* the other three moments); 8) item-total correlation; 9) internal consistency; 10) specificity and sensitivity. The itens anda sub-items were submitted to these analyses according to the criteria of Table 1. The variables that met the criteria stipulated in more than 50% of these statistical tests were accepted. Data supporting the results are available in supplementary material.

Table 1. Guidelines for statistical refinement ([R]) and validation ([V]) of the Unesp-Botucatu sheep acute pain scale (USAPS).

Type of analysis	Description	Statistical test
**Content validation[R]**	The following steps were performed: 1) a list of pain-related behaviors reported in the literature and 2) behaviors observed in the ethogram were scored by 3) a committee composed of three experienced veterinarians used to assess pain in ruminants who analyzed each sub-item within each item of the scale into relevant (+1), do not know (0), or irrelevant (-1)	All the values of each sub-item (-1, 0, or 1) were added and the total was divided by the number of observers. Items with a total score > 0.5 were included in the scale [78].
**Intra-observer reliability[RV]**	**Repeatability** - the level of agreement of each observer with themself was estimated by comparing the two phases of assessment, using the scores of each item, the total sum of the USAPS, numerical (NS), simple descriptive (SDS), and visual analogue scales (VAS) as well as the need for analgesic rescue.	For the scores of the items of the USAPS and the NS and SDS, and the need for analgesic rescue, the **weighted kappa coefficient** (***k_w***) was used; the disagreements were weighted according to their distance to the square of perfect agreement. The 95% confidence interval (CI) k_w ("cohen.kappa" function of the "psych" package) was estimated. For the VAS, the **intraclass correlation coefficient (ICC)** type "**agreement**" was used and its 95% CI ("icc" function of the "irr" package) [79–81]. For the sum of the **behavioral scale**, the consistency type ICC and its 95% CI were used. Interpretation of k_w and ICC: very good 0.81 - 1.0; good: 0.61 - 0.80; moderate: 0.41 - 0.60; reasonable: 0.21 - 0.4; poor < 0.2. The k_w and ICC> 0.50 was used as a criterion to refine the scale [47,82,84].
**Inter-observer reliability[RV]**	**1) Reproducibility** – the level of agreement between the gold standard observer and the other three observers in the 2nd evaluation phase was estimated, using the scores for each item; the total sum of the behavioral scale, NS, SDS, VAS, and the need for rescue analgesic.	
	**2) Agreement matrix** - a matrix was generated to assess the agreement of the total score of the USAPS in the 2nd evaluation phase of each observer *versus* all other observers.	
**Criterion validity[RV]**	**1) Concurrent criterion validity (relationship with gold standard method)**: the correlation of the sum of the behavioral scale was estimated with the NS, SDS, VAS, and facial expression scale of all grouped moments scored by the gold standard observer in the 2nd evaluation phase.	Spearman rank correlation coefficient (rs; "rcorr" function of the "Hmisc" package). Interpretation of the degree of correlation rs (p<0.05): 0 - 0.35: low correlation; 0.35 - 0.7: average correlation; 0.7 - 1.0: high correlation [47].
	**2) Concurrent criterion validity:** agreement between the gold standard and the other evaluators (reproducibility)	Please see above the item **Inter-observer reliability: 1) Reproducibility.**
	**3) Predictive criterion validity** was assessed by the number of sheep that should receive rescue analgesia according to the Youden Index (described below) in the moment of greatest pain (M2).	Descriptive analysis.
**Construct validity[RV]**	**Responsiveness** – the behavior from the ethogram observed by the presential researcher at the four moments of evaluation was compared; the scores of each item and the total score of the behavioral scale, NS, SDS, VAS, and the need for rescue analgesic over time (M1 *vs* M2 *vs* M3 *vs* M4) were compared according to the 2nd phase of the gold standard observer assessment.<br>Interpretation: differences in scores are expected to occur as follows: M2>M4≥M3>M1.<br>Construct validity was determined using the **three hypothesis test method**: 1) if the scale really measures pain, the score after surgery (M2) should be higher than the preoperative score (M1 *vs* M2); 2) the score should decrease after analgesia (M2 *vs* M3); 3) and over time (M2 *vs* M4).	For dichotomous variables (**need for rescue analgesic**) **logistic regression analysis** ("glm" function of the "stats" package) was applied using the **Tukey test** ("lsmeans" function of the "lsmeans" package) as a post hoc test. The **normality** of each variable at each moment was assessed by graphs of boxes and histograms ("boxplot" and "histogram" functions of the "graphics" and "lattice" packages, respectively). From these graphs, the pain scale items, NS, SDS, and VAS were considered nonparametric. Thus, we used the **Friedman test** (function "friedman.test" of the package "stats") in which the p-value was corrected with the **Bonferroni** procedure (function "pairwiseSignTest" of the package "rcompanion") [29,47]. For the **total score of the scale**, a mixed linear model ("lmer" function of the "lme4" package) was used, including the **moments** (M1, M2, M3 and M4) **and breeds** as fixed effects and the random effect of individuals, in which the p-value was also corrected by the **Bonferroni** procedure. The differences between the

		moments were identified by different letters (“cldList” function of the “rcompanion” package).
**Multiple association**RV	The multiple association of the items with each other was analyzed at all moments grouped on the behavioral scale in the 2nd phase of the gold standard observer assessment, through the analysis of main components, to define the number of dimensions-determined by different variables that establish the scale extension.	**Principal component analysis** (“princomp” and “get_pca_var” functions from the “stats” and “factoextra” packages respectively). According to the **Kaiser** criterion [83], representative dimensions of the components were selected with eigenvalue > 1 and variance> 20 and each item on the scale with a load value ≥ 0.50 or ≤ - 0.50.
**Item-total correlation**RV	Correlation coefficient of item score with total score. To analyze homogeneity, the relevance of each item on the scale, and to investigate inflationary items, the correlation of each item with the sum of all other items on the scale, by excluding the evaluated item, was estimated). Analysis was performed for all moments grouped (MG) in the 2nd phase of the gold standard observer assessment.	**Spearman rank correlation coefficient** (r_s; “rcorr” function of the “Hmisc” package). Interpretation of correlation r_s: suitable values 0.3 - 0.7 [84]. Itens were accepted if >0,3.
**Internal consistency**RV	The consistency (interrelation) of the scores for each item on the scale was estimated and the process was performed again to exclude each item. The analysis was performed for each of the 4 moments and for all of them grouped (MG) in the 2nd phase of the gold standard observer assessment.	**Cronbach's alpha coefficient** (α; "cronbach" function of the "psy" package) [84] Interpretation: 0.60-0.64, minimally acceptable; 0.65-0.69 acceptable; 0.70-0.74 good; 0.75-0.80 very good; and> 0.80 excellent [84–86].
**Specificity**RV	The scores of the behavioral scale in the 2nd phase of assessment of the gold standard observer at M1 were transformed into dichotomous (level “0” - absence of pain expression behavior for a given item; levels “1” and “2” - presence of pain expression behavior) and applied to the respective equation.	$Sp_{M1} = \frac{TN}{TN + FP}$ ***Sp*** = specificity. **TN = true negative** (scores that represented painless behaviors - "0" - at the time when the animals were expected to have no pain, since it was before surgery - M1). **FP = false positive** (scores that represented pain expression behaviors - 1 or 2 - at the time when the animals were expected to have no pain, since it was before surgery – M1). Interpretation: excellent 95 - 100%; good 85 - 94.9%; moderate 70 - 84.9%; not sensitive <70%. Only items ≥ 70% were included pain [84].
**Sensitivity**RV	The scores of the behavioral scale in the 2nd phase of assessment of the gold standard observer at M2 were transformed into dichotomous (as described for specificity) and applied to the respective equation.	$S_{M2} = \frac{TP}{TP + FN}$ ***S*** = sensitivity. **TP = true positive** (scores that represented pain expression behaviors - 1 or 2 - at the time the animals were expected to have pain, since it was after surgery– M2). **FN = false negative** (scores representing painless behaviors - 0 - at the time the animals were expected to have pain, since it was after surgery – M2). Interpretation: excellent 95 - 100%; good 85 - 94.9%; moderate 70 - 84.9%; not sensitive <70%. Only items ≥ 70% were included [40,84].
**Distribution of scores**V	A table was constructed with the distribution of the frequency of the presence of the scores 0, 1, and 2 of each item at each moment and in the MG scored in the 2nd phase of the gold standard observer assessment.	Descriptive analysis.
**Rescue analgesic point** V	The **need for analgesia** according to the clinical experience after the analysis of the videos was used as the true value and the **sum of the behavior scale** as a predictive value to build a **receiver operating characteristic curve (ROC)**. Then, the cut-off point for analgesic rescue was determined based on the **Youden index** and its **diagnostic**	$YI = (S + Sp) - 1$ ***YI* = Youden Index** ; *S* = sensitivity; *Sp* = specificity. Analysis of the receiver operating characteristic curve (ROC; “roc” function of the “pROC” package) and the area under the curve (AUC): graphical representation of the relationship between the “TP” (S) and the “FP” (1-*Sp*). *YI* is the point of

	**uncertainty zone** using all moments of pain assessment on the behaviour scale scored by each and all observers in the 2nd evaluation phase.<br>**Cut-off point** was represented by the Youden index using all moments of pain assessment on the behaviour scale, NS, SDS and VAS scored by all observers in the 2nd evaluation phase.<br>The **area under the curve (AUC)** was calculated, which indicates the discriminatory capacity of the test.<br>The **frequency and percentage of animals scored in the diagnostic uncertainty zone** of the cut-off point of all evaluators for the behaviour scale, NS, SDS, VAS were calculated using descriptive statistical analysis.	greatest sensitivity and specificity simultaneously, determined by the ROC curve [87]. **Interpretation:** AUC ≥ 0.95 - high discriminatory capacity of the scale [88].<br>The **diagnostic uncertainty zone** was determined by two methods, calculating: 1st) the 95% confidence interval (CI) replicating the original ROC curve 1,001 times by the bootstrap method (“ci.coords” and “ci.auc” functions of “pROC” package); 2) the interval between the sensitivity and specificity values of 0.90. The highest interval of one these two methods of all the evaluators were considered the diagnostic uncertainty zone, which indicates the diagnostic **accuracy** [89–92].
**Determination of pain intensity** [V]	Scores were classified by intensity: **low, intermediate or high,** in 2nd evaluation of all evaluators at the time of greatest pain (M2).	Non-hierarchical cluster analysis was employed, applying the “centroid” by using the “maximum” method (“dist” and “hclust” functions in the “stats” package) [93] followed by the Kruskal-Wallis test (“kruskal” function in the “agricolae” package) ”) to assess the difference between the groups formed by the cluster analysis.

Scales: numerical (NS), simple descriptive (SDS), visual analogue (VAS). Statistical analyzes were performed using R software in the RStudio integrated development environment [77]. For all analyzes, α of 5% was considered, MG - data of grouped moments (M1 + M2 + M3 + M4).

Results

A minimum sample size of 5 sheep [67] was estimated.

Behaviour data

With respect to the ethogram, from the description of the behaviors (S1 Table) and the time and percentages of each observed behavior (Table 2), comparing the moment of greatest pain (M2) with that of the supposed absence of pain (M1), the following differences were observed: at M2 there was a decrease in times of “normal interaction”, “ normal locomotion”, and “head above the withers” and an increase in times of “reduced and absent locomotion” and “arch back”. After the analgesic rescue (M3), compared to M2, the times of “eat” and normal “interaction” ” increased, and “normal locomotion” and “head below the withers” decreased. The act of “eat” increased at M3, in relation to M2, because the animals were fasting at M1 and therefore it was not possible to compare M2 vs M1 for this variable.

Pain scale data

According to the inclusion/exclusion criteria cited for the refinement (S4 Table), the following sub-items were excluded: in the item “locomotion”, “walks backwards” and “walks in a circle”; in the item "posture", "kicks and stamps limbs on the ground" and "extends one or more limbs; in the item “miscellaneous behavior”, “body tremors” and “crawls in ventral recumbence, without getting up” were excluded. The two sub-items that remained in the “miscellaneous” item replaced those excluded from the “posture” item. The “miscellaneous” item continued with four sub-items and was renamed “posture”.

Next, the final version of the USAPS, containing six items (five with three sub-items and one with four sub-items) was validated (Table 3). The total score was based on the sum of each item, ranging from zero (without pain) to 12 (maximum pain).

Table 2. Median and range of the percentage of duration of behaviors of 48 sheep before and after laparoscopy.

Moments	M1		M2		M3		M4	
**Behavior category**	**Median**	Range	**Median**	Range	**Median**	Range	**Median**	Range
**Eat**	**0^{c}**	0 – 0	**11^{b}**	0 – 82	**31.25^{a}**	0 – 86	**34^{ab}**	0 – 71
**Ruminate**	**0^{b}**	0 – 24	**0^{ab}**	0 – 41	**4.04^{a}**	0 – 20	**4^{a}**	0 – 44
Drink	0	0 – 7	0	0 – 26	0	0 – 16	0	0 – 2
Urinate	0	0 – 9	0	0 – 12	0	0 – 21	0	0 – 5
Defecate	0	0 – 0	0	0 – 19	0	0 – 0	0	0 – 0
**Normal interaction**	**44^{a}**	0 – 100	**0^{b}**	0 – 91	**7.15^{a}**	0 – 95	**61^{a}**	0 – 97
Reduced interaction	0^{a}	0 – 97	8^{a}	0 – 94	0^{a}	0 – 100	0^{b}	0 – 97
Absent interaction	0	0 – 100	0	0 – 100	0	0 – 30	0	0 – 88
**Normal locomotion**	**23^{a}**	0 – 57	**0^{b}**	0 – 68	**0^{c}**	0 – 47	**14^{a}**	0 – 50
Reduced/altered locomotion	0^{b}	0 – 21	0^{a}	0 – 25	0^{b}	0 – 40	0^{a}	0 – 47
Absent/abnormal locomotion	0^{b}	0 – 70	2^{a}	0 – 100	0^{ab}	0 – 60	0^{b}	0 – 30
**Head above the withers**	**11^{a}**	0 – 80	**0^{b}**	0 – 50	**0^{b}**	0 – 67	**21^{a}**	0 – 86
Head at the height of the withers	0	0 – 68	0	0 – 78	0	0 – 87	0	0 – 81
Head below the withers	10^{a}	0 – 70	13^{a}	0 – 81	6.46^{b}	0 – 60	5^{b}	0 – 25
Standing still in normal posture	61^{b}	33 – 89	67^{ab}	0 – 94	73.87^{a}	0 – 100	74^{ab}	10 – 92
Standing in altered posture	5^{a}	0 – 31	6^{a}	0 – 37	1.04^{ab}	0 – 60	0^{b}	0 – 8
Kick and stamp the limbs on the ground	0^{b}	0 – 0	0^{ab}	0 – 5	0^{b}	0 – 0	0^{a}	0 – 14
Lying down with extension of the head and neck and/or limb(s)	0^{ab}	0 – 31	0^{a}	0 – 38	0^{ab}	0 – 60	0^{b}	0 – 11
Lying down	0	0 – 31	0	0 – 90	0	0 – 35	0	0 – 44
Lying down with head turned back	0	0 – 0	0	0 – 0	0	0 – 0	0	0 – 0
Lying with head supported on or close to the ground	0	0 – 28	0	0 – 100	0	0 – 32	0	0 – 0
Look at affected area	0	0 – 0	0	0 – 0	0	0 – 0	0	0 – 0
Lick the affected area	0	0 – 0	0	0 – 0	0	0 – 0	0	0 – 0
Quick and repeated tail movements	0	0 – 40	0	0 – 0	0	0 – 0	0	0 – 0
Keep the tail straight	0	0 – 22	0	0 – 6	0	0 – 0	0	0 – 50
**Arch the back**	**0^{b}**	0 – 0	**0^{a}**	0 – 34	**0^{ab}**	0 – 13	**0^{ab}**	0 – 7
Body tremors	0	0 – 0	0	0 – 11	0	0 – 0	0	0 – 0

The percentages of each behavioral category were calculated based on the total time of each evaluation moment (20 mins). Different letters express significant differences between moments (values in bold express differences at M2 compared to M1, or M3, specifically for “eating”) with a>b>c, according to the Friedman test ($p < 0.05$) [29,47]. M1: preoperative; M2 - postoperative, before analgesic rescue; M3 - postoperative, after analgesic rescue; M4 - 24h after surgery.

Table 3. Final validated Unesp-Botucatu sheep acute pain scale (USAPS).

**Item**	**Sub-item** (descriptors)	**Score**	**Links to videos**
**Interaction**	Active, attentive to the environment, interacts and/or follows other animals.	0	https://www.youtube.com/watch?v=4fOJWD-uNbg&t=9s
	Apathetic: may remain close to other animals, but interacts little.	1	https://www.youtube.com/watch?v=EEyMC_VIMpk
	Very apathetic: isolated or not interacting with other animals, not interested in the environment.	2	https://www.youtube.com/watch?v=5NsthhKoEP4
**Activity**	Moves normally	0	https://www.youtube.com/watch?v=dDx9FesiA2M
	Restless, moves more than normal or lies down and gets up frequently.	1	https://www.youtube.com/watch?v=3MjccV2yV74
	Moves less frequently or only when stimulated or does not move.	2	https://www.youtube.com/watch?v=EvLDBJo93jo
**Locomotion**	Moves about freely, without altered locomotion; when stopped, the pelvic limbs are parallel to the thoracic limbs.	0	https://www.youtube.com/watch?v=W0Hw2Ibqbyk
	Moves about with restriction and/or short steps and/or pauses and/or lameness; when stopped, the thoracic or pelvic limbs may be more open and further back than normal.	1	https://www.youtube.com/watch?v=i8FxBj-yQhw
	Difficulty and/or reluctanct to get up and/or not moving and/or walking abnormally and/or limping; leans against a surface.	2	https://www.youtube.com/watch?v=dPdT9VMJTi0
**Head position**	Above the withers or eating.	0	https://www.youtube.com/watch?v=W8mi15I1dr8
	At the height of the withers.	1	https://www.youtube.com/watch?v=8xSUmoXaiZY
	Below the withers.	2	https://www.youtube.com/watch?v=YRxpWSTsqpw
**Appetite**	Normorexia and/or rumination present.	0	https://www.youtube.com/watch?v=no1VeiFglUE
	Hyporexia.	1	https://www.youtube.com/watch?v=aIEY1UkqQ-k
	Anorexia.	2	https://www.youtube.com/watch?v=YV40N-OHuNI
**Posture**	A. Arched back.		https://www.youtube.com/watch?v=gloa-38gTW8
	B. Extends the head and neck.		https://www.youtube.com/watch?v=rNh_aFePKAE
	C. Lying down with head resting on the ground or close to the ground		https://www.youtube.com/watch?v=LT6BJzhZO9E
	D. Moves the tail quickly (except when breastfeeding) and repeatedly and/or keeps the tail straight (except to defecate/urinate)		https://www.youtube.com/watch?v=91RbQMsa8Mg
	• Absence of these behaviors	0	
	• Presence of one of the related behaviors	1	
	• Presence of two or more of the related behaviors	2	

Intra-observer reliability

Repeatability ranged from reasonable to good for each item on the USAPS (with the exception of appetite for evaluator 3 which was poor) and from good to very good for their total score at all moments assessed (Table 4).

Table 4. Repeatability of USAPS, unidimensional scales and rescue analgesia indication in sheep.

**Evaluator**	**1**			**2** (gold)			**3**			**4**		
**Items**	***k_w***	Min	Max	***k_w***	Min	Max	***k_w***	Min	Max	***k_w***	Min	Max
Interaction	**0.64**	0.55	0.74	**0.66**	0.56	0.77	**0.52**	0.41	0.64	**0.65**	0.56	0.74
Activity	**0.54**	0.42	0.66	**0.65**	0.54	0.76	0.46	0.34	0.58	0.47	0.35	0.60
Locomotion	**0.71**	0.63	0.80	**0.66**	0.55	0.77	0.48	0.36	0.61	**0.61**	0.52	0.71
Head position	**0.67**	0.56	0.77	**0.71**	0.62	0.80	0.48	0.35	0.61	**0.59**	0.48	0.69
Appetite	**0.55**	0.42	0.69	**0.61**	0.48	0.73	**0.56**	0.43	0.69	0.15	0.02	0.28
Posture	0.42	0.27	0.57	**0.61**	0.48	0.73	0.45	0.28	0.61	0.32	0.23	0.42
**RA**	**0.67**	0.56	0.77	**0.75**	0.65	0.85	**0.53**	0.41	0.65	**0.53**	0.40	0.65
**NS**	**0.80**	0.74	0.86	**0.85**	0.80	0.91	**0.58**	0.48	0.68	**0.72**	0.64	0.79
**SDS**	**0.78**	0.71	0.85	**0.77**	0.69	0.84	**0.61**	0.50	0.71	**0.67**	0.59	0.76
**Scales**	**ICC**	CI		**ICC**	CI		**ICC**	CI		**ICC**	CI	
**USAPS**	**0.77**	0.70	0.82	**0.83**	0.78	0.87	**0.65**	0.57	0.73	**0.71**	0.64	0.78
**VAS**	**0.80**	0.74	0.85	**0.81**	0.76	0.86	**0.56**	0.44	0.64	**0.71**	0.63	0.77

Scales: USAPS - Unesp-Botucatu sheep acute composite pain scale; NS – numerical; SDS - simple descriptive; VAS - visual analogue; RA - Rescue analgesia. Each item of the USAPS, the NS and SDS was calculated with kappa coefficient (*kw*); Sum of the USAPS and the VAS was calculated using intraclass correlation coefficient (ICC consistency); CI - Confidence interval. Interpretation of the degree of reliability k_w or ICC (consistency): very good: 0.81 - 1.0; good: 0.61 - 0.80; moderate: 0.41 - 0.60; reasonable: 0.21 - 0.4; poor <0.2 Bold type corresponds to acceptable values > 50 [47,82,84].

Inter-observer reliability

Evaluator 2 was selected as the gold standard. Reproducibility between the gold standard and the other three observers for each item on the USAPS was moderate, with the exception of one evaluator that ranged from poor to moderate (Table 6).

Table 5. Reproducibility of USAPS, unidimensional scales and rescue analgesia indication between the gold standard and the other observers.

	Evaluator 2 (gold-standard) *vs*								
**Evaluators**	**1**			**3**			**4**		
**Items**	***k_w***	Min	Max	***k_w***	Min	Max	***k_w***	Min	Max
Interaction	**0.52**	0.42	0.62	**0.60**	0.49	0.70	0.45	0.35	0.55
Activity	**0.58**	0.47	0.69	**0.55**	0.43	0.67	**0.50**	0.35	0.55
Locomotion	**0.56**	0.46	0.66	0.48	0.35	0.60	0.49	0.37	0.60
Head position	**0.54**	0.42	0.65	0.48	0.36	0.61	0.43	0.31	0.54
Appetite	**0.57**	0.44	0.69	**0.63**	0.51	0.75	0.18	0.03	0.33
Posture	0.47	0.33	0.61	**0.50**	0.36	0.64	0.31	0.19	0.42
**RA**	**0.53**	0.41	0.64	**0.63**	0.52	0.74	0.46	0.35	0.57
**NS**	0.43	0.36	0.50	**0.66**	0.59	0.73	0.49	0.41	0.56
**SDS**	**0.65**	0.57	0.74	**0.65**	0.56	0.74	**0.63**	0.55	0.72
**Scales**	**ICC**	CI		**ICC**	CI		**ICC**	CI	
**USAPS**	**0.72**	0.65	0.78	**0.76**	0.70	0.82	**0.63**	0.53	0.70
**VAS**	0.30	0.16	0.42	**0.59**	0.49	0.68	0.44	0.32	0.55

Scales: USAPS - Unesp-Botucatu sheep acute composite pain scale; NS – numerical; SDS - simple descriptive; VAS - visual analogue; RA - Rescue analgesia. Each item of the USAPS, the NS and SDS was calculated with kappa coefficient (*kw*); Sum of the USAPS and the VAS was calculated using intraclass correlation coefficient (ICC consistency); CI - Confidence interval. Interpretation of the degree of reliability k_w or ICC (consistency): very good: 0.81 - 1.0; good: 0.61 - 0.80; moderate: 0.41 - 0.60; reasonable: 0.21 - 0.4; poor <0.2. Bold type corresponds to acceptable values > 50 [47,82,84].

Criterion validity

Concurrent criterion validity

Reproducibility of the total score of USAPS according to matrix correlation was good among all observers (Table 6).

Table 6. Reproducibility matrix of the USAPS.

**Evaluator**	**1**		**2** (gold)		**3**	
**ICC**	**ICC**	**CI**	**ICC**	**CI**	**ICC**	**CI**
**1**						
**2**	**0.72**	0.65 - 0.78				
**3**	**0.71**	0.64 - 0.78	**0.76**	0.70 - 0.82		
**4**	**0.74**	0.67 - 0.80	**0.63**	0.53 - 0.70	**0.63**	0.54 - 0.71

USAPS - Unesp-Botucatu sheep acute composite pain scale. Intra-class correlation coefficient (ICC consistency). Interpretation of the degree of reliability (ICC consistency): very good: 0.81 - 1.0; good: 0.61 - 0.80; moderate: 0.41 - 0.60; reasonable: 0.21 - 0.4; poor <0.2. Bold type corresponds to acceptable values> 50 [47,82,84].

The concurrent criterion validation test registered a high correlation between pain scale scores and those of the NS (r = 0.87), SDS (r = 0.86), and VAS (r = 0.81), and

moderate correlation with the facial scale (r = 0.59) for all moments (MG) evaluated by the four evaluators (Table 7; Fig 3).

Table 7. Correlation between the USAPS and the numerical, simple descriptive, visual analogue, and facial expression scales.

Scales / USAPS	MG
**NS**	**0.87**
**SDS**	**0.86**
**VAS**	**0.81**
Facial expression scale	0.59

Scales: USAPS - Unesp-Botucatu sheep acute composite pain scale; NS – numerical; SDS - simple descriptive; VAS - visual analogue; MG - data of grouped moments (M1 + M2 + M3 + M4). Interpretation of Spearman's correlation coefficient: good ≥ 0.75; moderate: 0.5-0.74; poor < 0.5 [84]. Bold values correlation ≥ 75.

Fig 3. Correlation between the scores of the USAPS (Unesp-Botucatu sheep acute composite pain scale) and those of the numerical, simple descriptive, visual analogue and facial expression scales for all moments.

Predictive criterion validity

Considering the predictive criterion validity, grounded by the Youden index, between 83 and 98% of sheep (90% considering all the grouped evaluators) would receive rescue analgesia in the moment of most intense pain (M2) (Table 8). Unnecessary analgesia would be indicated in 8 to 23% of sheep at M1, based on the score given by each evaluator and 15% considering all the grouped evaluators. This result demonstrated that the scale was sensitive in distinguishing pain, otherwise specific in distinguishing sheep not suffering pain.

There was no difference between indication of rescue analgesia according to clinical experience and according to the Youden index of the USAPS (p = 0.74 - Fisher's Exact Test).

Table 8. Percentage of sheep for which was indicated rescue analgesia according to clinical experience and according to the Youden index of the USAPS.

**Evaluator**	**1**		**2** (gold)		**3**		**4**		**All**	
**RA**	**Exp**	***YI***	**Exp**	***YI***	**Exp**	***YI***	**Exp**	***YI***	**Exp**	***YI***
**M1**	13	13	8	8	23	15	19	25	**16**	**15**
**M2**	90	90	83	83	83	90	98	98	**89**	**90**

Calculation based on 48 sheep for each observer and 192 sheep for all evaluators. RA – indication of rescue analgesia according to clinical experience scored at the end of each video analysis (Exp) and according to the Youden index of the USAPS (score ≥ 4). USAPS - UNESP-Botucatu sheep composite acute pain scale. Gold - gold standard observer; M1 - preoperative; M2 - postoperative, before rescue analgesia; Youden index ≥ 4 is representative of the cut-off point for indication of rescue analgesia (see Table 12 for results of Youden index).

Construct validity (responsiveness)

The scores for all items and the sum of the scale were significantly higher at M2 than at M1, M3, and M4, demonstrating their responsiveness (construct validity). The differences between the total scores of the proposed scale, the NS, SDS, and VAS were as follows: M2>M3>M4>M1 (Table 9; Fig 4). In the statistical model, the fixed effect of breeds did not have any significant influence on the total score of the final scale.

Table 9. Responsiveness of the USAPS, rescue analgesia and unidimensional pain scales, between the four perioperative moments.

Moments	M1		M2		M3		M4	
**Items**	Median	Amplit.	Median	Amplit.	Median	Amplit.	Median	Amplit.
Interaction	0^{bc}	0 - 2	1^{a}	0 - 2	0^{b}	0 - 2	0^{c}	0 - 1
Activity	0^{bc}	0 - 2	2^{a}	0 - 2	0^{b}	0 - 2	0^{c}	0 - 2
Locomotion	0^{c}	0 - 2	1^{a}	0 - 2	0.5^{b}	0 - 2	0^{c}	0 - 1
Head position	0^{bc}	0 - 2	1^{a}	0 - 2	0^{b}	0 - 2	0^{c}	0 - 1
Appetite	0^{bc}	0 - 0	0.5^{a}	0 - 2	0^{b}	0 - 2	0^{b}	0 - 2
Posture	0^{bc}	0 - 1	1^{a}	0 - 2	0^{b}	0 - 2	0^{b}	0 - 1
**USAPS**	2^{c}	0 - 8	7^{a}	0 - 12	2.5^{b}	0 - 10	0^{c}	0 - 7
**RA**	0^{c}	0-1	1^{a}	0-1	0^{b}	0-1	0^{c}	0-1
**NS**	1^{c}	1-4	5^{a}	1-8	2^{b}	1-5	1^{c}	1-5
**SDS**	1^{c}	1-3	3^{a}	1-3	2^{b}	1-3	1^{c}	1-3
**VAS**	8.5^{bc}	2-38	43^{a}	7-75	15^{b}	4-53	7^{c}	3-47

Each item and total score of the USAPS (0-12): Unesp-Botucatu sheep acute composite pain scale; RA - Rescue analgesia (0 no; 1 yes); NS (1-10), SDS (1-4) and VAS (0-100). Different letters express significant differences between moments where a> b> c, according to the Friedman test ($p < 0.05$) [29,47]. M1: preoperative; M2: postoperative, before rescue analgesia; M3: postoperative, after rescue analgesia; M4: 24h postoperative.

Fig 4. Box-plot of the scores (median/amplitude) of the USAPS, comparing the four perioperative moments.

Principal component analysis

The multiple association of the items of the scale with each other, at all moments, evaluated through principal component analysis selected the main component 1, corresponding to one representative dimension, the mathematical reason why the scale was considered unidimensional (Table 10; Fig 5).

**Items**	**Dimension 1**	**Dimension 2**
	Load value	Load value
Interaction	**0.82**	-0.06
Activity	**0.80**	-0.05
Locomotion	**0.85**	0.05
Head position	**0.73**	0.17
Appetite	**0.59**	**-0.70**
Posture	**0.68**	**0.50**
**Eigenvalue**	**3.39**	0.78
**Variance**	**56.39**	12.91

Table 10. Load values, eigenvalues and variance of the USAPS items with each other after principal components analysis.

USAPS – Unesp-Botucatu sheep acute pain scale. The structure was determined considering items with a load value ≥ 0.50 or ≤ -0.50, with representative dimension (eigenvalue > 1 and variance > 20%). The load values in bold indicate the variables that contribute to each dimension and the respective accepted eigenvalue and variance [83].

Fig 5. Biplot for the principal components analysis with the items of the USAPS.

USAPS – Unesp-Botucatu sheep acute pain scale. Confidence ellipses were built according to the moments of pain and animal assessment. The data were obtained from the analysis of the gold standard observer at M1 - preoperative; M2 - postoperative, before analgesic rescue; M3 - postoperative, after analgesic rescue; M4 - 24h after surgery. Ellipses were constructed according to the moments of pain assessment (M1 - green, M2 - red, M3 – blue, and M4 - yellow). The ellipse referring to the time when sheep were in severe pain (M2) was positioned at the right end of the graph, while on the opposite side were the times when the sheep were probably not in pain (M1 and M4). The moment of moderate pain (M3) is positioned in the middle. All items on the scale are influenced by moments of pain (M2 and M3) since their vectors are positioned in the direction of these ellipses.

Item-total correlation

Correlation coefficient of item score with total score (item-total score) ranged from 0.43 to 0.72 and, therefore, all items were accepted (Table 11).

Internal consistency

The Cronbach's α coefficient was 0.84 for all moments grouped, which indicates that the instrument presents excellent internal consistency and reinforces the possibility of using the full scale score to interpret the results obtained. Internal consistency was very good when locomotion (0.78) and interaction (0.79) were excluded and excellent when all other individual items were excluded, showing that all items contributed similarly and significantly to the total score (Table 11).

Specificity and sensitivity

All items on the USAPS showed moderate to excellent specificity. The items "interaction", "activity" and "locomotion" presented moderate or good sensitivity. "Head position", "appetite" and "posture" were not sensitive (Table 11).

Table 11. Item-total correlation, internal consistency, specificity and sensitivity of the USAPS.

Items	Item-total (Spearman)	Internal consistency (Cronbach's α)	Specificity (%)	Sensitivity (%)
**Full scale**		**0.84**		
	**Excluding each item below**			
**Interaction**	**0.72**	**0.79**	**87**	**81**
**Activity**	**0.69**	**0.81**	**83**	**85**
**Locomotion**	**0.71**	**0.78**	**87**	**83**
**Head position**	**0.54**	**0.82**	**71**	69
**Appetite**	**0.43**	**0.84**	**100**	50
**Posture**	**0.53**	**0.83**	**79**	67

USAPS: Unesp-Botucatu sheep acute composite pain scale. Interpretation of Spearman's rank correlation coefficient (r_s) - degree of correlation r_s: 0.3 - 0.7: acceptable values in bold [84]. Cronbach's α coefficient was calculated for the total score and excluding each item from the scale. Interpretation of the α coefficient values: 0.60-0.64: minimally acceptable; 0.65-0.69: acceptable; 0.70-0.74: good; 0.75-0.80: very good; > 0.80: excellent [84–86]; bold values > 70. Interpretation of specificity and sensitivity: excellent 95 - 100%; good 85 - 94.9%; moderate 70 - 84.9%; is not specific or sensitive <70%; bold values ≥ 70% [84].

Distribution of scores

The distribution of scores "0", "1" and "2" occurred as expected, according to the degree of pain. The score "0" predominated at moments M1, M3 and M4. Scores "1" and "2" were more frequent in M2 and decreased in M3 (Fig 6). Only the item "activity" of score

"1" was not very representative when all moments were grouped. For calculation purposes, "appetite" was considered arbitrarily normal in M1.

Fig 6. Distribution of the percentage of the presence of the periperative USAPS scores of each item.

M1: preoperative; M2: postoperative, before rescue analgesia; M3: postoperative, after rescue analgesia; M4: 24h postoperative; MG - data of the grouped moments (M1 + M2 + M3 + M4).

ROC Curve, Youden index, cut-off point and diagnostic uncertainty zone of the USAPS

In the analysis of the ROC curve to determine the cut-off point for diagnosing pain and recommending analgesia, the Youden index was ≥ 3 for evaluators 1 and 3, ≥ 4 for evaluators 2 (gold standard) and 4, and again ≥ 4 of 12 for all grouped evaluators. The interval between the sensitivity and specificity values of 0.90 was between 3.3 and 3.7. Resampling confidence interval (Bootstrap) for Youden index was between 2.5 to 3.5. Based on the resampling result, which showed the highest interval between these two methods, the diagnostic uncertainty zone scores ranged from 3 to 4 for all grouped evaluators; therefore, ≤ 2 indicates truly negative pain (sheep without pain) and ≥ 5 indicates truly positive pain (sheep suffering pain). The individual results of the area under the curve were high (AUC > 0.95) for all evaluators, and 0.97 for grouped evaluators (0.96 - 0.98), indicating that the USAPS presents excellent discriminatory capacity (Fig 7).

Fig 7. ROC curve and AUC [above] and ROC curve of two graphs with the diagnostic uncertainty zone for the USAPS [below].

ROC curve (receiver operating characteristic) with a 95% confidence interval (CI) [89] calculated from 1,001 replications and area under the curve (AUC) [above]. Interpretation of AUC ≥ 0.95 - high discriminatory capacity [90]. ROC curve of two graphs, CI of 1,001 replications and sensitivity and specificity> 0.90 applied to estimate the diagnostic uncertainty zone of the cut-off point of each evaluator, according to the Youden index for the Unesp-Botucatu sheep acute composite pain scale (USAPS) [91–94]. Data of all grouped evaluators. The diagnostic uncertainty zone scores ranged from 2.5 (3) to 3.5 (4); therefore, ≤ 2 indicates truly negative pain (sheep without pain) and ≥ 5 indicates truly positive pain (sheep suffering pain). Youden index was ≥ 4, representative of the cut-off point for indication of rescue analgesia.

For the unidimensional scales, the cut-off points for rescue analgesia defined by the ROC curve and the Youden index were ≥ 4 for SN, ≥ 3 for SDS and ≥ 27 for VAS (Table 12).

Table 12. Scores, specificity, sensitivity, and Youden index corresponding to rescue analgesia indiction of the USAPS and unidimensional scales.

Scale	Score	Specificity	Sensitivity	Youden index
**USAPS**	4	0.92	0.92	0.84
**NS**	4	0,97	0,94	0,91
**SDS**	3	1	0,71	0,71
**VAS**	27	0.94	0.96	0,90

Scales: USAPS - Unesp-Botucatu sheep acute composite pain scale; NS – numerical; SDS - simple descriptive; VAS - visual analogue.

The percentage of animals present in the diagnostic uncertainty zone (scores 3 and 4) was low at all times for all evaluators (11%; 9 - 15%). At M2, this percentage for all evaluators grouped was 7% (0 to 13%), which ensures that 93% of the sheep were detected as suffering pain with confidence at this moment (Table 13).

Table 13. Percentage of sheep present in the diagnostic uncertainty zone according to the Youden index of the USAPS.

**Evaluator**	**1**	**2** (gold)	**3**	**4**	**All**
M1	6	8	23	13	**13**
M2	13	10	4	0	**7**
M3	8	21	17	13	**15**
M4	10	8	15	15	**12**
**MG**	9	12	15	10	**11**

USAPS: Unesp-Botucatu sheep acute composite pain scale. Calculation based on 48 sheep for each evaluator and 192 sheep for all evaluators. M1: preoperative; M2: postoperative, before rescue analgesia; M3: postoperative, after rescue analgesia; M4: 24h postoperative; MG - data of grouped moments (M1 + M2 + M3 + M4). The diagnostic uncertainty zone scores ranged from 3 to 4; therefore, ≤ 2 indicates truly negative pain (sheep without pain) and ≥ 5 indicates truly positive pain (sheep suffering pain).

Determination of pain intensity

The total sums of the USAPS scores were divided into three groups (Fig 8) of pain intensity given by median, minimum and maximum and named according to the intensity: mild (1; 0 - 3); moderate (7; 4 - 8) and severe (10; 9 - 12) (Fig 9). From this classification, it was observed that in M2, the moment of greatest pain, 10% of sheep were classified as mild intensity, 47% as moderate intensity and 43% as severe intensity.

Fig 8. Dendogram created by the non-hierarchical cluster analysis based on the total score of the USAPS.

USAPS: Unesp-Botucatu sheep acute composite pain scale. The scores were divided into 3 groups: mild (0 - 3); moderate (4 - 8) and intense (9 - 12) pain intensity.

Fig 9. Box plot for the mild, moderate and severe pain intensity of the scores of the USAPS.

USAPS: Unesp-Botucatu sheep acute composite pain scale. Data were grouped at the time of greatest pain (M2). According to the median, minimum and maximum scores obtained by the non-hierarchical cluster analysis followed by the Kruskal-Wallis test, the scores were divided into 3 groups: mild (1; 0 - 3); moderate (7; 4 - 8) and (10; 9 - 12). Different letters indicate statistical difference by the Kruscal-Wallis test, where a> b> c.

Discussion

The creation of valid species-specific tools to assess pain is a prerequisite for recognizing the phenomenon and determining the need and effectiveness of analgesic treatment. From this perspective, the behavioral pain scale proposed herein can be considered as a reliable, valid instrument with a defined analgesic intervention point, which can be used to assess postoperative abdominal pain in sheep. This instrument demonstrates potential clinical applicability, as it can guarantee that sheep benefit from analgesia when necessary, and potential experimental applicability, for example to perform studies that compare the analgesic efficacy of drugs, as well as translational studies [2,17].

The validation process of an instrument to assess pain is based on the investigation of behaviors and, when possible, of species-specific physiological data

present during pain situations, followed by comparison of these changes with the state of normality [2]. This methodology was followed in the current study; an ethogram was constructed that covered the preoperative period, at which time the animals were supposedly devoid of pain, followed by the postoperative period, at which time the animals probably had severe pain, followed by rescue analgesia for pain reduction and assessment after 24 hours. Thus, the experimental design aimed to test the instrument at different pain intensities. The ethogram, together with the pain expression behaviors in sheep described in the literature, served as a basis for the construction of the scale and then, after content analysis by the committee of experienced researchers in the area, the first instrument was defined including relevant behaviors and excluding irrelevant ones, to make the instrument as simple and representative as possible.

Filming using video cameras adds value to the data as it enables the material to be archived for future research and minimizes the influence of the observer in the evaluation, avoiding possible behavioral changes that the animal may present which are inherent to the presence of the observer. On the other hand, it should be emphasized that in a clinical situation, it is often not possible to make a remote assessment, and the presence of the observer could interfere with the animals' behavior [94–97]. Thus, it is still necessary to validate the proposed scale in clinical situations and with the presence of the observer, to ensure that these results are reproducible.

According to the ethogram, sheep in pain ate less and consequently ruminated less in relation to the moments after rescue analgesia, which justified the introduction of appetite as one of the criteria to be evaluated on the scale. At the preoperative moment, the animals were fasting and there was no feed available, so, obviously this behavior was not present. Previous studies have also shown decreased appetite in sheep submitted to castration and laparoscopy [28,57,73,98], however, sheep in moderate pain may have appetite and rumination [66,99]. The improvement in appetite after analgesic therapy strengthens the need for postoperative analgesia in sheep [100], as in cattle [29].

As reported by the literature [31,62,99,101], in the current study, in sheep in pain, reduced interaction with the environment and activity, low head, and dorsal arching were observed, which confirms that laparoscopy leads to behavioral alterations indicative of acute pain [73]. Some of these behaviors only returned to normal 24 hours after the surgery.

The findings of the ethogram were similar to those in the literature. Lambs submitted to the mulesing procedure were bent over and walked less in the postoperative period [101] and after orchiectomy different behaviors and physiological indicators of postoperative pain were observed [102,103]. Orchiectomy and tail cutting trigger behaviors related to various pain intensities. During severe pain, limb, tail, and head movements and postures with full extension of the pelvic limbs occur; during moderate pain vocalization, standing, sitting, and lying positions with partial extension of the pelvic limbs or with tremor are observed; and during mild pain or no pain, postures may be normal [28].

Vocalization could be a possible indicator of pain, as reported in other ruminants, such as cattle [29,104–106] and goats [106]. However, in sheep, this behavior is more related to social isolation, restraint, and the trans-operative period. Vocalization was not included in the ethogram in the current study, as it was not observed, except at times when feed was supplied. Thus, vocalization is not, in the sheep species, an indicator of postoperative pain, especially in adult animals, even after intense painful stimuli [28,107].

A differential of the current study, which presents advantages and disadvantages compared to others, is that the majority of studies that evaluated acute pain in sheep used lambs [8,28,31–33,62,98,101–103], which could limit extrapolation of the results to adult animals. Some behaviors more specific to lambs mentioned in the literature, such as “jumping like a rabbit”, did not occur, as they are more frequent in young animals, up to about 5 months of age, than in adult animals. A common behavior in cattle [29] and cited in lambs [28,91], looking at the flank and lick the painful area, was not observed in the adult sheep in the current work.

Regarding the validation of the scale, the instrument was subjected to a blinded and random methodology, through the same experimental design already used in cats [47], cattle [29] horses [38], and pigs [30], which presents recognized scientific robustness [71].

Validity and reliability are essential attributes for an instrument to identify and quantify pain in animals. Observers familiar with the behavior of ruminants validated the content of the scale by assessing the representativeness of each item. This analysis measures the extent to which the instrument reflects the phenomenon of interest, in this case, pain [40,47,108,109]. The evaluators were selected for their experience, which improves reliability, that is, the accuracy of the tool based on repeatability and reproducibility [110].

The initially proposed acute pain scale contained 33 variables including items, sub-items and their sub-divisions (S3 Table), which were excluded according to the criteria of the statistical tests (Table 1). This process of refinement of the composite scale contained several steps and identified the 12 most relevant and appropriate variables to measure pain in sheep. The refinement of the pain scale through the analysis of its items was essential to improve the quality of the validated final scale, giving rise to a simpler and more objective version [111].

In a validation of the acute pain scale in lambs, the principal component analysis generated two principal components [28]. Unidimensional scales are not as satisfactory as those with more than one dimension, as they only assess the intensity of pain. However, considering their simplicity and practicality they are easily applicable. In the current study, it is premature to conclude about the dimension of the proposed pain scale, since only one type of pain was evaluated (abdominal), in soft tissues, using only one statistical model. The scale proposed for sheep includes several biological aspects of pain, such as physiological (appetite), sensory or motor (posture, activity), emotional (interaction with other animals and attention in the environment), and temporal (response

to analgesia). Future studies addressing different pain models, such as orthopedic, and other statistical approaches, may confirm the number of dimensions of this scale.

Every new instrument needs to be compared with another already established and validated tool, considered the gold standard [71]. As this is not the case in sheep, since there is no validated scale with robust statistics to assess postoperative pain, the methodology based on the agreement between the pain scores assigned by the evaluators and a gold standard evaluator was adopted, following the same criteria applied in cats [47], cattle [29] horses [38], and pigs [30].

The intra- and inter-observer reliability for each item and for the total score of the sheep scale was similar to that of cattle [29] and pigs [30]. These results were lower than in cats [47] and higher than in horses [38]. When compared with other instruments developed in the sheep species, the scale proposed here presented reliability similar to the scale reported in lambs subjected to acute pain (73% intra and 79% inter-observer reliability) [28]. Compared to a sheep locomotion scale (with a score ranging from 0 to 6) that demonstrated very good intra (91%) and inter-observer (93%) reliability [112], the proposed instrument showed lower results, in which the item “locomotion” presented good intra-observer reliability and only moderate inter-observer reliability. Another study, in which 10 veterinarians and 10 sheep farmers scored a locomotion scale, obtained very good and good values for intra- and inter-observer reliability, however, the reliability for individual locomotion scores varied from reasonable to moderate [113].

Validity indicates that the instrument can accurately measure what is proposed. There are three types of validity. Criterion validity assesses the measuring efficiency of a scale. Two methods may be used: concurrent and predictive criterion validity. Concurrent validity compares the instrument to existing validated scales [43], by evaluating the instrument and the criterion simultaneously and predictive validity evaluates the criterion after the test. Both methods were used in this study [114]. As mentioned earlier, there is no validated instrument to assess postoperative pain in sheep that can be used as a comparison, therefore the proposed instrument was compared

with the NS, SDS, and VAS, when a high correlation was observed. In the sheep species, the correlation of a claudication scale with the NS and VAS was good [115]. Otherwise when comparing the proposed scale with the facial scale in sheep [69], which, although not fully validated, was considered for comparison, the correlation was moderate. The agreement between the gold standard and the other observers was good and this was a second method to assess concurrent criterion validity, as reported previously in other species [29,30,38].

Predictive criterion validity was confirmed by the number of sheep that should receive rescue analgesia based on the Youden Index after surgery (M1). Consistent with this analysis rescue analgesia was indicated in 93% of sheep; therefore the tool would foresee well that sheep were undergoing pain and then be treated, guaranteeing the animals' welfare.

Construct validity reflects the responsiveness of the scale and examines whether the instrument detects predictable differences between groups, or moments [36]. The method tests the hypothesis that time and surgical and analgesic intervention should alter pain scores [47] and has been used to validate scales in veterinary medicine [29,30,38,41,47]. In this study, the differences observed in the pain scores between the moments, and especially at the expected moment of greatest pain compared to the other moments, confirm that the proposed scale is responsive both to identify intense degrees of pain, as well as moderate degrees, which occurred after rescue analgesia, or even mild pain, which occurred 24 hours after surgery. In cattle, the alterations between scores (M2>M4>M3>M1) [29] were different from sheep, where the pain at M4 decreased after M3 (M2>M3>M4>M1), however, the surgical, anesthetic and analgesic protocols were different in the two species of ruminants. The increase and decrease in pain scores after surgery and rescue analgesia, respectively, also occurred in cats [47], horses [38] and pigs [30]. As the current study was carried out with three different breeds of sheep and there was no effect of breed on the scores, apparently the instrument can be used in different sheep breeds.

Principal component analysis relates the variables of the tool in a grouped manner [116] and calculates the number of dimensions determined by different variables [47], to establish the extension or dimensionality of the scale [85]. These variables are related so that the items that define specific parts of the construct are grouped by means of multiple association [117]. According to the Kaiser criterion, one main component was selected and the scale was considered as unidimensional [79]. The scales developed in cattle [29] and pigs [30] are also unidimensional and the scale in cats is multidimensional since it generated three dimensions [45-47]. An instrument is multidimensional when in addition to pain intensity, it includes qualitative and temporal characteristics, such as sensory, motor, emotional, and cognitive dimensions, which have a high correlation in the experience of pain [47,116,117].

All items of the proposed scale presented an acceptable item-total correlation, as in pigs [30], which demonstrates their individual relevance and ensures the homogeneity of the tool. The internal consistency of the proposed scale was excellent, with very similar results to cats - 0.86 [47], cattle - 0.87 [29] and pigs - 0.82 [30], which ensures that the scores of the items that compose the scale can be added and the total score will be representative of the pain intensity [47]. The similarity of the values when excluding each item demonstrates that they have a similar tendency and importance [85].

The scale was specific for all items, however, it was sensitive for only three. Of these three items, the head position and posture values were very close to those considered as good sensitivity (0.70). Only appetite did not present adequate sensitivity, so sheep, even when in pain, can feed, as described in other species [29,30].

The analysis of score distribution, by providing an overview of the occurrence of each score at each moment, indicates the importance of each score. The results were as expected, since the score "0", related to the absence of pain, prevailed before and 24h after the surgery, the scores "1" and "2" occurred more in the postoperative period and after the rescue analgesia, and the score “2” was more evident, especially after surgery, suggesting a greater intensity of pain. The results of each item generally

followed the results of the sum of the scale. Only the score “1” of the “activity” item was not so evident, which demonstrates that it is rare for sheep to move about more than normal or to lie down and get up frequently, as occurs in other species [29,30,38,47].

The analysis of the ROC curve [90] estimated the cut-off point for analgesic intervention in sheep as was the case in pain scales in cats [47], cattle [29] and pigs [30]. The determination of scores indicative of the need to use analgesics helps professionals in clinical decisions, confirms the efficacy of analgesic treatment [47], and prevents unnecessary suffering in animals. The diagnostic uncertainty zone of all evaluators ensures that sheep with a score of > 4 out of a total of 12 points (≥ 5) are really in pain, while those with a score ≤ 2 do not have pain. The low percentage of animals within the zone of diagnostic uncertainty ensures good reliability in making decisions about the indication for rescue analgesia in animals that actually present pain and, therefore, should receive analgesia. Thus, the proposed scale presents excellent diagnostic accuracy. Although the definition of the score referring to the analgesic intervention point is a good tool, it is emphasized that even if the scores are < 4, in some cases additional analgesia may be necessary according to the clinical evaluation, at the discretion of the observer. The cut-off point was > 4/10 in cattle [29] , > 4/18 in pigs [30], and for the subscale “expression of pain” in cats it was > 2/12 [47]. In a recent empirical study, pain in sheep after cardiac surgery was scored; for scores of 0-2/25 there was no intervention, 3-9/25 rescue analgesia was performed, and ≥10/25 multimodal analgesia was performed [118]. Another study subjected 18 sheep to laparoscopy and pain was assessed for 24 hours based on three items: decreased appetite, limited mobility, and back arching, but the pain scale ranging from 0 to 6 was insensitive, with 90% of animals with a “0” score and 10% with “1” [98].

In line with the low percentage of animals within the diagnostic uncertainty zone, the high areas under the curve observed in this study (> 0.95) indicate that the scale has high discriminatory capacity, that is, it correctly classifies individuals with or without pain

[88], results that resembled cattle [29], pigs [30] and the subscale “expression of pain” in in cats [47],

To our knowledge, the scales that assess acute pain in various animal species do not classify pain intensity based on their scores, except in an empirical way [44]. In this study, the zone of diagnostic uncertainty (3 - 4) corresponded to the lower limit of moderate pain scores (4), insuring that sheep suffering from moderate pain would be treated according to the cut-off point.

Limitations

The current study had some limitations. The main one is that the behavioral scale was validated only for a specific type of soft tissue surgery (abdominal - laparoscopy) and in females. Further studies are needed to test this tool in different procedures, such as orthopedic surgery and in clinical circumstances, to ensure its versatility. To establish that the instrument is valid under field conditions, clinical validation with less experienced observers is also required. Since the majority of the studies that evaluated acute pain in sheep were in lambs, this can limit the collation of data, which means the instrument needs to be tested in lambs.

Another limitation is that video analysis does not necessarily equate to presential analysis. Video observation has the disadvantage of lacking some details observed in real time, while, as an advantage it can be reviewed. According to the previous experience of the authors, video analysis provides material to develop the ethogram and is an important step to validate a scale, as reported in cats [47], cattle [29] horses [38], and pigs [30], especially to assess intra-observer reliability. According to a study by our group in cats, the results of the video analysis were also reproducible in a clinical setting [45–47].

Some limitations relate specifically to the videos. Although the study was blinded, some videos may have suggested the moment they were taken: at baseline, the sheep were fasting, with no available feed, hence it was difficult for the observers to interpret if

the animals did not eat due to lack of food or if they really had anorexia. To overcome this problem and avoid that appetite would be excluded according to the refinement criteria, appetite was arbitrarily considered normal before surgery regardless of the scores attributed by the observers. Around 21% of the videos at M3 were filmed at night with artificial light, which could suggest that they corresponded to M3; variations in the circadian cycle could alter some behaviors such as activity, so the reduction in activity observed at night may not be related to pain or discomfort, but to the natural reduction in activity at night [119]; given the small difference in the size of the stalls, the density of animals varied slightly, which could influence interaction and locomotion behaviors; the dark wool of some animals may also have made it difficult to evaluate some items in the videos/photos, making the analysis less accurate, especially on the facial scale.

To improve data reliability, the authors suggest that observers attend a training period, as, at least in laboratory animals, instruction and training have improved pain recognition [120].

Conclusion

It is concluded that, after refinement of the originally proposed scale, the Unesp-Botucatu composite scale to assess acute postoperative pain in sheep (USAPS) is a valid, reliable, specific and sensitive instrument, with excellent internal consistency and discriminatory capacity. The well-defined cut-off point for rescue analgesia and the classification of pain intensity supports the indication and type of analgesic therapy. To assess the clinical and experimental applicability of the scale and ensure its versatility, it is recommended that it be evaluated in other surgical procedures and in lambs.

Acknowledgements

Capes scholarship and FAPESP thematic research project number 2017/12815-0.

References

1. Huxley JN, Whay HR. Current attitudes of cattle practitioners to pain and the use of analgesics in cattle. Vet Rec. 2006;159: 662–668. doi:10.1136/vr.159.20.662
2. Flecknell P. Analgesia from a veterinary perspective. Br J Anaesth. 2008;101: 121–124. doi:10.1093/bja/aen087
3. Lizarraga I, Chambers JP. Use of analgesic drugs for pain management in sheep. N Z Vet J. 2012;60: 87–94. doi:10.1080/00480169.2011.642772
4. Lorena SERS, Luna SPL, Lascelles BDX, Corrente JE. Attitude of brazilian veterinarians in the recognition and treatment of pain in horses and cattle. Vet Anaesth Analg. 2013;40: 410–418. doi:10.1111/vaa.12025
5. Raekallio M, Heinonen KM, Kuussaari J, Vainio O. Pain alleviation in animals: attitudes and practices of finnish veterinarians. Vet J. 2003;165: 131–135.
6. Anil L, Anil SS, Deen J. Detection P. Pain detection and amelioration in animals on the farm: issues and options. J Appl Anim Welf Sci. 2005;8(4):261-78. doi: 10.1207/s15327604jaws0804_3
7. Hewson CJ, Dohoo IR, Lemke KA, Barkema HW. Canadian veterinarians' use of analgesics in cattle, pigs, and horses in 2004 and 2005. Can Vet J = La Rev Vet Can. 2007;48: 155–164. Available from: https://www.ncbi.nlm.nih.gov/pubmed/17334029
8. Futro A, Masłowska K, Dwyer CM. Ewes direct most maternal attention towards lambs that show the greatest pain-related behavioural responses. PLoS One. 2015;10: 1–15. doi:10.1371/journal.pone.0134024
9. West D, Bruere N, Ridler A. The sheep: health disease and production. 4th ed. New Zealand: Massey University Press; 2018.

10. Dolan S, Field LC, Nolan AM. The role of nitric oxide and prostaglandin signaling pathways in spinal nociceptive processing in chronic inflammation. Pain. 2000;86: 311–320.

11. Kaler J, Medley GF, Grogono-Thomas R, Wellington EMH, Calvo-Bado LA, Wassink GJ, et al. Factors associated with changes of state of foot conformation and lameness in a flock of sheep. Prev Vet Med. 2010;97: 237–244. doi:10.1016/j.prevetmed.2010.09.019

12. Ley SJ, Livingston A, Waterman AE. The effect of chronic clinical pain on thermal and mechanical thresholds in sheep. Pain. 1989;39: 353–357. doi:10.1016/0304-3959(89)90049-3

13. Ley SJ, Waterman AE, Livingston A. A field study of the effect of lameness on mechanical nociceptive thresholds in sheep. Vet Rec. 1995;137: 85–87. doi:10.1136/vr.137.4.85

14. Dolan S, Kelly JG, Monteiro AM, Nolan AM. Up-regulation of metabotropic glutamate receptor subtypes 3 and 5 in spinal cord in a clinical model of persistent inflammation and hyperalgesia. Pain. 2003;106: 501–512.

15. Taylor K, Rego L. EU statistics on animal experiments for 2014. Altern to Anim Exp. 2016;33. doi:10.14573/altex.1609291

16. Gigliuto C, De Gregori M, Malafoglia V, Raffaeli W, Compagnone C, Visai L, et al. Pain assessment in animal models: do we need further studies? J Pain Res. 2014;7: 227–236. doi:10.2147/JPR.S59161

17. Dias IR, Camassa JA, Bordelo JA, Babo PS, Viegas CA, Dourado N, et al. Preclinical and translational studies in small ruminants (sheep and goat) as models for osteoporosis research. Curr Osteoporos Rep. 2018;16: 182–197. doi:10.1007/s11914-018-0431-2

18. Sartoretto SC, Uzeda MJ, Miguel FB, Nascimento JR, Ascoli F, Calasans-Maia MD. Sheep as an experimental model for biomaterial implant evaluation. Acta Ortop Bras. 2016;24: 262–266. doi:10.1590/1413-785220162405161949

19. Dolan S, Gunn MD, Crossan C, Nolan AM. Activation of metabotropic glutamate receptor 7 in spinal cord inhibits pain and hyperalgesia in a novel formalin model in sheep. Behav Pharmacol. 2011;22: 582–588. doi:10.1097/FBP.0b013e3283478802

20. Wilkes D, Li G, Angeles CF, Patterson JT, Huang LYM. A large animal neuropathic pain model in sheep: A strategy for improving the predictability of preclinical models for therapeutic development. J Pain Res. 2012;5: 415–424. doi:10.2147/JPR.S34977

21. Stubsjøen SM, Bohlin J, Skjerve E, Valle PS, Zanella AJ. Applying fractal analysis to heart rate time series of sheep experiencing pain. Physiol Behav. 2010;101: 74–80. doi:10.1016/j.physbeh.2010.04.018

22. Ong RM, Morris JP, O'Dwyer JK, Barnett JL, Hemsworth PH, Clarke IJ. Behavioural and EEG changes in sheep in response to painful acute electrical stimuli. Aust Vet J. 1997;75: 189–193. doi:10.1111/j.1751-0813.1997.tb10064.x

23. Mather LE, Cousins MJ, Huang YF, Pryor ME, Barratt SM. Lack of secondary hyperalgesia and central sensitization in an acute sheep model. Reg Anesth Pain Med. 2000;25: 174–180. doi:10.1097/00115550-200004000-00008

24. McLennan KM, Skillings EA, Rebelo CJB, Corke MJ, Pires Moreira MA, Morton AJ, et al. Technical note: Validation of an automatic recording system to assess behavioural activity level in sheep (Ovis aries). Small Rumin Res. 2015;127: 92–96. doi:10.1016/j.smallrumres.2015.04.002

25. Stubsjøen SM, Bohlin J, Dahl E, Knappe-Poindecker M, Fjeldaas T, Lepschy M, et al. Assessment of chronic stress in sheep (part I): The use of cortisol and cortisone

in hair as non-invasive biological markers. Small Rumin Res. 2015;132: 25–31. doi:10.1016/j.smallrumres.2015.09.015

26. Stubsjøen SM, Knappe-Poindecker M, Langbein J, Fjeldaas T, Bohlin J. Assessment of chronic stress in sheep (part II): Exploring heart rate variability as a non-invasive measure to evaluate cardiac regulation. Small Rumin Res. 2015;133: 30–35. doi:10.1016/j.smallrumres.2015.10.026

27. Jongman EC, Morris JP, Barnett JL, Hemsworth PH.. EEG changes in 4-week-old lambs in response to castration, tail docking and mulesing. Aust Vet J. 2000;78: 339–343. doi:10.1111/j.1751-0813.2000.tb11789.x

28. Molony V, Kent JE, McKendrick IJ. Validation of a method for assessment of an acute pain in lambs. Appl Anim Behav Sci. 2002;76: 215–238. doi:10.1016/S0168-1591(02)00014-X

29. de Oliveira FA, Luna SPL, do Amaral JB, Rodrigues KA, Sant'Anna AC, Daolio M, et al. Validation of the UNESP-Botucatu unidimensional composite pain scale for assessing postoperative pain in cattle. BMC Vet Res. 2014;10: 1–14. doi:10.1186/s12917-014-0200-0

30. Luna, S; Araujo, A; Neto, P; Oliveira, F; Brondani, J; Telles, F; Azeredo I. Validation of the UNESP-Botucatu unidimensional composite pain scale for assessing postoperative pain in pigs. 12th World Congress of Veterinary Anestesiology. Kyoto; 2015.

31. Molony V, Kent JE. Assessment of acute pain in farm animals using behavioral and physiological measurements. J Anim Sci. 1997;75: 266–272. doi:10.2527/1997.751266x

32. Grant C. Behavioural responses of lambs to common painful husbandry procedures. Appl Anim Behav Sci. 2004;87: 255–273. doi:https://doi.org/10.1016/j.applanim.2004.01.011

33. Guesgen MJ, Beausoleil NJ, Stewart M. Effects of early human handling on the pain sensitivity of young lambs. Vet Anaesth Analg. 2013;40: 55–62. doi:10.1111/j.1467-2995.2012.00746.x

34. Morton CM, Reid J, Scott EM, Holton LL, Nolan AM. Application of a scaling model to establish and validate an interval level pain scale for assessment of acute pain in dogs. Am J Vet Res. 2005;66: 2154–2166. doi:10.2460/ajvr.2005.66.2154

35. McDowell I. Measuring health: A guide to rating scales and questionnaires 3rd ed. New York: Oxford University Press; 2006. doi: 10.1093/acprof:oso/9780195165678.001.0001

36. Crellin D, Sullivan TP, Babl FE, O'Sullivan R, Hutchinson A. Analysis of the validation of existing behavioral pain and distress scales for use in the procedural setting. Paediatr Anaesth. 2007;17: 720–733. doi:10.1111/j.1460-9592.2007.02218.x

37. Beyer JE, Wells N. The assessment of pain in children. Pediatr Clin North Am. 1989;36: 837–854.

38. Taffarel MO, Luna SPL, de Oliveira FA, Cardoso GS, Alonso JM, Pantoja JC, et al. Refinement and partial validation of the UNESP-Botucatu multidimensional composite pain scale for assessing postoperative pain in horses. BMC Vet Res. 2015;11. doi:10.1186/s12917-015-0395-8

39. von Baeyer CL, Spagrud LJ. Systematic review of observational (behavioral) measures of pain for children and adolescents aged 3 to 18 years. Pain. 2007;127: 140–150. doi:10.1016/j.pain.2006.08.014

40. Bussieres G, Jacques C, Lainay O, Beauchamp G, Leblond A, Cadore J-L, et al. Development of a composite orthopaedic pain scale in horses. Res Vet Sci. 2008;85: 294–306. doi:10.1016/j.rvsc.2007.10.011

41. Brondani JT, Luna SPL, Minto BW, Santos BPR, Beier SL, Matsubara LM, et al. Validade e responsividade de uma escala multidimensional para avaliação de dor pós-operatória em gatos. Arq Bras Med Veterinária e Zootec. 2012;64: 1529–1538. doi:10.1590/S0102-09352012000600019

42. Firth AM, Haldane SL. Development of a scale to evaluate postoperative pain in dogs. J Am Vet Med Assoc. 1999;214: 651–659.

43. Holton L, Reid J, Scott EM, Pawson P, Nolan A. Development of a behaviour-based scale to measure acute pain in dogs. Vet Rec. 2001;148: 525–531. doi:10.1136/vr.148.17.525

44. Reid J, Nolan AM, Hughes JML, Lascelles D, Pawson P, Scott EM. Development of the short-form Glasgow composite measure pain scale (CMPS-SF) and derivation of an analgesic intervention score. Anim Welf. 2007;16: 97–104.

45. Brondani JT, Luna SPL, Padovani CR. Refinement and initial validation of a multidimensional composite scale for use in assessing acute postoperative pain in cats. Am J Vet Res. 2011;72: 174–183. doi:10.2460/ajvr.72.2.174

46. Brondani JT, Luna S, Padovani CR. Assessing acute postoperative pain in cats. Am J Vet Res. 2011;72: 174–183. doi:10.2460/ajvr.72.2.174

47. Brondani JT, Mama KR, Luna SPL, Wright BD, Niyom S, Ambrosio J, et al. Validation of the english version of the UNESP-Botucatu multidimensional composite pain scale for assessing postoperative pain in cats. BMC Vet Res. 2013;9: 1. doi:10.1186/1746-6148-9-143

48. Graubner C, Gerber V, Doherr M, Spadavecchia C. Clinical application and reliability of a post abdominal surgery pain assessment scale (PASPAS) in horses. Vet J. 2011;188: 178–183. doi:10.1016/j.tvjl.2010.04.029

49. Sutton GA, Paltiel O, Soffer M, Turner D. Validation of two behaviour-based pain scales for horses with acute colic. Vet J. 2013;197: 646–650. doi:10.1016/j.tvjl.2013.04.007

50. Sutton GA, Dahan R, Turner D, Paltiel O. A behaviour-based pain scale for horses with acute colic: scale construction. Vet J. 2013;196: 394–401. doi:10.1016/j.tvjl.2012.10.008

51. Banks EM. Behavioral research to answer questions about animal welfare. J Anim Sci. 1982;54: 434–446. doi:10.2527/jas1982.542434x

52. Thornton PD, Waterman-Pearson AE. Quantification of the pain and distress responses to castration in young lambs. Res Vet Sci. 1999;66: 107–118. doi:10.1053/rvsc.1998.0252

53. Dwyer CM, Lawrence AB. Maternal behaviour in domestic sheep (ovis aries): Constancy and change with maternal experience. Behaviour. 2000;137: 1391–1413. doi:10.1163/156853900501999

54. Thornton PD, Waterman-Pearson AE. Behavioural responses to castration in lambs. Anim Welf. 2002;11: 203–212.

55. Mellema SC, Doherr MG, Wechsler B, Thueer S, Steiner A. Influence of local anaesthesia on pain and distress induced by two bloodless castration methods in young lambs. Vet J. 2006;172: 274–283. doi:10.1016/j.tvjl.2005.06.002

56. Melches S, Mellema SC, Doherr MG, Wechsler B, Steiner A. Castration of lambs: a welfare comparison of different castration techniques in lambs over 10 weeks of age. Vet J. 2007;173: 554–563. doi:10.1016/j.tvjl.2006.01.006

57. Molony V, Kent JE, Viñuela-Fernández I, Anderson C, Dwyer CM. Pain in lambs castrated at 2days using novel smaller and tighter rubber rings without and with local anaesthetic. Vet J. 2012;193: 81–86. doi:10.1016/j.tvjl.2011.09.030

58. Guesgen MJ, Beausoleil NJ, Minot EO, Stewart M, Stafford KJ, Morel PCH. Lambs show changes in ear posture when experiencing pain. Anim Welf. 2016;25: 171–177. doi:10.7120/09627286.25.2.171

59. Small AH, Belson S, Holm M, Colditz IG. Efficacy of a buccal meloxicam formulation for pain relief in Merino lambs undergoing knife castration and tail docking in a randomised field trial. Aust Vet J. 2014;92: 381–388. doi:10.1111/avj.12241

60. McGlone JJ, Stobart RH. A quantitative ethogram of behavior of yearling ewes during two hours post-parturition. Appl Anim Behav Sci. 1986;16: 157–164. doi:10.1016/0168-1591(86)90108-5

61. Mellor DJ, Murray L. Effects of tail docking and castration on behaviour and plasma cortisol concentrations in young lambs. Res Vet Sci. 1989;46: 387–391.

62. Molony V, Kent JE, Robertson IS. Behavioural responses of lambs of three ages in the first three hours after three methods of castration and tail docking. Res Vet Sci. 1993;55: 236–245.

63. Kent JE, Molony V, Robertson IS. Comparison of the burdizzo and rubber ring methods for castrating and tail docking lambs. Vet Rec. 1995;136: 192–196. doi:10.1136/vr.136.8.192

64. Lester SJ, Mellor DJ, Holmes RJ, Ward RN, Stafford KJ. Behavioural and cortisol responses of lambs to castration and tailing using different methods. N Z Vet J. 1996;44: 45–54. doi:10.1080/00480169.1996.35933

65. Graham MJ, Kent JE, Molony V. Effects of four analgesic treatments on the behavioural and cortisol responses of 3-week-old lambs to tail docking. Vet J. 1997;153: 87–97.

66. Kent JE, Molony V, Graham MJ. Comparison of methods for the reduction of acute pain produced by rubber ring castration or tail docking of week-old lambs. Vet J. 1998;155: 39–51. doi:10.1016/S1090-0233(98)80033-6

67. Taylor KD, Mills DS. The development and assessment of temperament tests for adult companion dogs. J Vet Behav. 2006;1: 94–108. doi: 10.1016/j.jveb.2006.09.002

68. Belshaw Z, Asher L, Harvey ND, Dean RS. Quality of life assessment in domestic dogs: an evidence-based rapid review. Vet J. 2015;206: 203–212. doi:10.1016/j.tvjl.2015.07.016

69. McLennan KM, Rebelo CJB, Corke MJ, Holmes MA, Leach MC, Constantino-Casas F. Development of a facial expression scale using footrot and mastitis as models of pain in sheep. Appl Anim Behav Sci. 2016;176: 19–26. doi:10.1016/j.applanim.2016.01.007

70. Häger C, Biernot S, Buettner M, Glage S, Keubler LM, Held N, et al. The Sheep Grimace Scale as an indicator of post-operative distress and pain in laboratory sheep. PLoS One. 2017;12: e0175839. doi:10.1371/journal.pone.0175839

71. Merola I, Mills DS. Systematic review of the behavioural assessment of pain in cats. J Feline Med Surg. 2016;18: 60–76. doi:10.1177/1098612X15578725

72. Kilkenny C, Browne W, Cuthill IC, Emerson M, Altman DG. Animal research: reporting in vivo experiments: the ARRIVE guidelines. Br J Pharmacol. 2010;160: 1577–1579. doi:10.1111/j.1476-5381.2010.00872.x

73. Teixeira PPMM, Padilha LC, Motheo TF, Silva MAMM, Oliveira MEFF, da Silva ASLL, et al. Ovariectomy by laparotomy, a video-assisted approach or a complete laparoscopic technique in Santa Ines sheep. Small Rumin Res. 2011;99: 199–202. doi:https://doi.org/10.1016/j.smallrumres.2011.04.008

74. Rodriguez M, Ambrogi M, Dayane V, Maciel G, Feliciano M, Teixeira P. Aspiração folicular por laparoscopia em ovinos. Rev Investig. 2015;14: 55–60. doi:10.26843/investigacao.v14i2.878

75. de Araujo EAB, de Oliveira SN, de Faria Tabet A, Bittencourt RF, Chalhoub M, Filho ALR. Aspiração folicular videolaparoscópica comparativa em ovelhas Dorper e Santa Inês. Cienc Anim Bras. 2016;17: 98–104. doi:10.1590/1089-6891v17i128347

76. Martin P, Bateson PPG. Measuring behaviour: An introductory guide, 2nd ed. New York, NY, US: Cambridge University Press; 1993. doi:10.1017/CBO9781139168342

77. RstudioTeam. RStudio Team. 2016. Available: http://www.rstudio.com/

78. Suraseranivongse S, Santawat U, Kraiprasit K, Petcharatana S, Prakkamodom S, Muntraporn N. Cross-validation of a composite pain scale for preschool children within 24 hours of surgery. BJA Br J Anaesth. 2001;87: 400–405. doi:10.1093/bja/87.3.400

79. Schuster C. A note on the interpretation of weighted kappa and its relations to other rater agreement statistics for metric scales. Educ Psychol Meas. 2004;64: 243–253. doi:10.1177/0013164403260197

80. Landis JR, Koch GG. The measurement of observer agreement for categorical data. Biometrics. 1977;33: 159–174.

81. Cohen J. Weighted kappa: nominal scale agreement with provision for scaled disagreement or partial credit. Psychol Bull. 1968;70: 213–220.

82. Altman DG. Practical statistics for medical research. 1st ed. London: Chapman and Hall; 1991. doi: 10.1002/sim.4780101015

83. Kaiser HF. The varimax criterion for analytic rotation in factor analysis. Psychometrika. 1958;23: 187–200. doi:10.1007/BF02289233

84. Streiner D, Norman G, Cairney J. Health measurement scales: A practical guide to their development and use. 5th ed. New York: Oxford University Press Inc; 2015. doi:10.1093/acprof:oso/9780199231881.003.0006

85. Jensen MP. Questionnaire validation: a brief guide for readers of the research literature. Clin J Pain. 2003;19: 345–352.

86. Streiner DL. Starting at the beginning: an introduction to coefficient alpha and internal consistency. J Pers Assess. 2003;80: 99–103. doi:10.1207/S15327752JPA8001_18

87. Deyo RA, Diehr P, Patrick DL. Reproducibility and responsiveness of health status measures statistics and strategies for evaluation. Control Clin Trials. 1991;12: 142–158. doi:10.1016/S0197-2456(05)80019-4

88. Streiner DL, Cairney J. What's under the ROC? An introduction to receiver operating characteristics curves. Can J Psychiatry. 2007;52: 121–128. doi:10.1177/070674370705200210

89. Mallat J, Meddour M, Durville E, Lemyze M, Pepy F, Temime J, et al. Decrease in pulse pressure and stroke volume variations after mini-fluid challenge accurately predicts fluid responsiveness. Br J Anaesth. 2015;115: 449–456. doi:10.1093/bja/aev222

90. Cannesson M, Le Manach Y, Hofer CK, Goarin JP, Lehot J-J, Vallet B, et al. Assessing the diagnostic accuracy of pulse pressure variations for the prediction of fluid responsiveness: a “gray zone” approach. Anesthesiology. 2011;115: 231–241. doi:10.1097/ALN.0b013e318225b80a

91. Gall O, Champigneulle B, Schweitzer B, Deram T, Maupain O, Montmayeur Verchere J, et al. Postoperative pain assessment in children: a pilot study of the usefulness of the analgesia nociception index. Br J Anaesth. 2015;115: 890–895. doi:10.1093/bja/aev361

92. Celeita-Rodríguez N, Teixeira-Neto FJ, Garofalo NA, Dalmagro TL, Girotto CH, Oliveira GCV, et al. Comparison of the diagnostic accuracy of dynamic and static preload indexes to predict fluid responsiveness in mechanically ventilated, isoflurane anesthetized dogs. Vet Anaesth Analg. 2019;46: 276–288. doi:10.1016/j.vaa.2018.12.004

93. Trindade PHE, Hartmann E, Keeling LJ, Andersen PH, Ferraz GC, da Costa MJRP. Effect of work on body language of ranch horses in Brazil. Loor JJ, editor. PLoS One. 2020;15: e0228130. doi:10.1371/journal.pone.0228130

94. Pinho RH, Leach MC, Rocha FDL, Armani DJC, Ferreira-Souza G, Dissenha A, et al. Influence of the presence or absence of an observer on pain assessment in rabbits submitted to orthopaedic surgery. AVA autumn meeting. Ghent; 2019. p. 120.

95. Sorge RE, Martin LJ, Isbester KA, Sotocinal SG, Rosen S, Tuttle AH, et al. Olfactory exposure to males, including men, causes stress and related analgesia in rodents. Nat Methods. 2014;11:629–32. doi:10.1038/nmeth.2935

96. Leach MC, Allweiler S, Richardson C, Roughan J V, Narbe R, Flecknell PA. Research in veterinary science behavioural effects of ovariohysterectomy and oral

administration of meloxicam in laboratory housed rabbits. Res Vet Sci. 2009;87: 336–347. doi:10.1016/j.rvsc.2009.02.001

97. Lester LS, Fanselow MS. Exposure to a Cat Produces Opioid Analgesia in Rats. Behav Neurosci.1985;99: 756–759.

98. Adams J. Assessment and management of pain in small ruminants. Livestock. 2017;22: 324–328. doi:10.12968/live.2017.22.6.324

99. Manteca X, Temple D, Mainau E, Llonch P. Évaluation de la douleur chez les ovins. Farm animal welfare education centre. janvier 2017; 1–2. Available from: https://www.fawec.org/media/com_lazypdf/pdf/Fiche_Technique_FAWEC_n17_fr.pdf

100. Teixeira PPM, Padilha LC, Oliveira MEF, Motheo TF, da Silva ASL, Barros FFPC, et al. Laparoscopic ovum collection in sheep: gross and microscopic evaluation of the ovary and influence on ooctye production. Anim Reprod Sci. 2011;127: 169–175. doi:10.1016/j.anireprosci.2011.08.001

101. Paull DR, Lee C, Atkinson SJ, Fisher AD. Effects of meloxicam or tolfenamic acid administration on the pain and stress responses of Merino lambs to mulesing. Aust Vet J. 2008;86: 303–311. doi:10.1111/j.1751-0813.2008.00325.x

102. Mellor DJ, Stafford KJ, Todd SE, Lowe TE, Gregory NG, Bruce RA, et al. A comparison of catecholamine and cortisol responses of young lambs and calves to painful husbandry procedures. Aust Vet J. 2002;80: 228–233. doi:10.1111/j.1751-0813.2002.tb10820.x

103. Dinniss AS, Stafford KJ, Mellor DJ, Bruce RA, Ward RN. The behaviour pattern of lambs after castration using a rubber ring and/or castrating clamp with or without local anaesthetic. N Z Vet J. 1999;47: 198–203. doi:10.1080/00480169.1999.36143

104. Currah JM, Hendrick SH, Stookey JM. The behavioral assessment and alleviation of pain associated with castration in beef calves treated with flunixin meglumine and caudal lidocaine epidural anesthesia with epinephrine. Can Vet J = La Rev Vet Can. 2009;50: 375–382.

105. Millman ST. Behavioral responses of cattle to pain and implications for diagnosis, management, and animal welfare. Vet Clin North Am Food Anim Pract. 2013;29: 47–58. doi:10.1016/j.cvfa.2012.11.007

106. Watts, Stookey. Vocal behaviour in cattle: the animal's commentary on its biological processes and welfare. Appl Anim Behav Sci. 2000;67: 15–33. doi:10.1016/s0168-1591(99)00108-2

107. Ferguson DM, Lee C, Fisher A. Advances in sheep welfare. 1st ed. Melbourne: Woodhead publishing; 2017. doi:10.1016/C2015-0-00983-2

108. Ison SH, Clutton RE, Di Giminiani P, Rutherford KMD. A review of pain assessment in pigs. Front Vet Sci. 2016. p.108. doi: 10.3389/fvets.2016.00108

109. Murrell JC, Psatha EP, Scott EM, Reid J, Hellebrekers LJ. Application of a modified form of the Glasgow pain scale in a veterinary teaching centre in the Netherlands. Vet Rec. 2008;162: 403–408. doi:10.1136/vr.162.13.403

110. DeVon HA, Block ME, Moyle-Wright P, Ernst DM, Hayden SJ, Lazzara DJ, et al. A psychometric toolbox for testing validity and reliability. J Nurs Scholarsh. 2007;39: 155–164. doi:10.1111/j.1547-5069.2007.00161.x

111. Boateng GO, Neilands TB, Frongillo EA. Best practices for developing and validating scales for health, social, and behavioral research: a primer. 2018;6: 1–18. doi:10.3389/fpubh.2018.00149

112. Kaler J, Wassink GJ, Green LE. The inter- and intra-observer reliability of a locomotion scoring scale for sheep. 1129 Vet J. 2009;180(2):189–94.

113. Angell JW, Cripps PJ, Grove-White DH, Duncan JS. A practical tool for locomotion scoring in sheep: reliability when used by veterinary surgeons and sheep farmers. Vet Rec. 2015;176: 521. doi:10.1136/vr.102882

114. Pasquali L. Psychometrics. Ver. esc. enferm.USP. 2009;43: 992–999. doi:10.1590/S0080-62342009000500002

115. Welsh EM, Gettinby G, Nolan AM. Comparison of a visual analogue scale and a numerical rating scale for assessment of lameness, using sheep as a model. Am J Vet Res. 1993;54: 976–983.

116. Gracely RH. Evaluation of multi-dimensional pain scales. Pain. 1992; 48: 297–300. doi:10.1016/0304-3959(92)90076-n

117. Netemeyer R, Bearden W, Sharma S. Scaling procedures: issues and applications. London: Sage Publications; 2003. doi:10.4135/9781412985772

118. Izer JM, LaFleur RA, Weiss WJ, Wilson RP. Development of a pain scoring system for use in sheep surgically implanted with ventricular assist devices. J Investig Surg. 2018;1939: 1–10. doi:10.1080/08941939.2018.1457191

119. Wyse CA, Zhang X, McLaughlin M, Biello SM, Hough D, Bellingham M, et al. Circadian rhythms of melatonin and behaviour in juvenile sheep in field conditions: Effects of photoperiod, environment and weaning. Physiol Behav. 2018;194: 362–370. doi:10.1016/j.physbeh.2018.06.001

120. Roughan J V, Flecknell PA. Evaluation of a short duration behaviour-based post-operative pain scoring system in rats. Eur J Pain. 2003;7: 397–406. doi:10.1016/S1090-3801(02)00140-4

Supporting information

S1 Table. Ethogram with the description of the behaviors analyzed in 48 sheep submitted to laparoscopy [8,28,31,32,52–66].

S2 Table. **Criteria used to select the behaviors included in the pre-refinement Unesp Botucatu scale used for video analysis (S3 Table), based on content validity and behaviors reported in the literature.**

S3 Table. Pre-refinement Unesp-Botucatu scale to assess postoperative pain in sheep submitted to video analysis after content validation.

Ev. = Evaluator; each item was classified as relevant (+1), not known (0) or irrelevant (-1), the values were added and divided by the number of experts. Score: final of content validation. In bold are the items approved in content validation, because mean score was ≥ 0.5 and because the specific pain-related behavior had been reported in the ethogram (x) and literature [78];in italics are the behaviors that remained on the scale after refinement; "-" = behaviors that did not occur at any moment

S4 Table. Refinement process for inclusion and exclusion of items and sub-items on the Unesp-Botucatu scale to assess acute postoperative pain

Statistical tests: Etho. M2 - highest score of behavior in the Ethogram according the Friedman test at M2 vs the other moments; % M2 ≥ 15 - at least 15% frequency of occurrence of items/sub-items at M2; CV - content validation; Intra – intra-observer reliability; Inter – inter-observer reliability; PCA - Principal component analysis; F (M) - Friedman test between moments; ITC - item-total correlation; IC - Internal consistency; Sp - Specificity; S – Sensitivity. Sum – sum of the 10 statistical tests (Friedman test was divided into 3 tests). Locomotion: 1 - Difficulty and/or reluctance to get up; 2 - Does not move and/or walks abnormally and/or with a limp and/or when standing, the limbs may be more open and further back than normal; 3 - Leans against a surface; Posture: A - Kicks or stamps one or more limbs on the ground; B1 - Extends the head and neck; B2 - Extends one or more limbs; C – In ventral decubitus, the head rests on the ground or is close to the ground in column line; Miscellaneous behaviors: A - Moves the tail quickly and repeatedly and/or keeps the tail straight; B - Arched back; C - Body tremors (without considering the ears); D. Crawls in ventral decubitus, without getting up. Moments - M1: preoperative; M2: postoperative, before rescue analgesia; M3: postoperative, after rescue analgesia; M4: 24h postoperative. 1 - item included according to the criteria of each test. In bold the Itens and subitens included in final scale after refinement; The main items were submitted to 12 tests and a minimum of 7 were included in the final scale (Table 3); sub-items were subjected to 5 tests, and a minimum of 3 were included in the final scale (Table 3).

Fig 1

Click here to download Figure Fig 1.tif

**Moments**<br><br>Filming	**M1**<br>1h before surgery	**Anesthesia** (Diazepam + Ketamine IV)<br>**Local anesthesia:** Lidocaine (epidural)	**Surgery**	**M2**<br>3-4h after surgery	**Rescue Analgesia** (Meloxicam + Morphine IV)	**M3**<br>1h after rescue analgesia	**M4**<br>24 h after surgery

Fig 2 Click here to download Figure Fig 2.tif

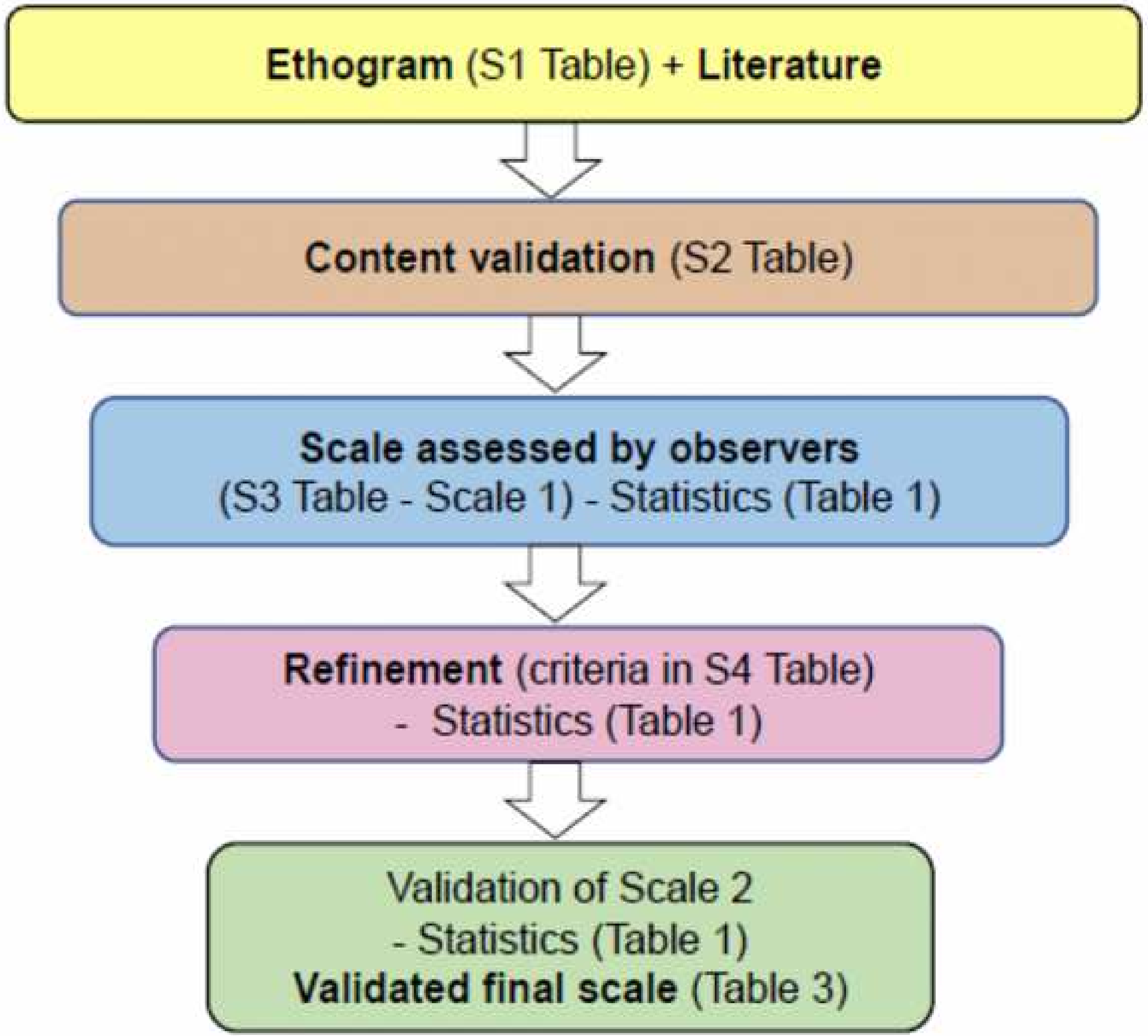

Fig 3

Click here to download Figure Fig. 3.tif

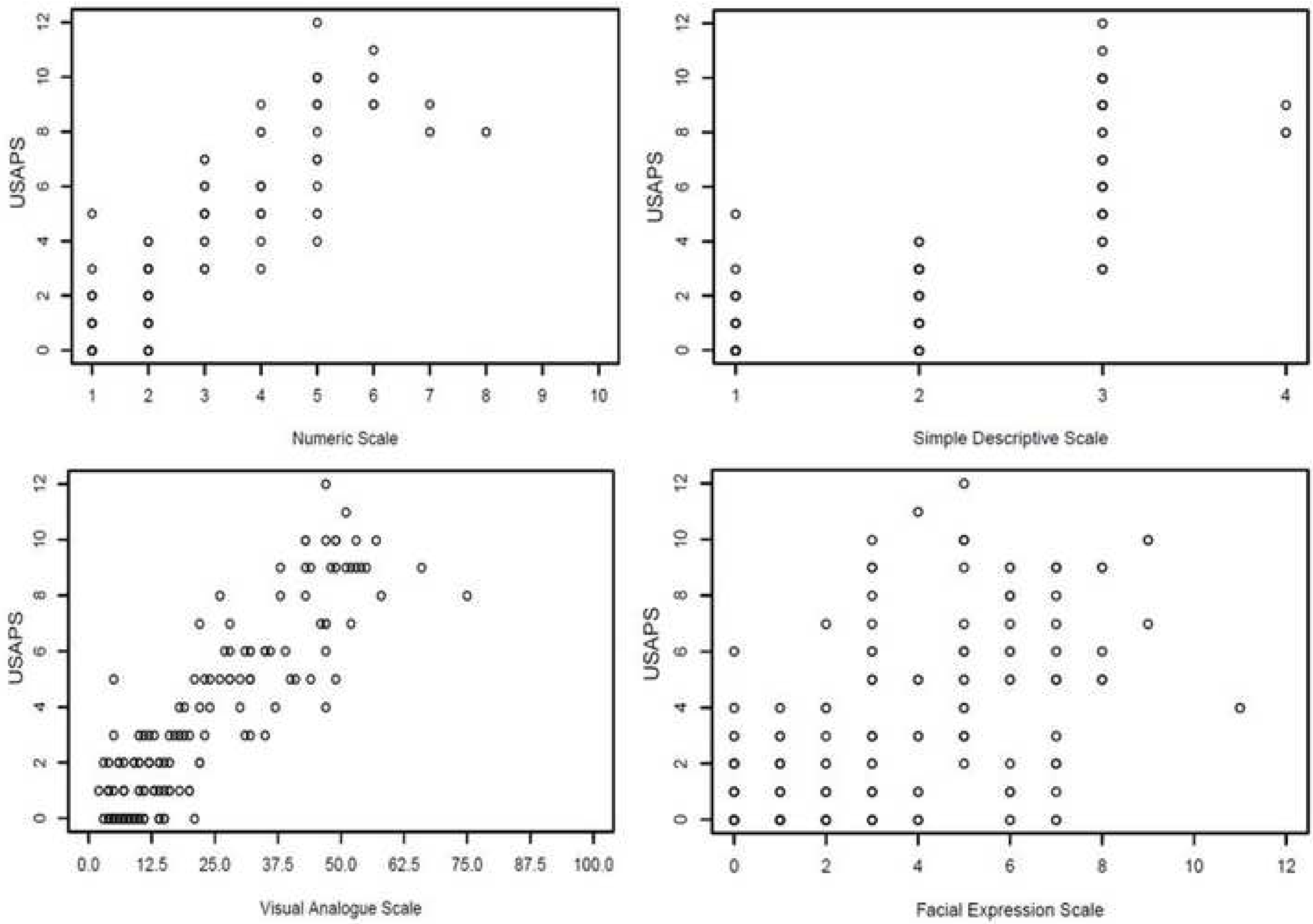

Fig 4

Click here to download Figure Fig. 4.tif

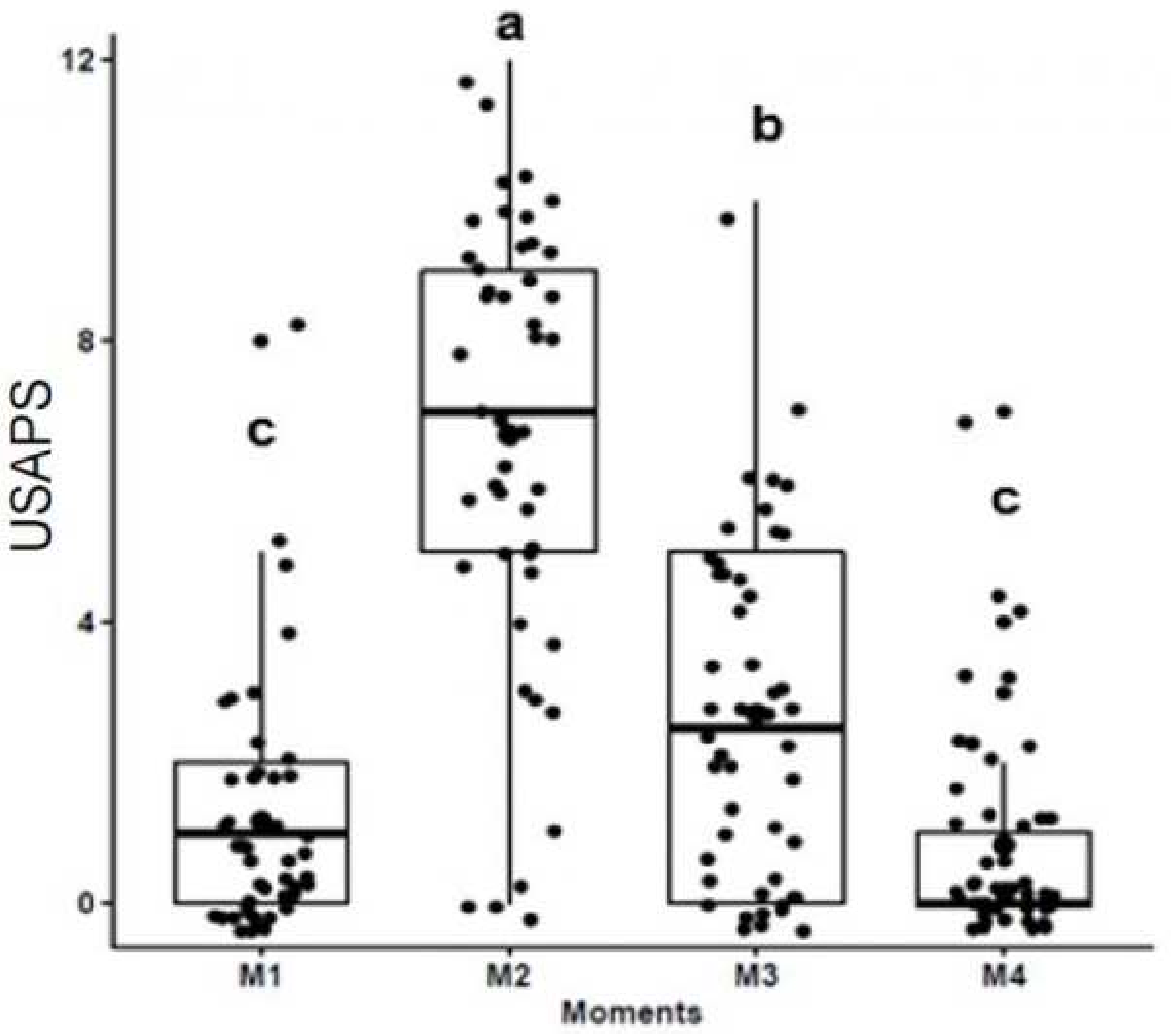

Fig 5

Click here to download Figure Fig. 5.tif

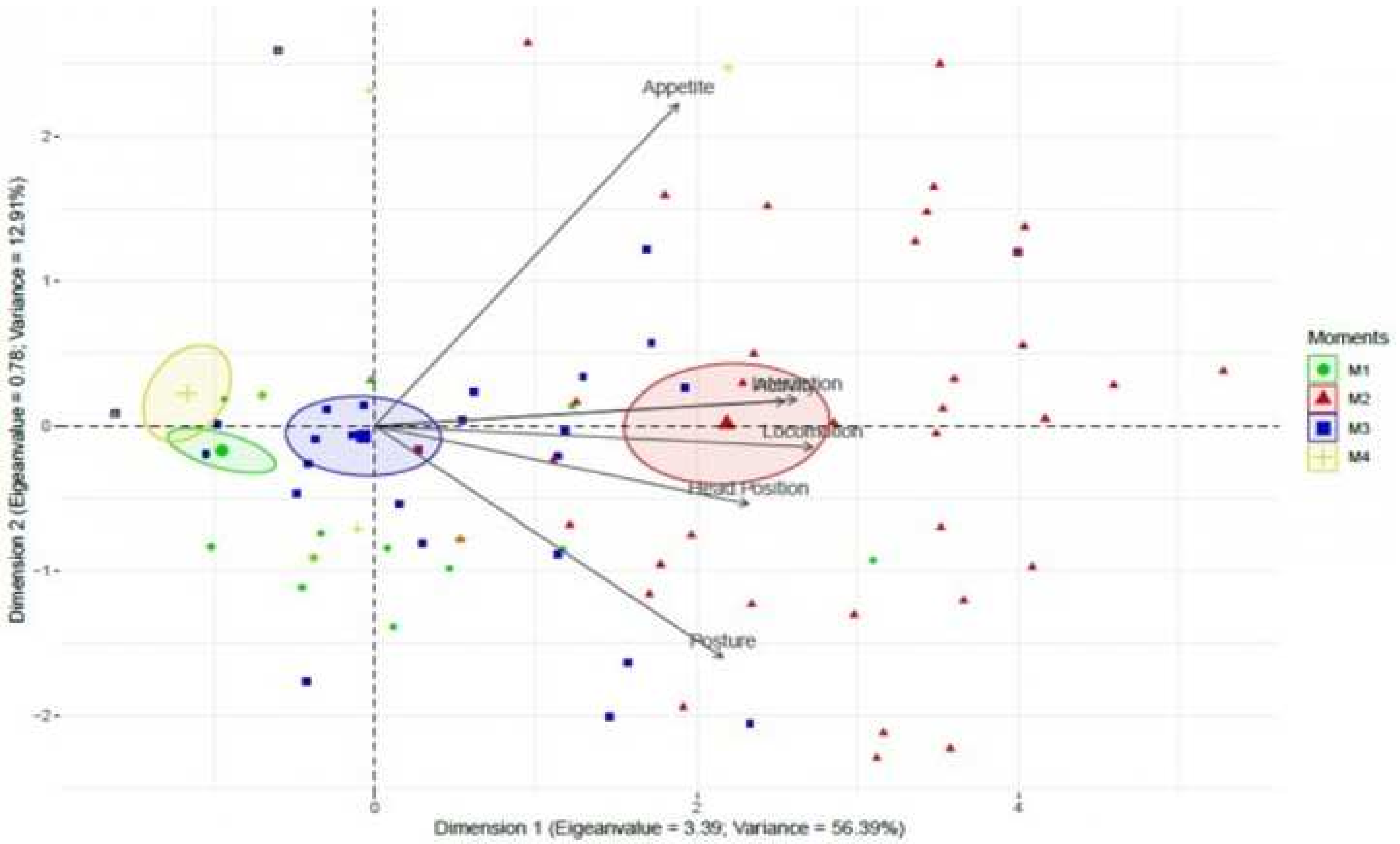

Fig 6

Click here to download Figure Fig 6.tif

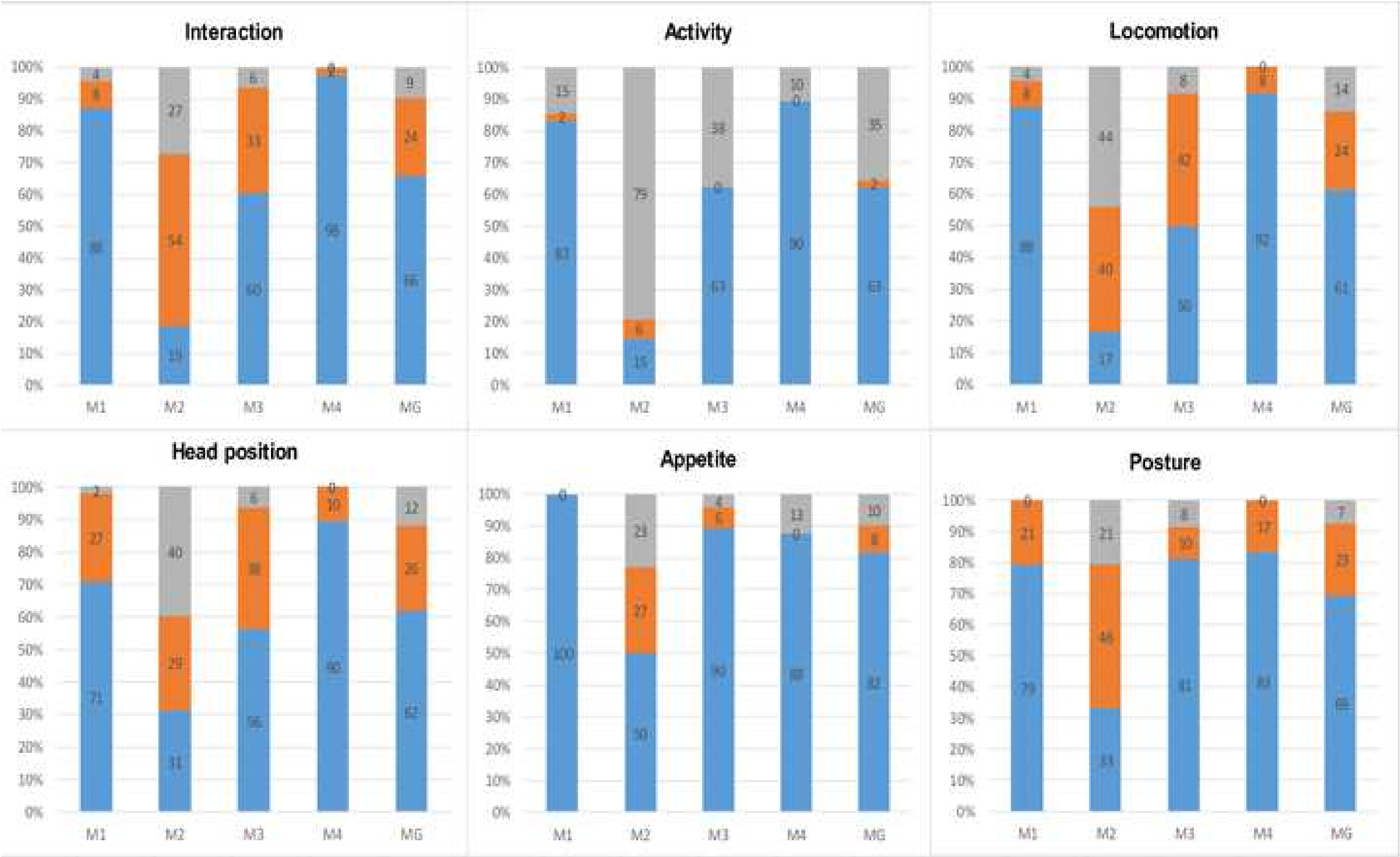

Click here to download Figure Fig 7.tif

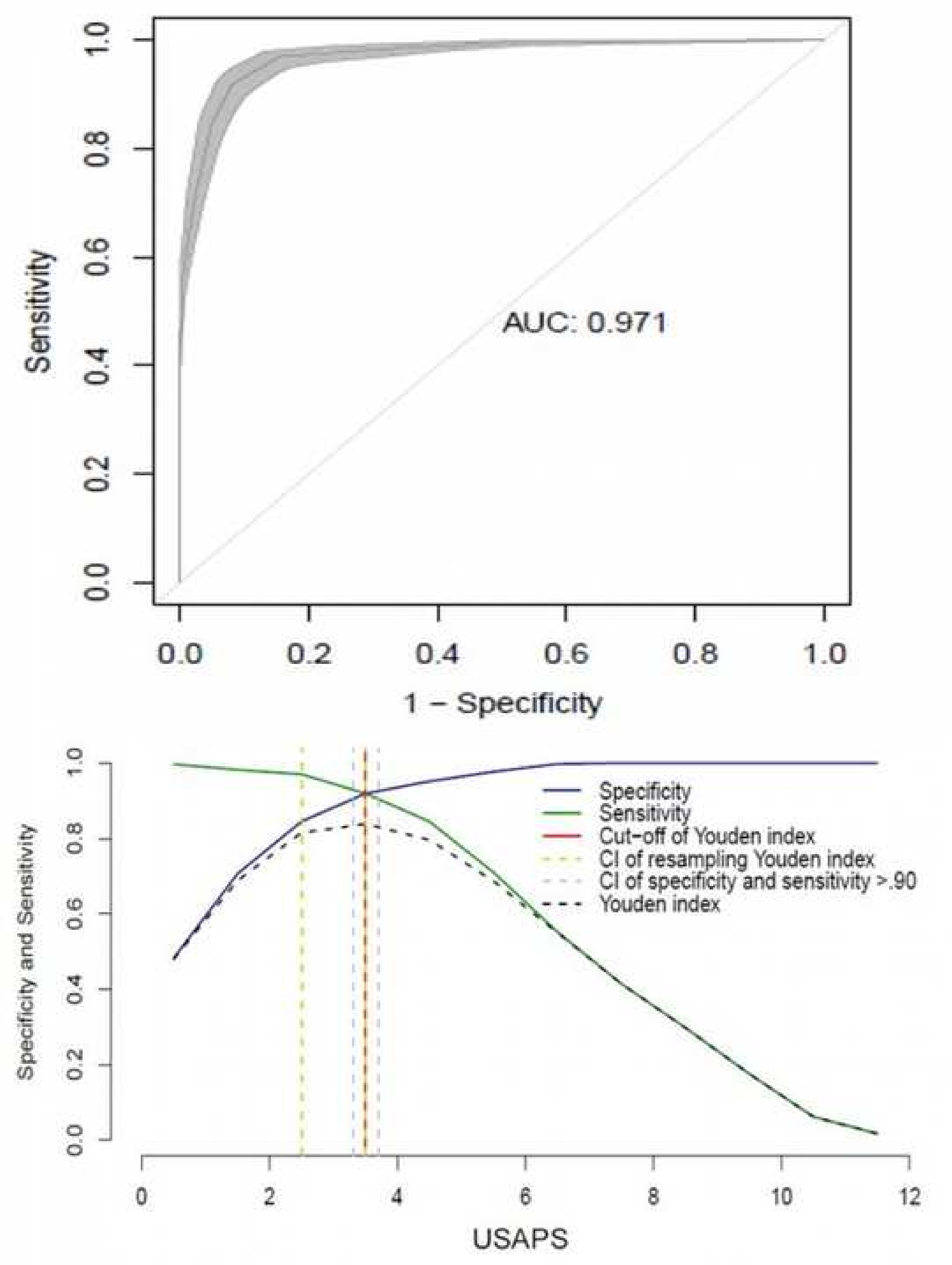

Click here to download Figure Fig 8.tif

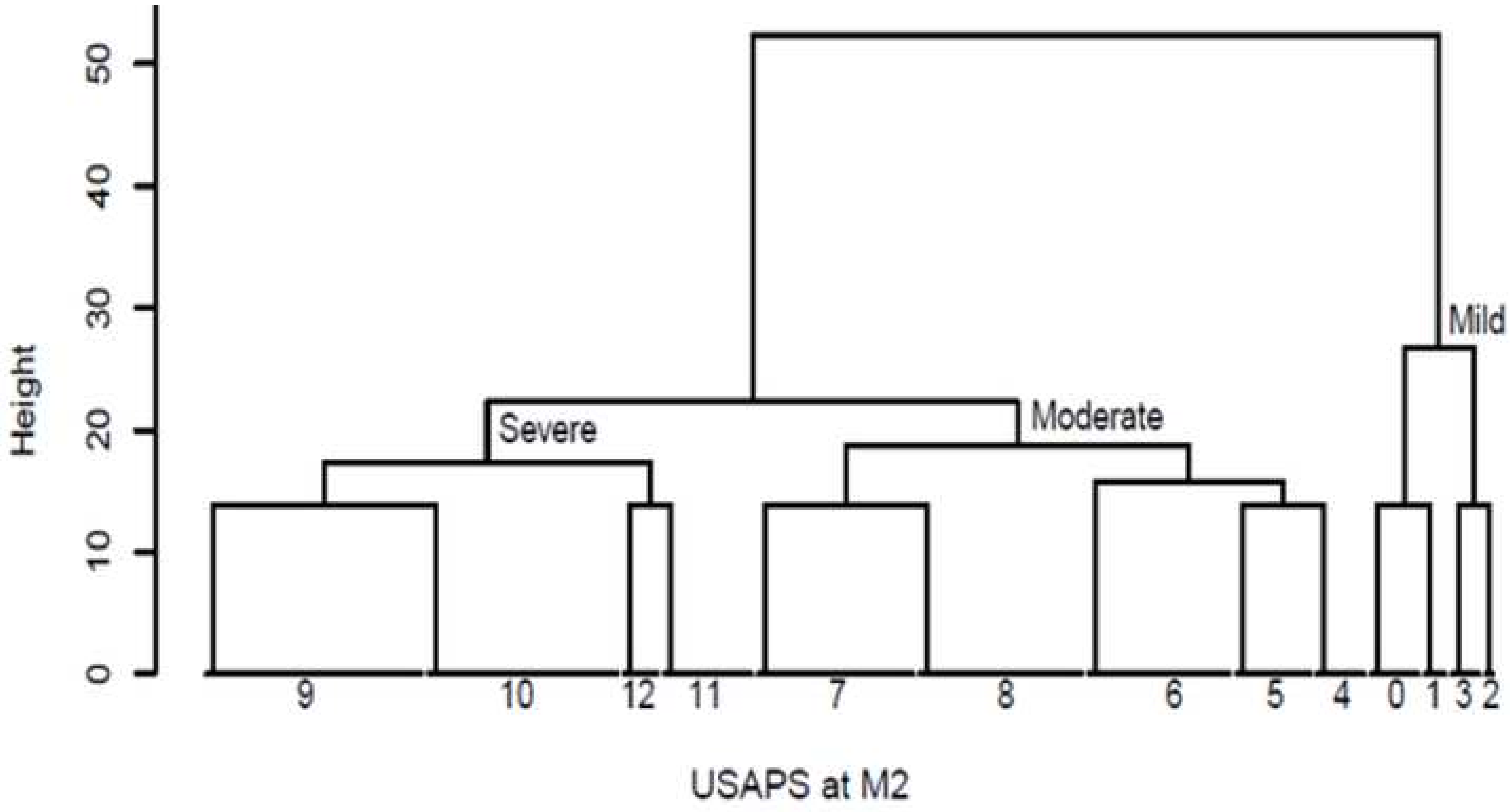

Fig 9 Click here to download Figure Fig 9.tif

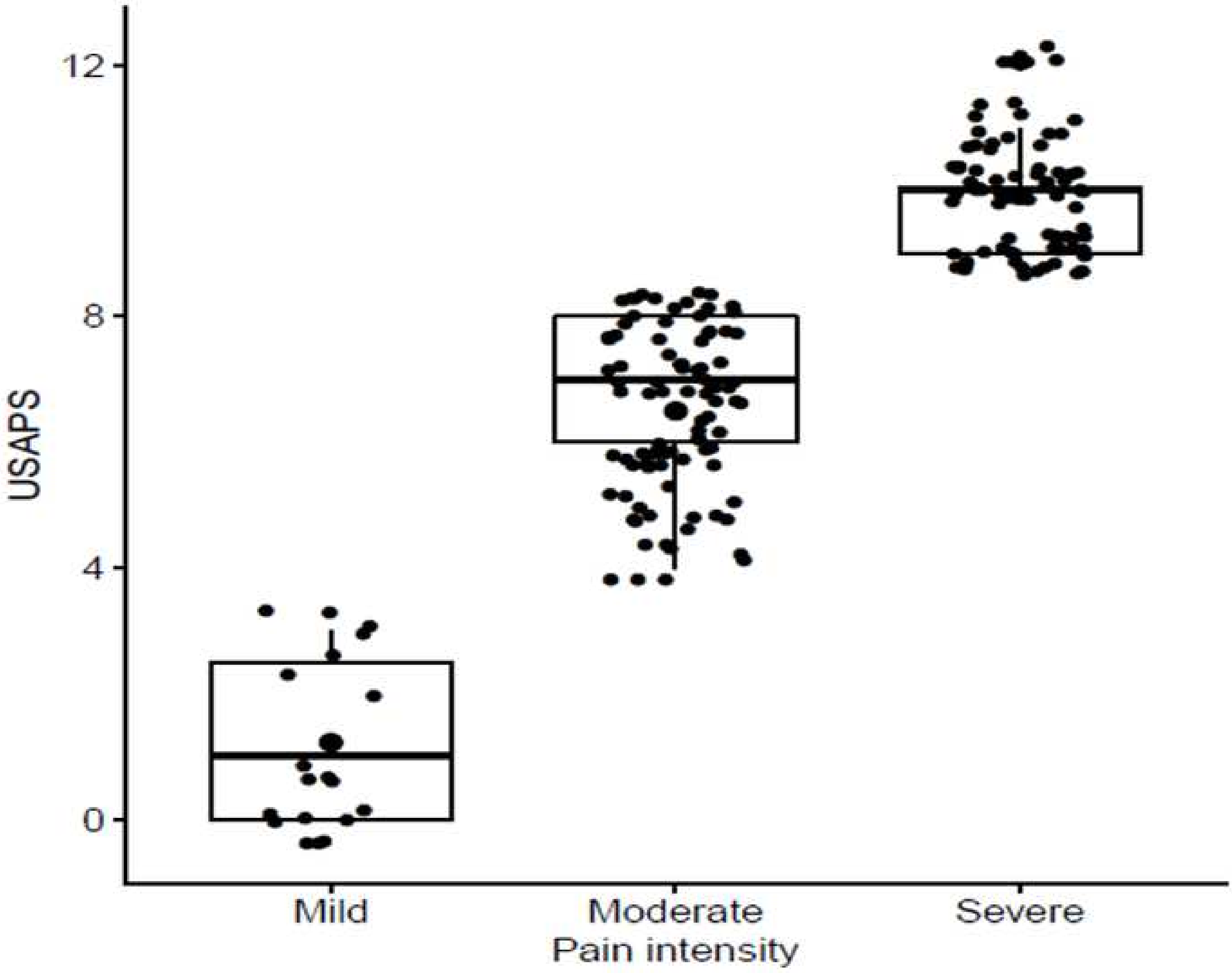

Click here to access/download

Supporting Information

S1 Table.pdf

Click here to access/download
Supporting Information
S2 Table.pdf

Click here to access/download
Supporting Information
S3 Table.pdf

Click here to access/download
Supporting Information
S4 Table.pdf
