# Supplementary material for: Validation of the Unesp-Botucatu composite scale to assess acute postoperative abdominal pain in sheep (USAPS)
Source: PLoS One. 2020 Oct 14;15(10):e0239622. doi: 10.1371/journal.pone.0239622 (PMC7556455; doi:10.1371/journal.pone.0239622)
Supplement: Attachment — Submitted filename: renamed_32687.pdf [file pone.0239622.s009.pdf]

Reviewer's comments – PONE-D-20-07213

General Comments

I would like to commend the authors who present a robust statistical study to validate an acute pain scale in sheep.

The only major concern I have within the manuscript is the attempt to classify the scores produced by the scale as mild, moderate and severe pain. While I understand appreciate in post-surgical studies, other groups often compare pain scores between groups, ultimately this is a non-continuous categorical scale. The section where mild-moderate-severe is defined reads as though the authors just divided the scale into three equal sections (0-3, 4-8, 9-12), and assumed each group.

This is in stark contrast to a previous section where the score 0 is defined as "without-pain". This within the manuscript, 0 is both no pain and mild pain. The majority of the introduction and discussion touts the robust statistical method employed in developing this scale. Something I think will be clear to the readership. This section certainly doesn't maintain the same high standards. The study design was meant to have non-painful and painful sheep, and develop a tool which could differentiate between these two states. The intervention point correlates with the sensitivity and specificity of a "score" to classify between the two. There was no stratification, nor any clear statistical method implored to stratify these sheep with any external method. I will point out some examples in specific sections later. But notably, in figure three, the highest score with the USAPS (12, maximum pain) is related to 5/10 on the NRS, 3/4 on the SDS, <50 on the VAS, and 5/12 on the facial scale. It does not seem that maximum pain with this scale correlates with the observers' "worst-possible pain" with the unidimensional instruments. Thus I would recommend the removal of this mild-moderate-severe classification. Further study would be required to determine any such classification.

Aside from this my other comments are minor.

I am not sure if it would be possible to shorten the introduction. While I understand the vast undertaking of the subject, I am not sure 71 references are necessary to justify the development of this scale.

Specific Comments

Page 1, Line 24 – "…is essential to diagnose pain and guarantee effective…"
No scale can <u>guarantee</u> analgesia. This term should be removed.

Page 3, Line 7 – "…and teaching, as their limitations to using these other species as models…"
Consider: "…and teaching, as there are limitations to using these other species as models…"

Page 3, Line 9 – "…studies on osteoporosis [17] and bone regeneration and osteointegration of dental implants [18]."
Consider: "…studies on osteoporosis [17], bone regeneration and osteointegration of dental implants [18]."

Page 3, Lines 11-15 – "Although there are several useful indicators to assess nociception in experimental situations, such as the injection of formalin into the interdigital space [19], von Frey filaments [20], tourniquets [21], electrical stimuli [22], and pneumo-mechanical stimulus in limbs [23], these are not reproducible and are difficult to use in clinical situations."
Are the authors trying to reference the instruments used to measure pain in references 19-23? Because the following are the
[reference] – pain model / measurement technique of each:

[19] – **Interdigital formalin injection** / Limb withdrawl and behavioural assessment
[20] – Peroneal nerve injury / **von Frey filament**
[21] – **Tourniquet** / Fractal HR
[22] – **Electrical stimuli** / EEG
[23] – **Pneumo-mechanical stimulus of the limbs** / Limb withdrawl and breath-to-breath CO_2

However the bold are what is reported in the manuscript. Please clarify what is meant as "indicator to assess nociception" and adjust accordingly.

Page 3, Line 15: "Actigraphy can be used to monitor…"
The authors should define actigraphy as it is not something the readership might be familiary with, nor is the term used in the reference provided.

Page 4, Line 12: "However, these instruments exclusively evaluate the intensity of pain dor…"
Consider removing the word "dor"
I am not sure this sentence also fully describes the advantage of multidimensional measurement over unidimensional instruments. Giving an example of how a tooth ache vs visceral pain cannot be simply compared might be another approach.

Page 4, Line 14: "…ethogram is produced to quantifies the duration…"
Consider: "…ethogram is produced to quantify the duration.."

Page 4, Line 23: "To guarantee the reliable measurement of pain…"
Again no scale can guarantee measurement. Consider: "To improve the reliability of pain measurement" or something similar.

Page 5, Lines 1-5: "Given the hypothesis that the scale proposed in the current study presents reliability, and content, construct and criterion validities, the main objective of this study was to validate a behavioral scale to assess acute pain in sheep undergoing soft tissue surgery

(laparoscopy), constructed from the literature and an ethogram, followed by refinement and subsequent validation, with definition of the cut-off point for analgesic intervention."
This is quite a long run-on sentence. Consider dividing it up for improved clarity.
Consider:
"The main objective of this study was to validate a behavioural scale to assess acute pain in sheep undergoing soft tissue surgery (laparoscopy). The authors constructed an ethogram from the literature, then used videos from this study for further refinement, and to define a cut-off point for analgesic intervention. The authors hypothesize that the final scale produced in the current study will be reliable and demonstrate content, construct and criterion validities."

Page 5, Lines 17-21: "The sheep were placed in stalls, close to the pen they lived in and where they were used to stay like a shelter when atmospheric conditions were extrem, 24 hours before the start of the study, during which they fasted for feed, and for 12 hours they fasted for water. In each stall (3x2x1.10mor2.20x2x1.20m-lengthxwidthxheight) 6 to 8 sheep or 2 to 4 sheep were housed, respectively."
These two sentences have a few typographical errors and lack clarity.
Consider:
"During the study period, sheep were housed in large (3 x 2 x 1.1m, length x width x height) and small (2.2 x 2 x 1.2m) pens with 6 to 8 or 2 to 4 animals each, respectively. The sheep were habituated to the pens for 24 hours before the start of the study."

Page 5, Line 23: "…position of the cameras and other adjustments, in order to guarantee the quality of filming.
Consider: "…positioning of the cameras and other adjustments, in order to optimize the quality of filming."

Page 6, Lines 6-7: "…and anesthetic infiltration with 2% lidocaine without vasoconstrictor (Xylestesin…"
The dose used for incisional block is missing.

Page 6, Lines 12-13: "In all animals, the same experienced surgeon performed a laparoscopy for follicular aspiration and replacement of follicular cells [73–75]…"
Reference 73 is for ovariectomy by laparotomy, partial video assisted ovariectomy and total laparascopic ovariectomy. Not sure the relevance here.

Page 6, Lines 18-19: "Fig 1."
Is this figure actually necessary? The order of events isn't very complicated.
Also this figure doesn't mention incisional lidocaine.

Page 6, Lines 22-23: "The procedures started at 9 am and the evaluations of the last animals ended around 7 pm."
Consider adding after this sentence that the 24 hour measurement occurred the next day.

Page 6, Line 24: "…and the mean temperature and humidity varied between…"

Was this the mean high? Or mean daily temperature?
Please add this descriptor.

Page 6, Line 26: “The presential observer made the recordings…”
“presential” is used multiple times in the manuscript, and should be removed.
Consider: The same observer (*author’s initials*) made the recordings…”

Page 7, Lines 1-2: “The camera was turned on and the presential researcher left the place, and stayed at least 10 m in order to minimize human interference in the behavior of the sheep.”
Consider: “The observer turned on the camera and then distanced themselves at least 10 m from the pens in order to minimize the effect of human presence on the behaviour of the sheep.”

Page 7, Line 22: “To elaborate the ethogram, the presential researcher evaluated…”
Consider: “For further elaboration of the ethogram, the same observer who recorded the videos evaluated….”

Page 8, Line 18: “…the presential observer…”
Remove the word presential.

Table 1:
Per my previous comment, I would recommend removing the determination of pain intensity. However if the authors justify its inclusion, the classification in this table is low, intermediate and high, not mild-moderate-severe

Page 13, Line 2: “A minimum sample size of 5 sheep [67] was estimated.”
This seems out of place. The 48 sheep reported in the methods should be stated here, and the sample size estimate should be in the methods.

Page 13, Line 23: “ranging from zero (without pain) to 12 (maximum pain).”
This is related to my previous comment.
This statement treats the USAPS as a unidimensional scale. I appreciate later that the values were treated as unidimensional for mathematical/statistical reasons, but this doesn’t seem appropriate.

Figure 6
There should be a definition of what blue/orange/grey means in the figure description

Page 23, Line 12: “10 The percentage of animals present in the diagnostic uncertainty zone (scores 3 and 4) was low at all times for all evaluators (11%; 9 - 15%). At M2, this percentage for all evaluators grouped was 7% (0 to 13%), which ensures that 93% of the sheep were detected as suffering pain with confidence at this moment (Table 13).”

Looking at the box plot in figure 4 , there are values less than 3. So 93% (100 minus 7%) of sheep were not detected as painful. Eyeballing there looks like there are 5 animals with a score of less than 3.
So the instrument detected 7% in a grey area and __% as painful and _% as non-painful. The 93% is the combined “clinically clear or useful”.

Page 24, Lines 1-20
Per my previous comment, I do not see the value of this section.
The authors have seemingly defined pain intensity as mild (0-3), moderate (4-8) and severe pain (9-12)
Then performed cluster analysis at M2
Based on the assumption from the study design, this is the painful phase, and “clinical experience” of the video evaluators suggests about 90% of these animals need rescue. But there was no stratification applied by any external measure. So the “intensities” here have simply been defined by the authors’ arbitrary cut-offs. Because, as mentioned, the “12’s” produced with this scale are not associated with the highest scores of the unidimensional scales.
The authors expected M4 to be worse than or equal to M3. Similar to the previous ruminant scale. Perhaps the model of pain used in this study doesn’t achieve extremes of intensities.
The ovariectomy reference 73 from the surgical description, using an NRS shows scores of 0.3 for video assisted or laparoscopic surgery (vs 5 for laparotomy). This scale may be sensitive to lower pain-intensities associated with minimally invasive surgeries. Future study, as the authors suggest later, is warranted.
Additionally: Figure 9’s description mentions a>b>c, but there are no letters in the figure.

Page 24, Line 27: “…as it can guarantee that sheep benefit from analgesia when necessary…”
Again, the scale cannot guarantee. This word should be changed. Even the authors recommend later in the discussion that clinical evaluation should still be considered for scores <4.

Page 26, Line 17: “…the trans-operative period.”
Do the authors mean peri-operative period? I am not sure what the trans-operative period is.

Page 26, Line 19: “Thus, vocalization is not, in the sheep species, an indicator of postoperative pain.”
Considering the great length, the authors go into in the introduction to talk about the limited availability of pain assessment in sheep in the literature. And that the references, as later discussed are mostly in lambs. The authors surely cannot claim this study as definitive proof that vocalization is not a component of pain, considering the current study only used one pain model.
This statement should be revised.

Page 26, Line 21: “A differential of the current study…”
I am not sure what is meant by differential?
Do the authors mean “A major difference in the current study compared to the literature…”?

Page 27, Line 23: "…it is premature to conclude about the dimension of the proposed pain…"
I am not sure "dimension" is what is meant here.
Is the comment meant to be about the universal application to other pain types?

Because the behavioural assessments in this instrument consider multiple dimensions, thus this would be a multidimensional scale, but it might just be applicable to abdominal pain.
For example in rats, a composite pain scale works for both laparotomy and visceral pain, however belly pressing which was a novel behaviour after laparotomy was not seen in the visceral models.

- Thomas et al. 2016. Efficacy of intrathecal morphine in a model of surgical pain in rats. PLOS One 11(10): e0163909
- Leung et al. 2019. Performance of behavioral assays: the Rat Grimace Scale, burrowing activity and a composite behavior score to identify visceral pain in an acute and chronic colitis model. Pain reports, 4(2) e718

Please clarify the intended comment here.

Page 28, Line 4: "As this is not the case in sheep…"
This statement, following the previous sentence, reads as though it is not necessary to compare the new instrument with a gold standard. Rather than the intended: because no gold standard exists, there is no standard to compare the current instrument with.
Consider removing this statement, and writing: "…considered the gold standard [71]. Since there is no validated scale with robust statistics to assess postoperative pain in sheep…"

Page 29, Line 9: "…Youden Index after surgery (M1)…rescue analgesia was indicated in 93% of sheep…"
This should be M2, which is the moment after surgery.
Per my previous comment, this wasn't 93% of sheep that needed rescue analgesia. The 7% are the sheep with scores 3-4, and 93% with scores <3 or >4. And there are (from figure 4) at least 5 with scores less than 3 (non-painful).

Page 29 Line 10-11: "…therefore the tool would foresee well that sheep were undergoing pain and then be treated, guaranteeing the animals' welfare.
The instrument isn't perfect, thus it cannot guarantee the animals treatment of pain, and also pain is only one component of welfare.
Consider: "…therefore the instrument is a

Page 29, Lines 17-21: "In this study, the differences observed in the pain scores between the moments, and especially at the expected moment of greatest pain compared to the other moments, confirm that the proposed scale is responsive both to identify intense degrees of pain, as well as moderate degrees, which occurred after rescue analgesia, or even mild pain, which occurred 24 hours after surgery."
Per my previous comment

- The authors expected M2>M4≥M3>M1 for pain scores, but actually observed M2>M3>M4>M1
- Thus the pain model does not provide the same order of scores as in previous studies in other species, or this instrument responds differently.

- Regardless, this order does not confer statistically robust stratification and cannot then be used to assume mild-moderate-severe pain classifications for a categorical scale.
- The design was not to achieve no pain → surgical pain → mild pain → moderate pain. This is impossible to predict. The authors developed a scale which differentiates between M1 and M2 (non-painful and painful) and then proves responsive to the treatment with rescue analgesia in M3. And then the pain had probably subsided due to the model by the 24h evaluation.

Also, with the given criteria, a score of 0/12 on the scale would be "mild pain". Though the authors frequently switch between mild and no-pain for 0/12.

Page 29, Line 28: "…apparently the instrument can be used in different sheep breeds."
Considering only 3 breeds, all of which are dairy sheep from the same geographical region, were assessed. It is rather presumptuous to state the scale can be used on all sheep breeds. The authors can postulate this theory, but should recommend further study before making such a definitive statement.

Page 32, Line 3-7: "To our knowledge, the scales that assess acute pain in various animal species do not classify pain intensity based on their scores, except in an empirical way [44]. In this study, the zone of diagnostic uncertainty (3 - 4) corresponded to the lower limit of moderate pain scores (4), insuring that sheep suffering from moderate pain would be treated according to the cut-off point."
Per my previous comment
I would imagine other scales were not classified in this manner because they are also non-continuous categorical scales used to assess whether or not pain is present.

This paragraph also highlights the completely arbitrary definition of "moderate pain"
WSAVA Guidelines for the recognition, assessment, and treatment of pain describes the perceived pain of ovariohysterectomy in dogs and cats as "Moderate".
I fully appreciate this is perceptions, and species differences can be vast. However:
The same references used in the surgical description (73) states mean pain score of ovariectomy by laparotomy as 5.6 (on a 10-pt scale, 0-9) and 0.3 out of 9 for both video-assisted and pure-laparascopic ovariectomy.
Additionally, suggesting that this pain scale recommends that the readership should not treat "mild pain" (scores 0-3) takes away from the robust statistical approach of all the other steps in this instruments development.
I do not understand what this classification achieves.

Page 32, Line 17: "…video analysis does not necessarily equate to presential analysis."
Consider: "…video analysis does not necessarily equate to in-person real-time analysis."

Page 32, Line 22: "According to a study by…"
Consider: "According to studies by our group…" since multiple references are used.
