# Supplementary material for: Validation of the Unesp-Botucatu composite scale to assess acute postoperative abdominal pain in sheep (USAPS)
Source: PLoS One. 2020 Oct 14;15(10):e0239622. doi: 10.1371/journal.pone.0239622 (PMC7556455; doi:10.1371/journal.pone.0239622)
Supplement: Attachment — Submitted filename: Response to reviewer 2.docx [file pone.0239622.s010.docx]

**Manuscript PONE-D-20-07213:**

**Validation of the Unesp-Botucatu composite scale to assess acute postoperative abdominal pain in sheep (USAPS)**

Dear Editor

Thank you very much for the comments regarding this paper. All corrections have been performed according to both Reviewers and each comment responded to separately.

We hope that after these corrections you consider the manuscript suitable for publication, but we are happy to answer any further questions.

Yours sincerely,

The authors

**REVIEWER: 2**

Dear Reviewer:

Thank you very much for the comments regarding this paper. All corrections have been performed according to your suggestions and each comment responded to separately.

We hope that after these corrections you consider the manuscript suitable for publication, but we are happy to answer any further questions.

Yours sincerely,

The authors

Recommendation:

Comments to the Author

Do you want your identity to be public for this peer review? For information about this choice, including consent withdrawal, please see our [Privacy Policy](https://www.plos.org/privacy-policy).

Reviewer #2: Yes:

Comments to the Author

1. Is the manuscript technically sound, and do the data support the conclusions?

Reviewer #2: Yes

2. Has the statistical analysis been performed appropriately and rigorously?

Reviewer #2: Yes

3. Have the authors made all data underlying the findings in their manuscript fully available?

Reviewer #2: Yes

4. Is the manuscript presented in an intelligible fashion and written in standard English?

Reviewer #2: Yes

-----

5. Review Comments to the Author

Please use the space provided to explain your answers to the questions above.

Reviewer #2: Please see the attached review document as formatting will assist in interpretation of the comments.

Here are the general comments:

I would like to commend the authors who present a robust statistical study to validate an acute pain scale in sheep.

Answer: The authors appreciate the time taken to review this manuscript and for including your suggestions that have been fully considered unless otherwise stated.

The statistical analysis has been performed again according to Reviewer 1 (data from all observers and phases 1 and 2 were combined instead of using data from phase 2 of the gold standard observer). Although small changes were observed in the Tables presented in the results section, the results are very similar to the previous submission. All changes are highlighted in yellow.

The only major concern I have within the manuscript is the attempt to classify the scores produced by the scale as mild, moderate and severe pain. While I understand appreciate in post-surgical studies, other groups often compare pain scores between groups, ultimately this is a non-continuous categorical scale. The section where mild-moderate-severe is defined reads as though the authors just divided the scale into three equal sections (0-3, 4-8, 9-12), and assumed eachgroup.

This is in stark contrast to a previous section where the score 0 is defined as “without-pain”. This within the manuscript, 0 is both no pain and mild pain. The majority of the introduction and discussion touts the robust statistical method employed in developing this scale. Something I think will be clear to the readership.

This section certainly doesn’t maintain the same high standards. The study design was meant to have non-painful and painful sheep, and develop a tool which could differentiate between these two states.

The only major concern I have within the manuscript is the attempt to classify the scores produced by the scale as mild, moderate and severe pain. While I understand appreciate in post-surgical studies, other groups often compare pain scores between groups, ultimately this is a non-continuous categorical scale. The section where mild-moderate-severe is defined reads as though the authors just divided the scale into three equal sections (0-3, 4-8, 9-12), and assumed each group.

This is in stark contrast to a previous section where the score 0 is defined as “without-pain”. This within the manuscript, 0 is both no pain and mild pain. The majority of the introduction and discussion touts the robust statistical method employed in developing this scale. Something I think will be clear to the readership.

This section certainly doesn’t maintain the same high standards. The study design was meant to have non-painful and painful sheep, and develop a tool which could differentiate between these two states.

Answer: The authors thank you for pointing this out. Two consultant statisticians were hired, and a new method used to present this analysis appropriately. We hope that this analysis now fulfils your expectation, otherwise please let us know.

In this study it was expected that sheep would not suffer pain at the preoperative point (M1), followed by a maximum expression of pain at the immediate postoperative moment (M2), reduction in pain after analgesia (M3), and little pain 24h after surgery (M4). We consider that the instrument for assessing pain does not only contemplate the diagnosis of pain but also quantifies pain intensity. Therefore, we used the simple descriptive scale (SDS) as a reference tool for pain intensity because SDS describes four levels of pain (1 = no pain; 2 = mild pain; 3 = moderate pain; and 4 = severe pain). A multivariate statistical analysis was applied to explore correspondences between categorical variables to classify the total score of USAPS in each of the SDS pain intensities. After consulting two statisticians, correspondences were calculated by two methods: (1) visual judgment of the proximity of the USAPS and SDS levels in the perceptual map by multiple correspondence analysis (MCA); and (2) value of the standardized chi-square residue. First, both scales were submitted to MCA which uses the basic concept of chi-square to standardize the frequencies of the variables and to form a basis for correspondences (interrelationship) between categorical variables (Greenacre, 2010). In addition, to make the USAPS classification more accurate, the correspondences between the scale levels by the chi-square residue were used to group the USAPS levels among each level of pain intensity according to the SDS.

To confirm a significant difference between the USAPS intensities according to MCA, the Scott Knott test was applied. This test uses a “likelihood” relation to judge the difference between clusters. In view of this new approach, we understand that the classification of USAPS intensities was carried out by robust and appropriate mathematical methods, already applied in other areas to group and classify categorical variables.

Salas, Y., Márquez, A., Diaz, D., & Romero, L. (2015). Epidemiological study of mammary tumors in female dogs diagnosed during the period 2002-2012: a growing animal health problem. PloS one, 10(5).

Wilfart, A., Espagnol, S., Dauguet, S., Tailleur, A., Gac, A., & Garcia-Launay, F. (2016). ECOALIM: A dataset of environmental impacts of feed ingredients used in French animal production. PloS one, 11(12).

Guinet, F., Avé, P., Jones, L., Huerre, M., &Carniel, E. (2008). Defective innate cell response and lymph node infiltration specify Yersinia pestis infection. PloS one, 3(2).

Le Rumeur, E., Carre, F., Bernard, A. M., Bansard, J. Y., Rochcongar, P., & De Certaines, J. D. (1994). Multiparametric classification of muscle T 1, and T 2 relaxation times determined by magnetic resonance imaging. The effects of dynamic exercise in trained and untrained subjects. The British journal of radiology, 67(794), 150-156.

The intervention point correlates with the sensitivity and specificity of a “score” to classify between the two. There was no stratification, nor any clear statistical method implored to stratify these sheep with any external method. I will point out some examples in specific sections later.

Answer: The stratification of the sheep with and without pain was not performed before the statistical analysis to estimate pain intensity for two reasons. Stratification based on the assessment time points could be ambiguous, because even in the immediate postoperative period (M2) some sheep did not appear to be suffering pain, possibly due to the individuality of the pain sensation. Besides this, the stratification by the cut-off point was not performed because by using this new mathematical approach sheep without pain (1 = no pain) and with pain (2 = mild pain; 3 = moderate pain; and 4 = severe pain) were included in the SDS pain intensities and classified below or above the cut-off point of both scales (≤ 3for USAPS and ≤1 for SDS), which shows consistency with the mathematical method used for the classification of pain and with the observers' scores; as well as which the intensity "no pain" is relevant and must be computed. This was the reason the statistical analysis was performed with all-time points and all observers to define the intensity of the pain*.*

But notably, in figure three, the highest score with the USAPS (12, maximum pain) is related to 5/10 on the NRS, 3/4 on the SDS, <50 on the VAS, and 5/12 on the facial scale. It does not seem that maximum pain with this scale correlates with the observers’ “worst-possible pain” with the unidimensional instruments. Thus I would recommend the removal of this mild-moderate-severe classification. Further study would be required to determine any such classification.

Answer: Unidimensional scales (NRS, SDS, and VAS) depend on the expertise of the evaluator and are considered more subjective when compared to composite and multidimensional scales. Because composite scales have well-defined descriptors, they suffer less influence of observer's interpretation and are usually more reliable. However, all these scales follow the same logic in which the highest numerical score represents the highest level of pain. Therefore, it is expected that the scores of unidimensional scales are not identical to USAPS, but are sufficiently correlated with the USAPS (rho> 0.81).

We recognize that this approach has limitations. Our group published a scale in pigs using the same approach (Luna et al 2020. Validation of the UNESP-Botucatu pig composite acute pain scale (UPAPS). PLoS ONE, 15(6), e0233552) and we hope to improve the quantification of pain in the future, but it is the best we can do currently according to the statisticians we consulted. If after this new analysis the reviewer is not satisfied, the authors are happy to remove this analysis of pain intensities.

Aside from this, my other comments are minor.

I am not sure if it would be possible to shorten the introduction. While I understand the vast undertaking of the subject, I am not sure 71 references are necessary to justify the development of this scale.

Answer: We have done our best to shorten the manuscript without leaving out necessary information. The number of references in the introduction has been reduced by almost half and the manuscript has been reviewed by a native English speaker to improve fluency.

*Specific Comments*

Page 1, Line 24 – “…is essential to diagnose pain and guarantee effective…”

No scale can guarantee analgesia. This term should be removed.

Answer: Corrected to “is essential to diagnose pain and improve decision making for analgesia” (page 1, lines 22-23).

Page 3, Line 7 – “…and teaching, as their limitations to using these other species as models…”

Consider: “…and teaching, as there are limitations to using these other species as models…”

Answer: This has been deleted to reduce the size of the introduction.

Page 3, Line 9 – “…studies on osteoporosis [17] and bone regeneration and osteointegration of dental implants [18].”

Consider: “…studies on osteoporosis [17], bone regeneration and osteointegration of dental implants [18].”

Answer: This has been deleted to reduce the size of the introduction.

Page 3, Lines 11-15 – “Although there are several useful indicators to assess nociception in experimental situations, such as the injection of formalin into the interdigital space [19], von Frey filaments [20], tourniquets [21], electrical stimuli [22], and pneumo-mechanical stimulus in limbs [23], these are not reproducible and are difficult to use in clinical situations.”

Are the authors trying to reference the instruments used to measure pain in references 19-23?

Because the following are the

[reference] – pain model / measurement technique of each:

[19] – **Interdigital formalin injection** / Limb withdrawl and behaviouralassessment
[20] – Peroneal nerve injury / **von Freyfilament**
[21] – **Tourniquet** / Fractal HR
[22] – **Electrical stimuli** /EEG
[23] – **Pneumo-mechanical stimulus of the limbs** / Limb withdrawl and breath-to-breathCO2

However the bold are what is reported in the manuscript. Please clarify what is meant as “indicator to assess nociception” and adjust accordingly.

I swapped von frey filament for the **pain model** to make everything coherent: such as interdigital formalin injection [19], **peroneal nerve injury**[20], tourniquets [21], electrical stimuli [22], and pneumo-mechanical stimulus of the limbs [23],

Answer: The paragraph has been shortened to reduce the size of the introduction and the references have been updated “Although there are several experimental methods to assess nociception [10-14]” – see page 3, line 1.

Page 3, Line 15: “Actigraphy can be used to monitor…”

The authors should define actigraphy as it is not something the readership might be familiary with, nor is the term used in the reference provided.

Answer: Corrected to “Actigraphy can be used to monitor the sheep activity” (page 3, lines 2-3).

Page 4, Line 12: “However, these instruments exclusively evaluate the intensity of pain dor…”

Consider removing the word “dor”

Answer: Corrected (page 3, line 26).

I am not sure this sentence also fully describes the advantage of multidimensional measurement over unidimensional instruments. Giving an example of how a tooth ache vs visceral pain cannot be simply compared might be another approach.

Answer: Corrected to (in bold) “However, these instruments exclusively evaluate the intensity of pain, whereas multidimensional or composite scales include sensory, motor, and emotional qualities and **may be developed to differentiate specific types of pain**” – page 3, lines 27-28.

Page 4, Line 14: “…ethogram is produced to quantifies the duration…”

Consider: “…ethogram is produced to quantify the duration..”

Answer: Corrected (page 4, lines 1).

Page 4, Line 23: “To guarantee the reliable measurement of pain…”

Again no scale can guarantee measurement. Consider: “To improve the reliability of pain measurement” or something similar.

Answer: Corrected as suggested (page, 4, line 9-10).

Page 5, Lines 1-5: “Given the hypothesis that the scale proposed in the current study presents reliability, and content, construct and criterion validities, the main objective of this study was to validate a behavioral scale to assess acute pain in sheep undergoing soft tissue surgery(laparoscopy), constructed from the literature and an ethogram, followed by refinement and subsequent validation, with definition of the cut-off point for analgesic intervention.”

This is quite a long run-on sentence. Consider dividing it up for improved clarity. Consider: “The main objective of this study was to validate a behavioural scale to assess acute pain in sheep undergoing soft tissue surgery (laparoscopy). The authors constructed an ethogram from the literature, then used videos from this study for further refinement, and to define a cut-off point for analgesic intervention. The authors hypothesize that the final scale produced in the current study will be reliable and demonstrate content, construct and criterion validities.”

Answer: Corrected as suggested (page 4, lines 19-24).

Page 5, Lines 17-21: “The sheep were placed in stalls, close to the pen they lived in and where they were used to stay like a shelter when atmospheric conditions were extrem, 24 hours before the start of the study, during which they fasted for feed, and for 12 hours they fasted for water. In each stall (3x2x1.10mor2.20x2x1.20m-lengthxwidthxheight) 6 to 8 sheep or 2 to 4 sheep were housed, respectively.”

These two sentences have a few typographical errors and lack clarity.

Consider: “During the study period, sheep were housed in large (3 x 2 x 1.1m, length x width x height) and small (2.2 x 2 x 1.2m) pens with 6 to 8 or 2 to 4 animals each, respectively. The sheep were habituated to the pens for 24 hours before the start of the study.”

Answer: Corrected as suggested (page 5, lines 6-10).

Page 5, Line 23: “…position of the cameras and other adjustments, in order to guarantee the quality of filming.

Consider: “…positioning of the cameras and other adjustments, in order to optimize the quality of filming.”

Answer: Corrected as suggested (page 5, line 1-2). The other reviewer also asked for inclusion of the camera model.

Page 6, Lines 6-7: “…and anesthetic infiltration with 2% lidocaine without vasoconstrictor (Xylestesin…”

The dose used for incisional block is missing.

Answer: The information has been included (page 5, lines 19-20).

Page 6, Lines 12-13: “In all animals, the same experienced surgeon performed a laparoscopy for follicular aspiration and replacement of follicular cells [73–75]…”

Reference 73 is for ovariectomy by laparotomy, partial video assisted ovariectomy and total laparascopic ovariectomy. Not sure the relevance here.

Answer: Only one reference has been left here as it was used later as well (page 5, line 26).

Page 6, Lines 18-19: “Fig 1.”

Is this figure actually necessary? The order of events isn’t very complicated.

Answer: This has been maintained to add clarity for the other reviewer (page 6, line 16-17).

Also this figure doesn’t mention incisional lidocaine.

Answer: Included (page 6, Fig 1)

Page 6, Lines 22-23: “The procedures started at 9 am and the evaluations of the last animals ended around 7 pm.”

Consider adding after this sentence that the 24 hour measurement occurred the next day.

Answer: Included (page 6, lines 6-7).

Page 6, Line 24: “…and the mean temperature and humidity varied between…”

Was this the mean high? Or mean daily temperature? Please add this descriptor.

Answer: Corrected to mean daily temperature,

Page 6, Line 26: “The presential observer made the recordings…”

“presential” is used multiple times in the manuscript, and should be removed.

Consider: The same observer (author’s initials) made the recordings…”

Answer: Corrected to “the in-person observer (NEOFS) made the recordings…” (page 6, line 19).

Page 7, Lines 1-2: "The camera was turned on and the presential researcher left the place and stayed at least 10 m in order to minimize human interference in the behavior of the sheep."

Consider: “The observer turned on the camera and then distanced themselves at least 10 m from the pens in order to minimize the effect of human presence on the behaviour Answer: Corrected according to the Reviewer´s suggestion (page 6, line 20-22).

Page 7, Line 22: “To elaborate the ethogram, the presential researcher evaluated…” Consider: “For further elaboration of the ethogram, the same observer who recorded the videos evaluated….”

Answer: This paragraph has been reformulated to better explain the elaboration of the ethogram to the other reviewer (page 7, lines 15-21).

Page 8, Line 18: “…the presential observer…”

Remove the word presential.

Answer: Corrected (page 7, line 15).

Table 1:

Per my previous comment, I would recommend removing the determination of pain intensity. However if the authors justify its inclusion, the classification in this table is low, intermediate and high, not mild-moderate-severe

Answer: According to the new analysis the classification has been maintained as described by the simple descriptive scale, because SDS was the reference to quantify the intensity of pain by the USAPS. However, the authors are happy to change this if the reviewer is not satisfied (page 9, Table 1).

Page 13, Line 2: “A minimum sample size of 5 sheep [67] was estimated.”

This seems out of place. The 48 sheep reported in the methods should be stated here, and the sample size estimate should be in the methods.

Answer: The sample size calculation has been removed as we had no previous data to estimate sample size.

Page 13, Line 23: “ranging from zero (without pain) to 12 (maximum pain).”

This is related to my previous comment. This statement treats the USAPS as a unidimensional scale. I appreciate later that the values were treated as unidimensional for mathematical/statistical reasons, but this doesn’t seem appropriate.

Answer: A paragraph in the discussion raises the debate about the mathematical and biological dimensions (page 27, lines 14-23). The authors agree that the scale was multidimensional in biological terms.

Figure 6

There should be a definition of what blue/orange/grey means in the figure description

Answer: Included in the legend (new figure 3 - page 16, line 8).

Page 23, Line 12: “10 The percentage of animals present in the diagnostic uncertainty zone (scores 3 and 4) was low at all times for all evaluators (11%; 9 - 15%). At M2, this percentage for all evaluators grouped was 7% (0 to 13%), which ensures that 93% of the sheep were detected as suffering pain with confidence at this moment (Table 13).”

Looking at the box plot in figure 4, there are values less than 3. So 93% (100 minus 7%) of sheep were not detected as painful. Eyeballing there looks like there are 5 animals with a score of less than 3.

So the instrument detected 7% in a grey area and% as painful and _% as non-painful. The 93% is the combined “clinically clear or useful”.

Answer: Please accept our apologies for this mistake and lack of attention. We are grateful to the reviewer for pointing this out. This sentence has been deleted: “At M2 95% (92-99) of the sheep were detected as suffering pain with confidence at this moment” (Table 11).

Page 24, Lines 1-20

Per my previous comment, I do not see the value of this section.

The authors have seemingly defined pain intensity as mild (0-3), moderate (4-8) and severe pain (9-12). Then performed cluster analysis at M2

Answer: Please check previous comments about this analysis. Data from all time-points are now used. If the reviewer is not satisfied with the new more robust statistical analysis, the authors are happy to remove this section from the methods, results, and discussion.

Based on the assumption from the study design, this is the painful phase, and “clinical experience” of the video evaluators suggests about 90% of these animals need rescue. But there was no stratification applied by any external measure. So, the “intensities” here have simply been defined by the authors’ arbitrary cut-offs. Because, as mentioned, the “12’s” produced with this scale are not associated with the highest scores of the unidimensional scales.

Answer: Please see the previous comment about the same subject.

The authors expected M4 to be worse than or equal to M3. Similar to the previous ruminant scale. Perhaps the model of pain used in this study doesn’t achieve extremes of intensities. The ovariectomy reference #73 from the surgical description, using an NRS shows scores of 0.3 for video assisted or laparoscopic surgery (vs 5 for laparotomy). This scale may be sensitive to lower pain-intensities associated with minimally invasive surgeries. Future study, as the authors suggest later, is warranted.

Answer: Thank you for this comment. The authors are currently performing a study with orthopaedic surgery to clinically validate (or not) this scale.

Additionally: Figure 9’s description mentions a>b>c, but there are no letters in the figure. Answer: Corrected, the letters have been included (page 23, Fig. 9).

Page 24, Line 27: “…as it can guarantee that sheep benefit from analgesia when necessary…” Again, the scale cannot guarantee. This word should be changed. Even the authors recommend later in the discussion that clinical evaluation should still be considered for scores <4.

Answer: Corrected (page 24, lines 3).

Page 26, Line 17: “…the trans-operative period.”

Do the authors mean peri-operative period? I am not sure what the trans-operative period is.

Answer: This has been deleted because it was meant to say that restraint in the preoperative moment may lead to vocalization (page 25, line 20).

Page 26, Line 19: “Thus, vocalization is not, in the sheep species, an indicator of postoperative pain.”

Considering the great length, the authors go into in the introduction to talk about the limited availability of pain assessment in sheep in the literature. And that the references, as later discussed are mostly in lambs. The authors surely cannot claim this study as definitive proof that vocalization is not a component of pain, considering the current study only used one pain model.

This statement should be revised.

Answer: Corrected to “Thus vocalization is not an indicator of postoperative pain in adult sheep submitted to laparoscopy, as reported previously in sheep undergoing severe painful stimuli” (page 25, lines 21-22).

Page 26, Line 21: “A differential of the current study…”

I am not sure what is meant by differential? Do the authors mean “A major difference in the current study compared to the literature…”?

Answer: Corrected (page 26, line 24).

Page 27, Line 23: “…it is premature to conclude about the dimension of the proposed pain…” I am not sure “dimension” is what is meant here.

Is the comment meant to be about the universal application to other pain types?

Answer: This was focused only on the mathematical approach and corrected to differentiate the mathematical and biological approaches (see page 3, lines 25-28). The last sentence of this paragraph has been changed to exclude the relationship between dimensions and types of pain (page 27, lines 22-23).

Page 28, Line 4: “As this is not the case in sheep…”

This statement, following the previous sentence, reads as though it is not necessary to compare the new instrument with a gold standard. Rather than the intended: because no gold standard exists, there is no standard to compare the current instrument with.

Consider removing this statement, and writing: “…considered the gold standard [71]. Since there is no validated scale with robust statistics to assess postoperative pain in sheep…”

Answer: The statistical analysis has been performed again as suggested by the other reviewer. As data from all observers were grouped there are no longer comparisons against the gold standard observer. The paragraph has been rephrased as suggested by the other reviewer.

Page 29, Line 9: “…Youden Index after surgery (M1) rescue analgesia was indicated in 93% of sheep…”

This should be M2, which is the moment after surgery.

Answer: Corrected (page 31, lines 21-22).

Per my previous comment, this wasn’t 93% of sheep that needed rescue analgesia. The 7% are the sheep with scores 3-4, and 93% with scores <3 or >4. And there are (from figure 4) at least 5 with scores less than 3 (non-painful).

Answer: Please see the previous comment about the same subject.

Page 29 Line 10-11: “…therefore the tool would foresee well that sheep were undergoing pain and then be treated, guaranteeing the animals’ welfare.

The instrument isn’t perfect, thus it cannot guarantee the animals treatment of pain, and also pain is only one component of welfare.

Consider: “…therefore the instrument is a

Answer: Corrected (page 31, lines 21-24).

Page 29, Lines 17-21: “In this study, the differences observed in the pain scores between the moments, and especially at the expected moment of greatest pain compared to the other moments, confirm that the proposed scale is responsive both to identify intense degrees of pain, as well as moderate degrees, which occurred after rescue analgesia, or even mild pain, which occurred 24 hours after surgery.”

Per my previous comment

- The authors expected M2>M4≥M3>M1 for pain scores, but actually observed M2>M3>M4>M1
- Thus the pain model does not provide the same order of scores as in previous studies in other species, or this instrument respondsdifferently.

Answer: This part has been amended (page 29, lines 6-9).

- Regardless, this order does not confer statistically robust stratification and cannot then be used to assume mild-moderate-severe pain classifications for a categoricalscale.
- The design was not to achieve no pain → surgical pain → mild pain → moderate pain. This is impossible to predict. The authors developed a scale which differentiates between M1 and M2 (non-painful and painful) and then proves responsive to the treatment with rescue analgesia in M3. And then the pain had probably subsided due to the model by the 24hevaluation.
- Also, with the given criteria, a score of 0/12 on the scale would be “mild pain”. Though the authors frequently switch between mild and no-pain for 0/12.

Answer: Please see the previous comment about the same subject. The manuscript has been reformulated and so has this analysis. If after this new analysis the reviewer is not satisfied, the authors are happy to remove this analysis of pain intensities

Page 29, Line 28: “…apparently the instrument can be used in different sheep breeds.” Considering only 3 breeds, all of which are dairy sheep from the same geographical region, were assessed. It is rather presumptuous to state the scale can be used on all sheep breeds. The authors can postulate this theory, but should recommend further study before making such a definitive statement.

Answer: We agree with the reviewer. However, with the new statistical analysis, there was a difference between species, so we have excluded this sentence.

Page 32, Line 3-7: “To our knowledge, the scales that assess acute pain in various animal species do not classify pain intensity based on their scores, except in an empirical way [44]. In this study, the zone of diagnostic uncertainty (3 - 4) corresponded to the lower limit of moderate pain scores (4), insuring that sheep suffering from moderate pain would be treated according to the cut-off point.”

Per my previous comment

I would imagine other scales were not classified in this manner because they are also non- continuous categorical scales used to assess whether or not pain is present.

This paragraph also highlights the completely arbitrary definition of “moderate pain” WSAVA Guidelines for the recognition, assessment, and treatment of pain describes the perceived pain of ovariohysterectomy in dogs and cats as “Moderate”.

I fully appreciate this is perceptions, and species differences can be vast. However:

The same references used in the surgical description (73) states mean pain score of ovariectomy by laparotomy as 5.6 (on a 10-pt scale, 0-9) and 0.3 out of 9 for both video- assisted and pure-laparoscopic ovariectomy.

Additionally, suggesting that this pain scale recommends that the readership should not treat “mild pain” (scores 0-3) takes away from the robust statistical approach of all the other steps in this instruments development.

I do not understand what this classification achieves.

Answer: Please see the previous comment about the same subject. Please also see the paragraph of discussion before Limitations (page 31, lines 25-27; page 32, lines 1-2).

Page 32, Line 17: “…video analysis does not necessarily equate to presential analysis.”

Consider: “…video analysis does not necessarily equate to in-person real-time analysis.”

Answer: Corrected to“…video analysis does not necessarily equate to in-person real-time analysis” (page 32, lines 18-19).

Page 32, Line 22: “According to a study by…”

Consider: “According to studies by our group…” since multiple references are used.

Answer: This part has been rephrased.
