# Supplementary material for: Validation of the Unesp-Botucatu composite scale to assess acute postoperative abdominal pain in sheep (USAPS)
Source: PLoS One. 2020 Oct 14;15(10):e0239622. doi: 10.1371/journal.pone.0239622 (PMC7556455; doi:10.1371/journal.pone.0239622)
Supplement: Attachment — Submitted filename: Response to Reviewer 1.docx [file pone.0239622.s011.docx]

**ManuscriptPONE-D-20-07213:**

**Validation of the Unesp-Botucatu composite scale to assess acute postoperative abdominal pain in sheep (USAPS)**

**REVIEWER: 1**

Dear Reviewer:

Thank you very much for the comments regarding this paper. All corrections have been performed according to your suggestions and each comment responded to separately.

We hope that after these corrections you consider the manuscript suitable for publication, but we are happy to answer any further questions.

Yours sincerely,

The authors

-----------------------------------------

Reviewers' comments:

Reviewer's Responses to Questions

**Comments to the Author**

1. If the authors have adequately addressed your comments raised in a previous round of review and you feel that this manuscript is now acceptable for publication, you may indicate that here to bypass the “Comments to the Author” section, enter your conflict of interest statement in the “Confidential to Editor” section, and submit your "Accept" recommendation.

Reviewer #1: All comments have been addressed

2. Is the manuscript technically sound, and do the data support the conclusions?

Reviewer #1: Yes

3. Has the statistical analysis been performed appropriately and rigorously?

Reviewer #1: Yes

4. Have the authors made all data underlying the findings in their manuscript fully available?

Reviewer #1: Yes

5. Is the manuscript presented in an intelligible fashion and written in standard English?

Reviewer #1: Yes

6. Review Comments to the Author

Reviewer #1: I applaud the authors for making the manuscript much clearer and much more concise, except for the discussion which still needs some work on improving the narrative and flow of the information.

Answer: The authors appreciate the effort taken to revise this manuscript. The manuscript is now more concise, and we have done our best to improve fluency. The pain intensity section was removed from all sections according to the Editor´s comments.

There are too many very short paragraphs and 'appetite' appears in many of them doted throughout the writing. This is the only section that really needs work.

Answer: The discussion is now more concise and clear. The comments about appetite were condensed.

I would also change figure 6 so that it doesn't have the colour 'blobs' around the boxes - I don't think they are helpful in getting your points across.

Answer: Corrected

In the limitations section, rather than 'pros' and 'cons' I would stick to advantages and disadvantages as proper, academic English.

Answer: Corrected (page 30, lines 3 and 6).

In addition on line 22 of the limitations you state "The scale was proven..." - I never like "proven" in scientific language as science never "proves" anything - consider instead 'demonstrate' or 'showed' etc.

Answer: The sentence has been rephrased (page 30, lines 16).

Otherwise - well done, much easier to read than before and see exactly what a good piece of work has been carried out.

.
