# Supplementary material for: Correction: Validation of the Unesp-Botucatu composite scale to assess acute postoperative abdominal pain in sheep (USAPS)
Source: PLoS One. 2022 May 5;17(5):e0268305. doi: 10.1371/journal.pone.0268305 (PMC9070906; doi:10.1371/journal.pone.0268305)
Supplement: S7 Table — (DOCX) [file pone.0268305.s001.docx]

**S7 Table. Contingency table between USAPS (0-12) and Numeric Scale (1-10) submitted to chi-square test (χ^2^=1741.6; df=108; p< 2.2^-16^). Occurrence ≥ 10 is highlighted in grey.**

|  | | **Numeric Scale** | | | | | | | | | |
| --- | --- | --- | --- | --- | --- | --- | --- | --- | --- | --- | --- |
|  |  | **1** | **2** | **3** | **4** | **5** | **6** | **7** | **8** | **9** | **10** |
| **USAPS** | **0** | 312 | 39 | 19 | 3 | 1 | 0 | 0 | 0 | 0 | 0 |
|  | **1** | 66 | 56 | 19 | 8 | 5 | 0 | 0 | 0 | 0 | 0 |
|  | **2** | 99 | 50 | 28 | 13 | 2 | 3 | 1 | 0 | 1 | 0 |
|  | **3** | 19 | 33 | 25 | 9 | 3 | 3 | 2 | 0 | 1 | 0 |
|  | **4** | 9 | 21 | 19 | 20 | 8 | 11 | 7 | 1 | 0 | 0 |
|  | **5** | 7 | 15 | 17 | 26 | 18 | 18 | 7 | 2 | 1 | 0 |
|  | **6** | 3 | 4 | 18 | 26 | 23 | 25 | 13 | 10 | 1 | 0 |
|  | **7** | 2 | 2 | 6 | 11 | 16 | 24 | 17 | 16 | 3 | 1 |
|  | **8** | 0 | 1 | 4 | 8 | 18 | 16 | 14 | 20 | 9 | 7 |
|  | **9** | 0 | 1 | 1 | 8 | 14 | 6 | 21 | 21 | 9 | 4 |
|  | **10** | 0 | 0 | 1 | 2 | 5 | 4 | 13 | 20 | 8 | 7 |
|  | **11** | 0 | 0 | 0 | 1 | 5 | 4 | 5 | 10 | 6 | 6 |
|  | **12** | 0 | 0 | 0 | 0 | 1 | 0 | 1 | 3 | 3 | 1 |
