# Supplementary material for: Correction: Validation of the Unesp-Botucatu composite scale to assess acute postoperative abdominal pain in sheep (USAPS)
Source: PLoS One. 2022 May 5;17(5):e0268305. doi: 10.1371/journal.pone.0268305 (PMC9070906; doi:10.1371/journal.pone.0268305)
Supplement: S8 Table — (DOCX) [file pone.0268305.s002.docx]

**S8 Table. Model findings with linear and quadratic fit of the predictor variable USAPS and with the predictive variable Numeric Scale.**

| Models | Estimate | Std. Error | t-value | P-value | AIC | BIC | Log Likelihood | R^2^ | R^2^-adjusted | |
| --- | --- | --- | --- | --- | --- | --- | --- | --- | --- | --- |
| Liner adjustment  (Intercept) | -0.045 | 0.0805 | -0.559 | 0.576 | 6298.03 | 6314.04 | -3146 | 0.70 | | 0.70 |
| Numeric Scale | 1.092 | 0.0183 | 59.555 | <2.2^-16^ |  |  |  | | | |
| Quadratic adjustment |  |  |  |  | 6236.05 | 6257.4 | -3114 | 0.71 | 0.71 | |
| (Intercept) | -0.8312 | 0.1253 | -6.633 | 4.54^-11^ |  |  |  | | | |
| Numeric Scale | 1.6596 | 0.0724 | 22.892 | < 2.2^-16^ |  |  |  | | | |
| Numeric Scale^2^ | -0.0630 | 0.0078 | -8.075 | 1.35^-15^ |  |  |  | | | |

AIC: Akaike information criterion; BIC: Bayesian information criterion.
